# The predicted sorting platform dynamically associates with the type III secretion system from *Xanthomonas euvesicatoria* in response to the external pH

**DOI:** 10.1186/s12866-025-04227-6

**Published:** 2025-08-25

**Authors:** Christian Otten, Daniela Büttner

**Affiliations:** 1 Institute of Biology, Department of Genetics, Martin-Luther University Halle- Wittenberg, Halle (Saale), Germany; 2 Present Address: Department of Microbial Pathogenesis, Yale University School of Medicine, New Haven, CT USA

**Keywords:** Type III secretion, Plant pathogen, Sorting platform, ATPase, Substrate Docking, AlphaFold 2, Alternative translation initiation

## Abstract

The Gram-negative bacterium *Xanthomonas euvesicatoria* (*Xe*) causes bacterial spot disease in pepper and tomato plants, and employs a type III secretion (T3S) system to translocate effector proteins into plant cells. The T3S system is a highly complex nanomachine which spans both bacterial membranes and is conserved in different bacterial species. In animal-pathogenic bacteria, structural and functional studies showed that the membrane-spanning secretion apparatus dynamically associates with a cytoplasmic sorting platform for substrate docking. The sorting platform is a wheel-like structure and contains the central ATPase SctN, which is connected via SctL to peripheral pod-like SctQ complexes. The architecture and function of a similar substructure of the T3S system in plant-pathogenic bacteria is still unknown. We previously reported that the SctQ homolog HrcQ from *Xe* forms complexes with its alternative translation initiation product HrcQ_C_, which stabilizes HrcQ and contributes to T3S. In the present study, we analysed the protein-protein interaction network of predicted sorting platform components from *Xe* and performed localization studies with HrcQ and the ATPase HrcN by fluorescence microscopy. We show that HrcQ complex formation depends on the SctL homolog HrcL which interacts with HrcQ and HrcN via its N- and C-terminal regions, respectively. Both HrcQ and HrcL contribute to HrcN stability, suggesting that a preassembled HrcQ-HrcL complex associates with and stabilizes the ATPase. Colocalization studies revealed that the HrcN-HrcL-HrcQ complex preferentially assembles at the membranes under T3S-permissive conditions when bacteria are cultivated at a low external pH. In contrast, at pH 7.0, HrcN and HrcQ form large cytoplasmic aggregates. Our data indicate that the interaction of the predicted sorting platform with the T3S system is influenced by external pH levels. These dynamic protein-protein interactions likely control the initiation of effector delivery in response to environmental stimuli, a strategy that might be also used by other plant pathogens.

**Supplementary Information:**

The online version contains supplementary material available at 10.1186/s12866-025-04227-6.

## Background

Many Gram-negative bacterial pathogens translocate effector proteins into eukaryotic host cells and thus manipulate cellular pathways such as defence responses to promote bacterial survival and proliferation [1–3]. Effector protein delivery often depends on a type III secretion (T3S) system which is present in many plant- and animal-pathogenic bacteria and is evolutionarily related to the bacterial flagellum [4, 5]. T3S systems span both bacterial membranes and are associated with an extracellular pilus-like appendage which serves as a transport channel for type III effectors (T3Es) to the host-pathogen interface [6]. Translocation of T3Es into target cells is mediated by a bacterial translocon which inserts into the eukaryotic plasma membrane [7]. At least nine components of T3S systems are conserved in plant- and animal-pathogenic bacteria, suggesting a similar architecture of the secretion apparatus in different bacterial species [8, 9]. Conserved core components of T3S systems from animal pathogens are designated Sct (secretion and cellular translocation) followed by a letter referring to the nomenclature of T3S system components from *Yersinia s*pp [9–11]. Cryo-electron microscopy (cryo-EM) and in situ cryo-electron tomography studies of single core components and isolated T3S systems revealed that Sct proteins are involved in the formation of oligomeric rings in the inner membrane (IM) and outer membrane (OM) [12, 13]. The OM ring is assembled by the secretin protein SctC, which is connected to 24mer oligomeric IM rings consisting of SctD on the outer and SctJ on the inner side [13]. The IM rings surround the export apparatus which is essential for protein transport across the IM. The export apparatus consists of a periplasmic SctR_5_-SctS_4_-SctT_1_ complex which associates with the transmembrane protein SctU and is embedded in a nonameric ring of SctV proteins [6, 14–19]. SctU and SctV span the IM and contain large cytoplasmic domains which are involved in substrate recruitment [13].

Additional substrate binding sites are provided by the cytoplasmic sorting platform which is a wheel-like protein complex and likely involved in the establishment of a T3S hierarchy [20]. Given its dynamic nature, the sorting platform from animal pathogens did not copurify with the T3S system and was, therefore, visualized by in situ cryo-electron tomography and superresolution microscopy [21–27]. Situated in the centre of the sorting platform is the hexameric ATPase SctN which is connected via a stalk-like structure consisting of SctO to the cytoplasmic domains of SctV proteins. Furthermore, SctN is linked via spoke-like structures formed by homodimers of SctL to six peripheral pods assembled by tetramers of SctQ proteins [13, 20, 28]. SctQ proteins were also designated C ring proteins due to their homology to FliM and FliN from the bacterial flagellum which form a ring-like cytoplasmic structure [29]. The C-terminal region of SctQ proteins corresponds to FliN and is often translated as separate protein (SctQ_C_) from an internal alternative translation start site [30–34]. SctQ_C_ proteins interact with and thus stabilize SctQ and were identified as essential structural components of the sorting platform in *Yersinia* spp. and *Shigella flexneri* [30–35].

The sorting platform attaches to the IM ring of the T3S system via SctK which links each of the six SctQ pods to the cytoplasmic domains of the 24mer IM ring component SctD [25–28, 36, 37]. In *Salmonella* spp., docking of the sorting platform leads to a conformational rearrangement of the cytoplasmic domain of SctD into six discrete patches [25]. Imaging in live bacteria by fluorescence microscopy revealed that the attachment of the sorting platform to SctD is a dynamic process, which is likely controlled by environmental conditions. Components of the sorting platform presumably preassemble in the cytoplasm and associate with SctD to activate T3S [20, 27, 34, 35, 38, 39]. In plant-pathogenic bacteria, however, the presence and function of a similar structure of the T3S system is still unknown.

In the present study, we analysed predicted sorting platform components from the plant-pathogenic bacterium *Xanthomonas euvesicatoria* (*Xe*, formerly known as *Xanthomonas campestris* pv. *vesicatoria*), which is the causal agent of bacterial spot disease in pepper and tomato and a model organism for the analysis of bacterial virulence strategies in plants [40, 41]. The T3S system from *Xe* translocates at least 36 T3Es into plant cells where they interfere with various cellular processes to promote bacterial invasion and multiplication in the plant apoplast [3, 42, 43]. In resistant plants, which contain corresponding resistance genes, individual T3Es are recognized by the plant immune system and often trigger the hypersensitive response (HR), a local defence response at the infection site [44, 45]. Given the essential contribution of T3S genes to T3E delivery and thus to the interaction with susceptible and resistant plants, they were designated *hrp* (hypersensitive response and pathogenicity) [46]. *hrp* genes from *Xe* comprise 25 genes organized in eight operons in a chromosomal gene cluster. Two regulators, HrpG and HrpX, are encoded outside the T3S gene cluster and activate *hrp* gene expression when the bacteria enter the plant or are cultivated in special minimal media [46–48]. Eleven *hrp* gene products are homologous to corresponding T3S system components from plant- and/or animal-pathogenic bacteria and were, therefore, named Hrc for Hrp conserved followed by a letter which refers to the corresponding Sct proteins from animal pathogens [9]. In addition to Hrc proteins, the function and assembly of the T3S system from *Xe* depends on non-conserved Hrp proteins, which are only present in *Xe* or related plant pathogens. Several Hrp proteins are themselves secreted and constitute extracellular components of the T3S system such as the pilus protein HrpE and the translocon protein HrpF [49, 50]. Additional Hpa (Hrp associated) proteins encoded in the *hrp* gene cluster contribute to pathogenicity and include the T3S chaperone HpaB, which is required for efficient T3E delivery [51, 52].

The mechanisms underlying the recognition and docking of T3S substrates to the secretion apparatus are largely unknown. Targeting of substrates to the T3S system often depends on N-terminal export signals which are part of unstructured protein regions and are not conserved on the amino acid level [10, 53–56]. In *Xe*, possible T3S substrate docking sites include the ATPase HrcN and the SctQ homolog HrcQ, which are both components of the predicted sorting platform [57, 58]. We previously showed that HrcQ from *Xe* is stabilized by its C-terminal region, HrcQ_C_, which is translated as separate protein from an internal alternative translation initiation codon as was reported for SctQ_C_ proteins from animal-pathogenic bacteria [30–34, 59, 60]. A complex of HrcQ_C_ and HrcQ associates with the SctK-like protein HrpB4 which acts as a linker to the cytoplasmic domain of the IM ring component HrcD [37, 59].

HrcQ complex formation was visualized in *Xe* by fluorescence microscopy analysis of a HrcQ-sfGFP (superfolder green fluorescent protein) fusion protein which forms fluorescent foci at the cell periphery [59, 61]. Genetic approaches showed that foci formation depends on a functional T3S system and on the cytoplasmic domain of the IM ring component HrcD which provides the docking sites for the predicted sorting platform [37, 59]. In the absence of HrcD, HrcQ-sfGFP forms large fluorescent complexes in the bacterial cytoplasm [59]. The formation of cytoplasmic but not of membrane-associated HrcQ complexes was independent of HrpB4, suggesting that HrpB4 is specifically required for the attachment of HrcQ to the T3S system [37]. It is, however, still unknown whether HrcQ is part of the predicted T3S sorting platform and associates with the ATPase complex in the bacterial cytoplasm. In the present study, we, therefore, examined potential interactions of HrcQ with the ATPase HrcN and its predicted regulator HrcL and evaluated their roles in HrcQ complex formation using fluorescence microscopy. Our experimental findings and the results of structural modeling indicate that the N- and C-terminal regions of HrcL link the HrcQ/HrcQ_C_ complexes to the ATPase HrcN. Furthermore, our data suggest that a preassembled HrcQ-HrcL complex is required for the recruitment and stabilization of HrcN. Notably, the predicted sorting platform specifically associates with the T3S system under T3S-permissive conditions. We, therefore, propose that the HrcN-HrcL-HrcQ complex dynamically interacts with the T3S system in response to environmental conditions to regulate T3S in *Xe*.

## Methods

### Bacterial strains and growth conditions

Plasmids were introduced into *Escherichia coli* and *Xe* using either electroporation or triparental conjugation and pRK2013 as a helper plasmid. *E. coli* strains were cultivated at 37 °C in lysogeny broth (LB) medium and *Xe* strains at 30 °C in nutrient-yeast extract-glycerol (NYG) medium or in minimal medium A (MA; 12 mM K_2_HPO_4_, 6.6 mM KH_2_PO_4_, 1.52 mM (NH_4_)_2_SO_4_, 0.34 mM Na-citrate*2H_2_O, 1 mM MgSO_4_). MA medium was supplemented with sucrose (10 mM) and casamino acids (0.3%) at pH 5.3 or pH 7.0 [62] and antibiotics were added to the media at the following final concentrations: ampicillin, 100 µg/ml; kanamycin, 25 µg/ml; rifampicin, 100 µg/ml; spectinomycin, 100 µg/ml, gentamycin, 15 µg/ml and streptomycin, 25 µg/ml. All plasmids and bacterial strains, which were used in this study, are summarized in Table S1.

### Plant material and plant infections

Bacteria were infiltrated with a needle-free syringe into leaves of the near-isogenic pepper cultivars Early Cal Wonder (ECW) and ECW-10R. If not stated otherwise, bacterial solutions were adjusted in 1 mM MgCl_2_ to an optical density at 600 nm of 0.1 (corresponding to 1 × 10^8^ colony-forming units (CFU) ml^-1^). After infection, plants were incubated in growth chambers for 16 h of light at 28 °C and 8 hours of darkness at 22 °C. Disease symptoms and the HR were photographed over a period of one to seven days post inoculation (dpi). While disease symptoms including water-soaking and yellowing are best observed in photographs, infected leaves of resistant plants were bleached over night at 65 °C in 70% ethanol to enhance HR visualization. All infection experiments were performed at least three times with consistent results, and one representative image is shown.

### Protein-protein interaction studies using the BACTH system

For BACTH (bacterial adenylate cyclase-based two-hybrid) assays, expression constructs encoding T25 and T18 fusion proteins were introduced into chemically competent cells of the *E. coli* reporter strain DHM1. To select cells with both plasmids, transformants were plated on LB medium containing kanamycin and gentamycin. For each plasmid combination, three transformants were grown in 500 µl of LB medium containing both antibiotics. Cultures were incubated in 96 well plates overnight at 30 °C with shaking. Subsequently, 2 µl of each culture were spotted on LB indicator plates containing kanamycin, gentamycin, X-gal (5-bromo-4-chloro-3-indolyl-β-D-galactopyranoside; 40 µg/ml) and IPTG (isopropyl-β-D-thiogalactopyranoside, final concentration of 2 mM). The indicator plates were incubated for five to six days at room temperature in the dark before the colonies were photographed. As negative control, every fusion protein was tested against the T25 or the T18 domain alone. Each interaction assay was conducted at least three times with transformants derived from independent transformations. One representative colony is shown.

For protein detection, T18 and T25 expression constructs were transferred into the *cya*^*+*^ *E. coli* strain JM109, in which recombination is minimized (Table S1). JM109 cultures were grown in LB medium and gene expression was induced with IPTG at an optical density at 600 nm (OD_600_) of 0.6–0.8. After two hours of induction at 37 °C, cells from 500 µl of the cultures were collected by centrifugation, resuspended in Laemmli buffer and analysed by immunoblotting, using a FLAG epitope-specific antibody. Since cAMP/CAP is required for optimal activation of the *lac* promoter which drives the expression of T25 and T18 reporter fusions, protein synthesis is analysed in *cya*^*+*^ *E. coli* strains instead of the *cya* mutant and reporter strain DHM1, which is used for the BACTH assays.

### Immunoblot analyses of ***Xe*** cell extracts

To detect HrcQ, HrcN and HrcJ in *Xe* strains, bacteria were grown overnight at 30 °C in minimal medium A (pH 7.0) without antibiotics on a rotary shaker. Cells were harvested from 500 µl of the cultures by centrifugation and resuspended in 50 µl of Laemmli buffer. Equal amounts of cell extracts adjusted according to the OD_600_ were subjected to SDS-PAGE and analysed by immunoblotting, using antibodies directed against HrcN, HrcQ, the IM ring component HrcJ and the c-Myc epitope (polyclonal antibody from rabbit, Sigma-Aldrich) [63]. Horseradish peroxidase-labelled anti-rabbit antibodies served as secondary antibodies and binding of antibodies was visualized with a chemiluminescence imager (Vilber Fusion FX Edge). HrcN- and HrcQ-specific signals were quantified using the open-source image processing tool Fiji/ImageJ2 [64]. For this, the immunoblot images were transformed via Affinity Photo into “Grayscale” and JPEG files. For each blot, in which the analysed proteins (HrcQ and HrcN) and the loading control (HrcJ) were detected, an ROI (region of interest) frame covering the minimal area of the largest band was defined and the intensity of each signal was measured (the “Set measurements” were set to “Grey Mean Value”). With the same ROI frames, a background measurement was performed by moving the frames into areas without any signals above or below the detected proteins. All data were exported to Excel and the relative values of the signals were calculated. For this, the pixel density was inverted using the formula 255– X, in which X is the value recorded by Fiji. For the determination of the net value of each signal, the inverted background value was deducted. The final relative values were calculated as ratio of the net values of the respective HrcQ and HrcN signal and the HrcJ loading control.

### Fluorescence microscopy analyses

For the analysis of fluorescent reporter fusions, *Xe* strains containing the respective modular T3S gene cluster constructs were grown in MA medium (pH 7.0) supplemented with sucrose (10 mM) and casamino acids (0.3%) without antibiotics. Following overnight cultivation, the cells were resuspended at an OD_600_ of 0.15 in MA medium containing BSA and thiamine, adjusted to pH 5.3 or pH 7.0. The cultures were then incubated at 30 °C on a tube rotator for at least 1 h. For microscopy, bacterial cultures were subsequently spotted onto a slide overlaid with a 1.5% agarose pad prepared with MA medium at pH 5.3 or pH 7.0, following a previously described protocol [61]. Fluorescence analysis was performed using a confocal laser scanning microscope (Leica STELLARIS 8) equipped with a 60 x magnification objective and 5 x digital magnification. Fluorophores were excited using a white light laser at wavelengths specific for each fluorophore (485 nm for sfGFP and 551 nm for mKOκ), and fluorescence signals were captured using HyD S detectors with emission detection windows optimized for the fluorophores (sfGFP at 510 nm and mKOκ at 563 nm). Pictures were taken from approximately 500–1000 cells of each of the three transconjugants for every strain. Foci were counted in a single z-plane. Image analysis across multiple z-planes confirmed that minimal to no foci were lost, ensuring that restricting the analysis to one z-plane would not affect the results (Fig. S14). To enhance or sharpen fine structures and details of sfGFP-HrcN in the colocalization studies, the images were processed using the Leica LIGTHNING detection concept. Fig. S12 exemplifies for each fluorescence image the foci with the weakest fluorescence which were included in the quantification. Foci were manually counted using the Multi-Point-Tool of the ImageJ2 processing package Fiji, thus ensuring accuracy and preventing errors like missed or miscounted foci that could arise from manual note-taking [64]. For the analysis of protein synthesis, bacteria incubated in MA medium as described above were harvested by centrifugation and resuspended in Laemmli buffer. Equal amounts of the cell extracts adjusted according to the OD_600_ were analysed by SDS-PAGE and immunoblotting using GFP- (Roche Applied Science), FLAG- (Sigma-Aldrich), HrpB1- and HrcC-specific antibodies [62, 65].

### Generation of expression constructs by Golden Gate cloning

For the generation of *sfgfp*, *hrcL*, *hrcN* and *hrcQ* expression constructs, the corresponding genes or deletion derivatives thereof were amplified by PCR and respective primers (listed in Table S2), and the amplicons were inserted via blunt-end cloning using *Sma*I and ligase into vector pICH41021. After Sanger sequencing, individual inserts were cloned downstream of the native *hrpB* promoter comprising 113 bp upstream of the ATG start codon of *hrpB1* into vector pBRM-P or downstream of the *lac* promoter into vector pBRM or the BACTH vectors pUT18_GG_, pUT18C _GG_, pKT25_GG_ and pKNT25 _GG_ using the Golden Gate cloning technique. For this, cut-ligation reactions containing vector and insert were incubated with *BsaI* and ligase in a thermocycler for 55 cycles at 37 °C for 2 min and 16 °C for 5 min followed by 10 min incubation at 50 °C and 20 min incubation at 65 °C [66]. *E. coli* Top10 or JM109 cells were used for subsequent transformation of the reactions and transformants were analysed by test restriction of isolated plasmids.

### Generation of modular T3S gene cluster constructs

Modular T3S gene cluster constructs were generated by Golden Gate-based modular cloning (MoClo) as described previously [61, 67]. For this, promoter and ORF modules were assembled using a series of MoClo vectors (designated level − 2, -1, 0, 1, M and P) which allow the stepwise cloning of multigene constructs by alternate use of the type IIs enzymes *Bsa*I and *Bpi*I [67]. The MoClo system was previously used to assemble the T3S gene cluster, the regulatory and accessory genes hrpG*,* hrpX*,* xopA* and *hpaH* as well as dummy modules, which can be replaced by reporter fusions [61].

#### Mutation of single or multiple T3S genes in the modular T3S gene cluster expression constructs

In the present study, we introduced deletions into single T3S genes by replacing the corresponding gene modules in the modular T3S gene cluster constructs. To generate a deletion in *hrcL*, the 5’ and 3’ regions of *hrcL* were amplified by PCR using construct pAGB192 (*hrcL*) as template and primer pairs hrcL-MoClo-F1/hrcL-Del-MoClo-R1 and hrcL-Del-MoClo-F2/hrcL-MoClo-R2. The resulting amplicons were assembled in vector pAGM9121 using *Bpi*I and ligase, thus generating the level − 2 construct pAGB957 (∆*hrcL*). The corresponding insert was subsequently assembled with the inserts of constructs pAGB193 (*hrcN*), pAGB194 (*hrpB7*), pAGB195 (*hrcT*) and pAGB196 (*hrcC*) in vector pAGM1311, using *Bsa*I and ligase, thus leading to the level − 1 construct pAGB961. The inserts of pAGB961 and pAGB197 (*hrpB* operon promoter + *hrpB1* to *hrpB4*) were then cloned into vector pICH41331 using *Bpi*I and ligase, thus generating the level 0 construct pAGB965 (*hrpA* and *hrpB* operon + ∆*hrcL*). Next, the insert of pAGB965 was transferred into the *Bsa*I sites of pICH47811 to generate the level 1 construct pAGB969. For the assembly of the modular level M T3S gene cluster construct pAGB979 containing the deletion in *hrcL*, the inserts of pICH54011 (dummy pos. 1), pAGB969 (*hpaA* to *hrpB* operons and ∆*hrcL* fragment), pAGB155 (*hrpC* to *hpaB* operons), pAGB156 (*hrpF* operon) and pICH50900 (end linker 4) were cloned into vector pAGM8031 using *Bpi*I and ligase. For localization studies with HrcQ-sfGFP, an additional deletion in *hrcQ* was introduced during the assembly by replacing construct pAGB155 with construct pAGB275 (*hrpC* to *hpaB* operons + ∆h*rcQ*), thus resulting in the level M construct pAGB973.

For the introduction of a deletion in *hrcN*, the 5’ and 3’ regions flanking the deletion were amplified by PCR using phosphorylated primers (hrcN-Del-MoClo-F/hrcN-Del-MoClo-R) and the level − 2 plasmid pAGB193 (*hrcN*) as template. The amplicon was ligated to generate the level − 2 plasmid pAGB514 (∆*hrcN*). The corresponding insert was subsequently assembled with the inserts from constructs pAGB192 (*hrcL*), pAGB194 (*hrpB7*), pAGB195 (*hrcT*) and pAGB196 (*hrcC*) in vector pAGM1311, using *Bsa*I and ligase. The resulting level − 1 construct pAGB674 was ligated together with the insert of pAGB197 (containing the *hrpB* operon promoter as well as *hrpB1* to *hrpB4)* using *Bpi*I and ligase to generate the level 0 construct pAGB678 (*hrpA* and *hrpB* operon including ∆*hrcN*). The insert of pAGB678 was ligated into the *Bsa*I sites of vector pICH47811 to generate the level 1 construct pAGB681. The final level M construct pAGB737 containing the T3S gene cluster with a deletion in *hrcN* was generated by assembly of constructs pICH54011 (dummy pos. 1), pAGB681 (*hpaA* to *hrpB* operons including ∆*hrcN*), pAGB155 (*hrpC* to *hpaB* operons), pAGB156 (*hrpF* operon) and pICH50900 (end-linker 4) in vector pAGM8031 using *Bpi*I and ligase. To introduce an additional deletion in *hrcQ*, which was necessary for the localization studies with HrcQ-sfGFP, the insert of pAGB155 was replaced by the insert of pAGB275 (*hrpC* to *hpaB* operons including ∆*hrcQ*).

To introduce deletions in both *hrcN* and *hrcL*, the last 46 bp of *hrcN* were amplified by PCR using primers hrcNL-Del-MoClo-F and hrcNL-Del-MoClo-R and pAGB193 (*hrcN*) as template. The resulting amplicon was cloned into vector pAGM9121 using *Bpi*I and ligase to obtain the level − 2 construct pAGB659 (∆*hrcN*/*hrcL*). The insert of pAGB659 was assembled with the inserts of constructs pAGB194 (*hrpB7*), pAGB195 (*hrcT*) and pAGB196 (*hrcC*) into vector pAGM1311 using *Bsa*I and ligase, thus resulting in the level − 1 construct pAGB660. Subsequent assembly of pAGB660 with pAGB197 (*hrpB* operon promoter and *hrpB1* to *hrpB4*) in vector pICH41331 using *Bpi*I and ligase led to the level 0 construct pAGB661. The insert of pAGB661 was transferred to vector pICH47811 using *Bsa*I and ligase to generate the level 1 construct pAGB662. The final level M construct pAGB666 containing deletions in *hrcN* and *hrcL* resulted from assembly of constructs pICH54011 (dummy pos. 1), pAGB662 (*hpaA* to *hrpB* operons including ∆*hrcN/hrcL*), pAGB155 (*hrpC* to *hpaB* operons), pAGB156 (*hrpF* operon) and pICH50900 (end-linker 4) with *Bpi*I and ligase in vector pAGM8031. For the additional deletion of *hrcQ*, construct pAGB155 was replaced by construct pAGB275 (*hrpC* to *hpaB* operons including ∆*hrcQ*).

To mutate both *hrcN* and *hrpB4* in the modular T3S gene cluster, the inserts of constructs pAGB776 (contains the *hrpB* operon promoter and *hrpB1* to *hrpB4* with a frameshift mutation in *hrpB4*) and pAGB674 (contains *hrcC* and *hrcL* to *hrcT* including ∆*hrcN*) were assembled using *Bpi*I and ligase to generate the level 0 construct pAGB1270 and were subsequently transferred to vector pICH47811 using *Bsa*I and ligase, leading to the level 1 construct pAGB1273. A level M construct (pAGB1275) containing the T3S gene cluster with mutations in *hrcN* and *hrpB4* was generated by assembly of pAGB1275 with pICH54011 (dummy pos. 1), pAGB155 (*hrpC* to *hpaB* operons), pAGB156 (*hrpF* operon) and pICH50900 (end-linker 4) using *Bpi*I and ligase in vector pAGM8031. For the generation of the *hrcN*, *hrcL* and *hrpB4* triple mutant, the inserts of pAGB776 (*hrpB* operon promoter and *hrpB1* to *hrpB4* including *fshrpB4*) and pAGB660 (*hrcC* and *hrcL* to *hrcT* including ∆*hrcN/hrcL*) were cloned into vector pICH41331 using *Bpi*I and ligase to obtain the level 0 construct pAGB1201 (*hrpA* and *hrpB* operon + ∆*hrcN/hrcL/*fs*hrpB4*). The insert of pAGB1201 was transferred into vector pICH47811 using *Bsa*I and ligase to generate the level 1 construct pAGB1202 and subsequently assembled with the inserts of pICH54011 (dummy pos. 1), pAGB1202 (*hpaA* to *hrpB* operons including ∆*hrcN/hrcL/*fs*hrpB4*), pAGB155 (*hrpC* to *hpaB* operons), pAGB156 (*hrpF* operon) and pICH50900 (end linker 4) using *Bpi*I and ligase in vector pAGM8031, leading to the level M construct pAGB1394. To introduce an additional deletion in *hrcQ*, pAGB155 was replaced by pAGB275 (*hrpC* to *hpaB* operons including ∆*hrcQ*) to generate the level M construct pAGB1203. All constructs are listed in Table S1. Sequences with the following GenBank accession numbers were used to generate the expression constructs and gene modules listed in Table S1: hrcC (APO92110), hrpB1 (AAB08456), *hrpB2* (AAB08457), *hrcJ* (NEK90484), *hrpB4* (AAB08459), *hrcL* (CAJ22062), *hrcN* (MG847397), *hrpB7* (AAB08462), *hrcT* (NEL30510), *hrcU* (NEK90481), *hrcV* (KLA79882), *hpaC* (CAJ22055), *hrcQ* (AAD21320), *hrcR* (AAD21321), *hrcS* (AAD21322), *hpaA* (AAD21323), *hrcD* (CAJ22050), *hrpD6* (AAD21324), *hrpE* (DQ286694), *hpaB* (AAD21328), *hpaE* (CAJ22046), *hrpF* (AAB86527), *xopA* (AAL78294), *hpaH* (AAL78295), *hrpG* (U57625) and *hrpX* (AB026466).

#### Generation of reporter fusions

The *hrcQ-sfgfp* fusion was generated as described previously and assembled with *xopA*,* hpaH*,* hrpX* and hrpG* in the level M vector pAGM8079 to generate the level M construct pAGB322 [61]. For localization studies, pAGB322 was assembled with the level M constructs pAGB762 (*hrp* gene cluster + ∆*hrcN/∆hrcQ*), pAGB973 (*hrp* gene cluster + ∆*hrcL/∆hrcQ*), pAGB764 (*hrp* gene cluster + ∆*hrcN/L/∆hrcQ*) or pAGB1203 (*hrp* gene cluster + ∆*hrcN/L/∆hrcQ/fshrpB4*) (see above) to generate the corresponding level P constructs pAGB765, pAGB990, pAGB767 and pAGB1205, respectively (see Table S1). For the generation of a *hrcQ*-*mKO*κ expression cassette, *hrcQ* was amplified by PCR using primers hrcQ-NTM-MoClo-F and hrcQ-NTM-MoClo-R and the resulting amplicon was cloned into vector pICH41021 using *Sma*I and ligase, resulting in the level − 1 construct pAGB805 (NTM [N-terminal]-*hrcQ*). *NTM*-*hrcQ* was subsequently assembled with the inserts of pAGB1000 (2 x AKLEGPAGL), which encodes a two-fold linker sequence, and pAGB1153 (CTM [C-terminal]-*mKO*κ) in pICH41308 using *Bpi*I and ligase, thus generating the level 0 construct pAGB1172. Assembly of pAGB1172 (*hrcQ-2 x AKLEGPAGL-mKO*κ) with pAGB249 (*hrpD* operon promoter) and pAGB231 (*Xe term1*) in the level 1 destination vector pICH4778 using *Bsa*I and ligase led to the level 1 construct pAGB1173. In the following cloning step, the inserts of pAGB1173, pAGB157 (*xopA + hpaH*), pAGB160 (*hrpX*), pAGB163 (hrpG*), pICH54022 (dummy pos. 2’) and pICH50881 (end linker 2) were cloned into the level M vector pAGM8079 using *Bsa*I and ligase, thus generating the level M construct pAGB1184. In the final step, assembly of pAGB1184 with pAGB273 (T3S gene cluster including ∆*hrcQ*) and pICH79264 (level P end-linker 2) in the *Bsa*I sites of pICH75322 led to the level P construct pAGB1185.

To generate N-terminal translational fusions to HrcN, the corresponding gene was amplified by PCR using primers hrcN-CTM-MoClo-F and hrcN-CTM-MoClo-R and the amplicon was cloned into vector pAGM9121 to generate the level − 2 construct pAGB608 (CTM-*hrcN*). The insert of pAGB608 was cloned into vector pAGM1311 using *Bsa*I and ligase to generate the level − 1 construct pAGB478 (*CTM*-*hrcN*). pAGB478 was then assembled with pAGB488 (*NTM*-*sfgfp*, includes the linker AKLEGPAGL) in vector pICH41308 using *Bpi*I and ligase to generate the level 0 construct pAGB831. The insert of pAGB831 (*sfgfp-hrcN*) was subsequently ligated with the inserts of pAGB512 (*hrpB* operon promoter) and pAGB231 (*Xe term1*) into the level 1 destination vector pICH47781, using *Bsa*I and ligase to generate the level 1 construct pAGB839. For the generation of the level 0 construct pAGB232 (*Xe term2*), which encodes a native *Xe* translational terminator, the corresponding terminator sequence was amplified by PCR using primers termX2-MoClo-F and termX2-MoClo-R and chromosomal DNA from *Xe* as template. The resulting amplicon was cloned into vector pICH41276 using *Bpi*I and ligase to generate the level 0 construct pAGB232. The level 1 construct pAGB839 containing the *hrpB* operon promoter, *sfgfp-hrcN* and *Xe term2* was assembled with pAGB157 (*xopA + hpaH*), pAGB160 (*hrpX*), pAGB163 (hrpG*), pICH54022 (dummy pos. 2’) and pICH50881 (end-linker 2) in the level M vector pAGM8079 using *Bpi*I and ligase. The resulting level M construct pAGB847 was subsequently assembled with pAGB737 (*hrp* gene cluster + ∆*hrcN*) and pICH79264 (level P end linker 2) in the *Bsa*I site of pICH75322 to generate the level P construct pAGB855.

For colocalization studies with sfGFP-HrcN and HrcQ-mKOκ, pAGB831 (*sfgfp-hrcN*), pAGB512 (*hrpB* operon promoter) and pAGB232 (*Xe term2*) were assembled in the additional level 1 destination vector pICH47742, thus generating the level 1 construct pAGB1342, which was subsequently assembled with the inserts of pAGB1173 (*hrcQ-2 x AKLEGPAGL-mKO*κ), pAGB157 (*xopA + hpaH*), pAGB160 (*hrpX*), pAGB163 (hrpG*) and pICH50881 (end-linker 2) in vector pAGM8079 using *Bpi*I and ligase, resulting in the level M construct pAGB1343. To generate the final level P constructs, the inserts of pAGB1343 (*hrcQ-2 x AKLEGPAGL-mKO*κ + *sfgfp-hrcN*) and pICH79264 (level P end linker 2) were assembled with pAGB762 (*hrp* gene cluster + ∆*hrcN/*∆*hrcQ*) or with pAGB764 (*hrp* gene cluster + ∆*hrcN/L/*∆*hrcQ*) in the *Bsa*I site of vector pICH75322 to obtain the level P constructs pAGB1344 and pAGB1376, respectively.

To generate an N-terminal translational fusion to HrcL, *hrcL* was amplified by PCR using primers hrcL-CTM-MoClo-F and hrcL-CTM-MoClo-R and the resulting amplicon was cloned into vector pICH41021 using *Sma*I and ligase to obtain the level − 2 plasmid pAGB1118 (*CTM*-*hrcL*). The insert of pAGB1118 was transferred into the *Bsa*I sites of vector pAGM1311 to generate the level − 1 construct pAGB1119 (*CTM*-*hrcL*). The module for an N-terminal translational fusion of sfGFP to the target protein, *sfgfp* was amplified (sfGFP-NTM-woLink-F/ sfGFP-NTM-woLink-R) and the PCR product cloned into pICH41021 with *Sma*I and ligase to obtain the level − 1 plasmid pAGB1047 (NTM-*sfgfp*). For the generation of the linker (2 x GGAGGAGG) for the next cloning step, the two primers MoClo-LinkDiepold-5-3 and MoClo-LinkDiepold-3-5 were annealed and cloned with *Sma*I and ligase into vector pICH41021 to generate the level − 1 construct pAGB1001. The inserts of constructs pAGB1001 (2 x GGAGGAGG), pAGB1119 (CTM-*hrcL*) and pAGB1047 (NTM-*sfgfp*) were assembled in vector pICH41308, using *Bpi*I and ligase and generating the level 0 construct pAGB1121. The inserts of pAGB1121 (*sfgfp- 2x GGAGGAGG-hrcL*), pAGB512 (*hrpB* operon promoter) and pAGB232 (*Xe term2*) were subsequently assembled in the level 1 destination vector pICH47781 using *Bsa*I and ligase, leading to the level 1 construct pAGB1122. The corresponding insert was ligated with the insert of pAGB157 (*xopA + hpaH*), pAGB160 (*hrpX*), pAGB163 (hrpG*), pICH54022 (dummy pos. 2’) and pICH50881 (end-linker 2) into the *Bpi*I sites of the level M vector pAGM8079, resulting in the level M construct pAGB1124. The final level P construct for the complementation studies was assembled by cloning the inserts of pAGB1124, pAGB979 (*hrp* gene cluster + ∆*hrcL*) and pICH79264 (level P end-linker 2) into the level P vector pICH1126, using *Bsa*I and ligase, thus obtaining the level P construct pAGB1126.

### Structure predictions using AlphaFold 2

For the generation of a *Xe* sorting platform model, single structures and complex predictions were performed using the AlphaFold2 notebook from ColabFold with default parameters [68]. The predictions were individually performed for HrcD_N_ (HrcD_1 − 100_), HrpB4, HrcQ, HrcQ_C_, HrcL, HrcN and HrpB7. To generate the hexameric ATPase complex consisting of HrcN and the predicted stalk protein HrpB7, the predicted models were superimposed onto the hexameric EscN structure with the associated EscO stalk (PDB: 6NJP) [69] using UCSF ChimeraX [70–73]. For the modelling of the remaining components of the sorting platform, the multimeric complexes 2×HrcD_N_-HrpB4, HrpB4-HrcQ, HrcQ-2×HrcQ_C_, HrcQ-HrcL, HrcL-HrcN were individually predicted using AlphaFold2 and the overlapping protein structures were subsequently superimposed (e.g. 2×HrcD_N_-HrpB4, HrpB4-HrcQ) to assemble a pod structure subunit (2×HrcD_N_-HrpB4-HrcQ-2×HrcQ_C_-HrcL-HrcN). This subunit was then superimposed onto the predicted HrcN_6_-HrpB7 complex using the structure of a HrcN monomer to generate the wheel-like sorting platform. The amino acids which form the interaction interfaces between HrcQ-HrcL and HrcL-HrcN were predicted with the “select → contact” command in UCSF ChimeraX and labelled with a different colour. The structures of deletion derivatives of HrcN and HrcL were predicted accordingly. The *Xe* proteins with the following accession numbers were analysed: HrcD (CAJ22050), HrpB4 (AAB08459), HrcQ (AAD21320), HrcN (CAJ22063), HrcL (CAJ22062) and HrpB7 (AAB08462).

## Results

### Predicted structure of the sorting platform from ***Xe***

**Fig. 1:**
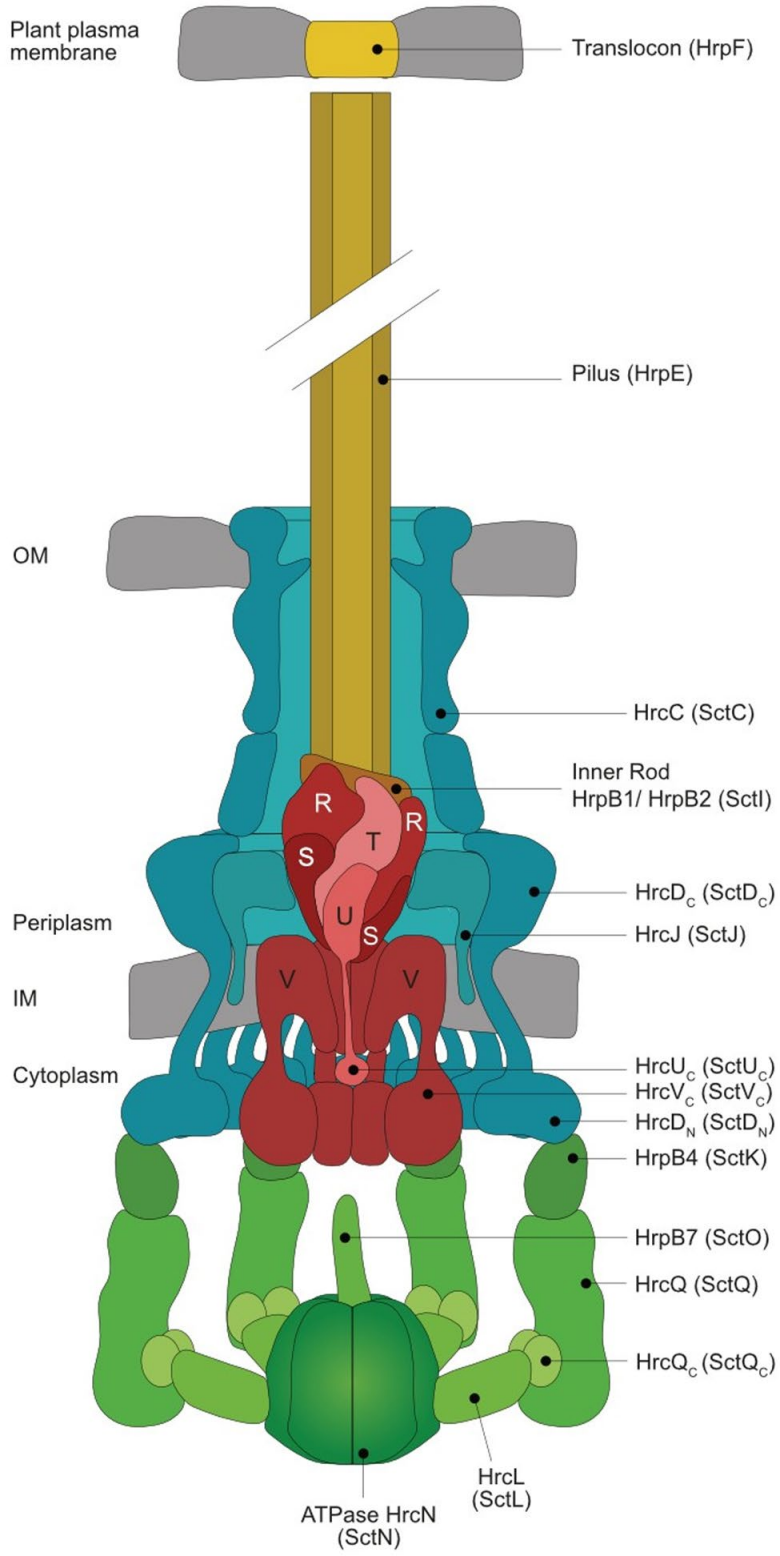
Model of the T3S system from *Xe*. The T3S system from *Xe* spans both bacterial membranes and is associated with a predicted cytoplasmic sorting platform consisting of the central ATPase HrcN which associates via the linker protein HrcL with six pod-like structures assembled by HrcQ and HrcQ_C_. The sorting platform binds via HrpB4 to the cytoplasmic domains of the IM ring protein HrcD. The IM rings surround the export apparatus which consists of HrcU, HrcR, HrcS, HrcT and HrcV and likely provides the assembly platform for the periplasmic inner rod structure composed of HrpB1 and HrpB2. The membrane-spanning secretion apparatus is associated with an extracellular pilus structure composed of HrpE. A channel-like translocon assembled by HrpF likely inserts into the plant plasma membrane. The sorting platform is shown in green, the export apparatus in red and the IM and OM rings in blue. Letters refer to conserved Hrc proteins. Corresponding Sct proteins of T3S system from animal-pathogenic bacteria are written in brackets. IM, inner membrane; OM, outer membrane

**Fig. 2:**
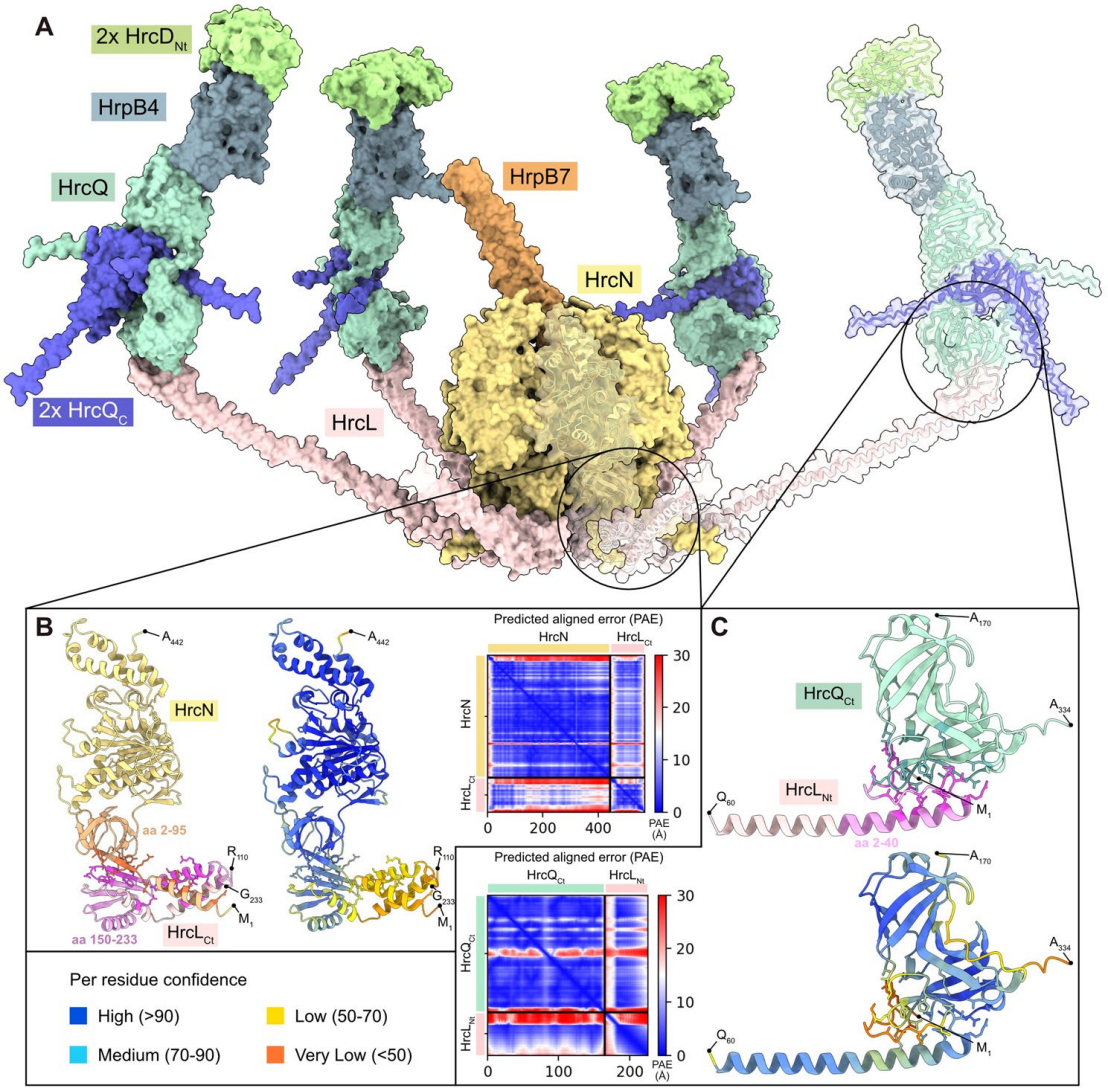
Predicted structure of the putative sorting platform from *Xe*. (**A**) Structural modelling of the predicted sorting platform using the AlphaFold 2 algorithm and the molecular visualization program UCSF ChimeraX. Acentral hexamer of the ATPase HrcN (yellow) is attached to the SctO-like protein HrpB7 (orange) and associated via the linker protein HrcL (pink) to pod-like structures consisting of HrcQ (turquoise) which is associated with a dimer of its alternative translation product HrcQ**A**_C_ (blue). HrcQ attaches via the SctK-like protein HrpB4 (light blue) to a dimer of the cytoplasmic domain of the IM ring component HrcD (green). (**B**) Predicted interaction sites between HrcN and the C-terminal region of HrcL (HrcL_Ct_). Structures of HrcN and HrcL_Ct_ were predicted by AlphaFold 2 and numbers refer to amino acid positions. Different colouring reflects the “per residue confidence” score (pLDDT) as indicated. A predicted aligned error (PAE) plot shows regions of high (blue colour, low PAE value) and low (red colour, high PAE value) confidence for the predicted structures. (**C**) Predicted interaction sites between the N-terminal region of HrcL (HrcL_Nt_) and the C-terminal region of HrcQ (HrcQ_Ct_). The “per residue confidence” and the PAE are indicated as described in (**B**)

Structural studies in animal-pathogenic bacteria showed that SctQ proteins assemble at the periphery of the sorting platform [13, 20, 23]. In situ visualization by cryo-electron tomography in *Salmonella* and *Shigella* spp. revealed that the sorting platform is a hexagonal cage-like structure and consists of the central ATPase SctN which is connected through SctL with pod-like structures containing SctQ and dimers of SctQ_C_ [24, 25, 28, 74]. A similar architecture is predicted for the T3S system and the corresponding Hrc proteins from *Xe* (Fig. 1). Our previous in vitro pull-down experiments with corresponding proteins from *Xe* revealed that the ATPase HrcN interacts with itself as well as with HrcL and HrcQ [57, 58]. HrcN is conserved in different bacterial species while HrcL and HrcQ only share weak sequence similarities with SctL and SctQ from animal-pathogenic bacteria (0–35% amino acid identity). To predict the structure of the HrcN-HrcL-HrcQ complex in association with the SctO-like protein HrpB7 and the SctK-like protein HrpB4, we used the AlphaFold 2 algorithm and the molecular visualization program UCSF ChimeraX [70, 71]. Superimposition of the structural models of HrcN and HrpB7 onto the EscN_6_-EscO complex from EPEC suggests that a HrcN hexamer is attached to the stalk-like HrpB7 protein [69, 75] (Fig. S1 and Fig. 2A). Furthermore, the AlphaFold 2 model and the assembly of protein structures by ChimeraX predict a hexagonal protein complex, in which the central HrcN hexamer is linked via HrcL to pod-like structures assembled by HrcQ and dimers of HrcQ_C_ (Fig. 2A, Fig. S2 and Fig. S3). A similar hexagonal arrangement was shown by cryo-electron microscopy of T3S system from *Shigella* and *Salmonella* spp [24, 25]. The N- and C-terminal regions of HrcQ are separated by a flexible linker and HrcQ_C_ adopts a saddle-shaped structure as was also predicted for SctQ_C_ proteins from animal pathogens [31, 32]. A 1:2 complex as the minimal unit in pod-like structures of sorting platforms was also reported for SctQ and SctQ_C_ proteins from *S. flexneri* and *Yersinia* [31, 32]. Additionally, a similar arrangement was described for the flagellar C ring components FliM and FliN, which share similarities with SctQ and SctQ_C_ proteins [76].

The N-terminal region of HrcQ likely interacts with the SctK-like protein HrpB4 which connects HrcQ to the cytoplasmic domains of the IM ring component HrcD [37]. Structural modelling predicts the binding sites for HrcQ and the cytoplasmic N-terminal domain of HrcD (HrcD_Nt_) at opposite sides of HrpB4 (Fig. 2A and Fig. S2). This confirms the potential role of HrpB4 as a linker between HrcQ and the IM ring of the T3S system [37]. Interestingly, AlphaFold 2 indicates that HrpB4 interacts with a dimer of HrcD_nt_ (Fig. 2 and Fig. S2). This observation aligns with earlier findings showing that the SctK protein from *Salmonella* spp. interacts with a tetramer of the cytoplasmic domain of SctD, leading to structural rearrangements in the IM ring upon activation of the T3S system [74]. Likewise, the association of six HrpB4 proteins with the IM ring may induce rearrangements in the cytoplasmic domains of HrcD, thus possibly facilitating the attachment of the predicted sorting platform. To characterize the impact of the proposed protein assemblies on T3S and to validate the AlphaFold 2 model of the predicted sorting platform from *Xe*, we investigated the interactions of HrcN, HrcL and HrcQ by genetic approaches, interaction and localization studies as is described below.

### Interactions of the predicted sorting platform components HrcQ, HrcL, HrcN and HrpB4

**Fig. 3:**
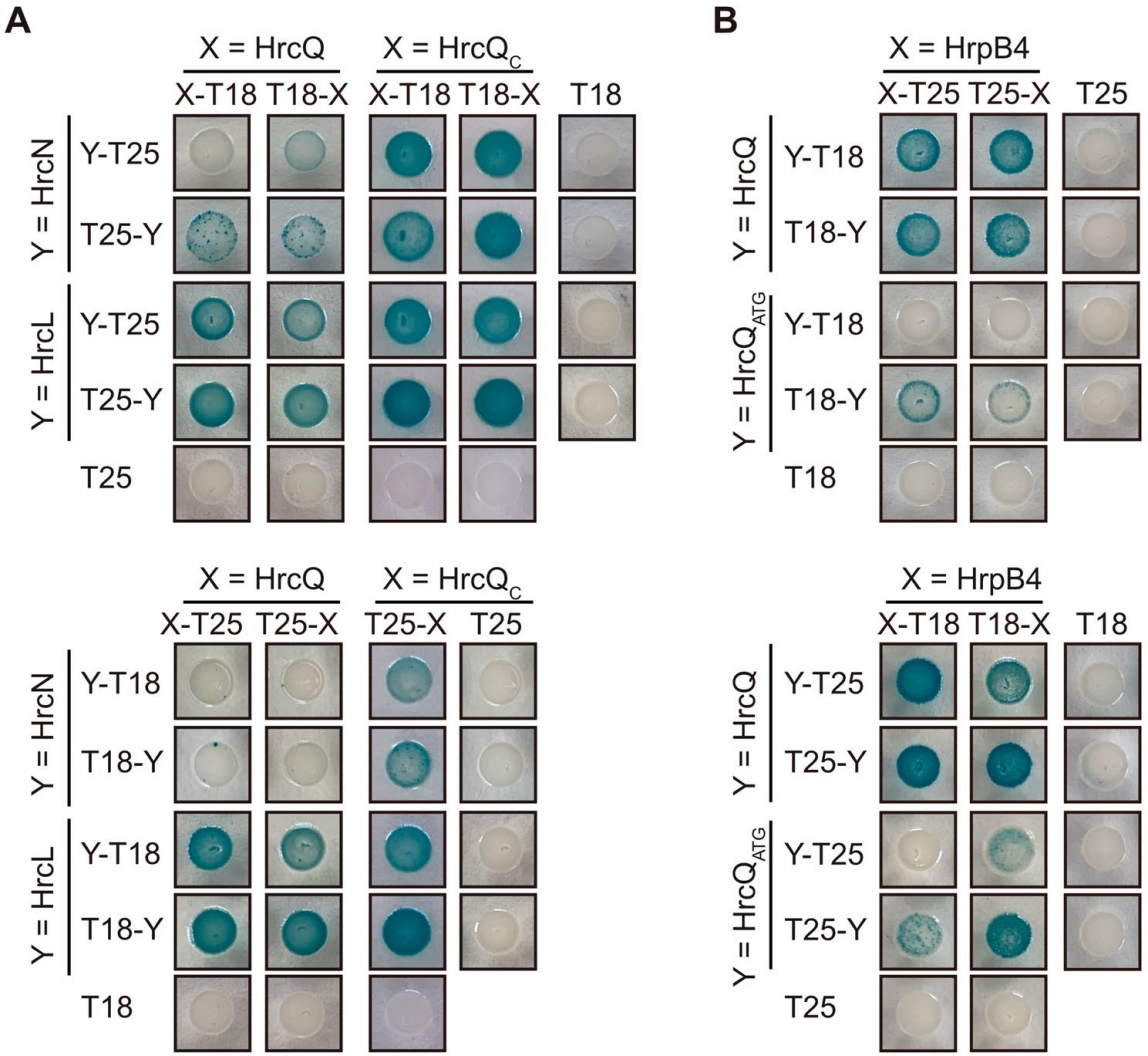
Interaction studies with HrcQ, HrcL, HrcN and HrpB4 using the BACTH system. (**A**) HrcQ and HrcQ_C_ interact with HrcN and HrcL. BACTH assays were performed with T25 and T18 fusions of HrcQ, HrcQ_C_, HrcL and HrcN in *E. coli* DHM1 cells. Bacterial cultures were spotted on indicator plates and representative results for each combination were photographed after 5–7 days. As controls, each fusion protein was tested against the T25 or T18 domain. (**B**) The N-terminal region of HrcQ contributes to the interaction with the linker protein HrpB4. T18 and T25 fusions of HrcQ, HrcQ_ATG_ and HrpB4 as indicated were analysed in DHM1 cells as described in (**A**). HrcQ_ATG_ refers to the annotated HrcQ protein, which lacks the N-terminal 30 amino acids. HrcQ_C_-T25 unspecifically interacts with the T18 domain and was, therefore, not included. All fusion proteins were stably synthesized as was shown by immunoblot analysis [37, 59] (Fig. S5 and S6). Experiments were performed at least three times with independent transformants and similar results. One representative colony is shown

Previous in vitro pull-down studies suggested that HrcD, HrpB4, HrcQ, HrcN and HrcL interact with themselves as well as with each other as is summarized in Fig. S4. To validate these interactions and to experimentally confirm the AlphaFold 2-predicted associations of HrcQ, HrcL, HrcN and HrpB4, we performed additional protein-protein interaction studies using the BACTH system, which is based on the reconstitution of the catalytic domain of the adenylate cyclase (Cya) from two subdomains (T18 and T25) [77, 78]. The interaction of T18 and T25 fusions leads to cAMP synthesis and the expression of the *lac* operon. In *E. coli* reporter strains lacking the native *cya* gene, LacZ activity results in blue colonies on indicator plates containing X-Gal [77, 78]. For BACTH assays, we used expression constructs encoding HrcQ, HrcL, HrcN and HrpB4 as N- or C-terminal fusion partners of T18 and T25 domains. *hrcQ* expression constructs contained the annotated *hrcQ* coding sequence and 30 additional codons with a predicted TTG translational start codon upstream of the annotated GTG start site [59]. We previously reported that translation of *hrcQ* presumably starts at the TTG codon, which is located downstream of a predicted Shine Dalgarno sequence and is required for wild-type HrcQ levels [59]. The annotated HrcQ protein, which was used in previous interaction studies, is hereafter designated “HrcQ_ATG_” and presumably lacks the N-terminal 30 amino acids [59]. BACTH assays revealed that HrcQ weakly interacts with HrcN when analysed as fusion partner of the T18 but not the T25 domain (Fig. 3A). However, both T18 and T25 fusions of HrcQ interacted with HrcL and HrpB4 in all possible combinations, suggesting that HrcQ binds to both HrcL and HrpB4 (Fig. 3A and B). Unlike HrcQ, the annotated HrcQ_ATG_ only weakly interacted with HrpB4, indicating that the extra 30 amino acids at the N-terminus of HrcQ enhance its association with HrpB4 (Fig. 3B). This is also supported by the structural model, which shows that the N-terminal region of HrcQ is closely associated with HrpB4, while the C-terminal region interacts with HrcL (see above, Fig. 2A and C; Fig. S2 and Fig. S3). We also performed interaction studies with HrcQ_C_, which likely acts as a chaperone for HrcQ and binds to both HrcQ and HrpB4 [37, 59] (Fig. S4). Similarly to HrcQ, HrcQ_C_ interacted with HrcN and HrcL, suggesting that the C-terminal region of HrcQ is involved in the association with the ATPase complex (Fig. 3A). All proteins were stably synthesized in *E. coli* as was shown by immunoblot analyses of bacterial cell extracts (Fig. S5 and S6) [37, 59]. Weak protein-protein interactions were, therefore, likely not caused by protein instabilities. Taken together, we conclude from these results that the N-terminal region of HrcQ contributes to the interaction with HrpB4 whereas the C-terminal region is involved in the binding of HrcQ to HrcN and HrcL as predicted by AlphaFold 2.

### The N- and C-terminal regions of HrcL interact with HrcQ and HrcN, respectively

**Fig. 4:**
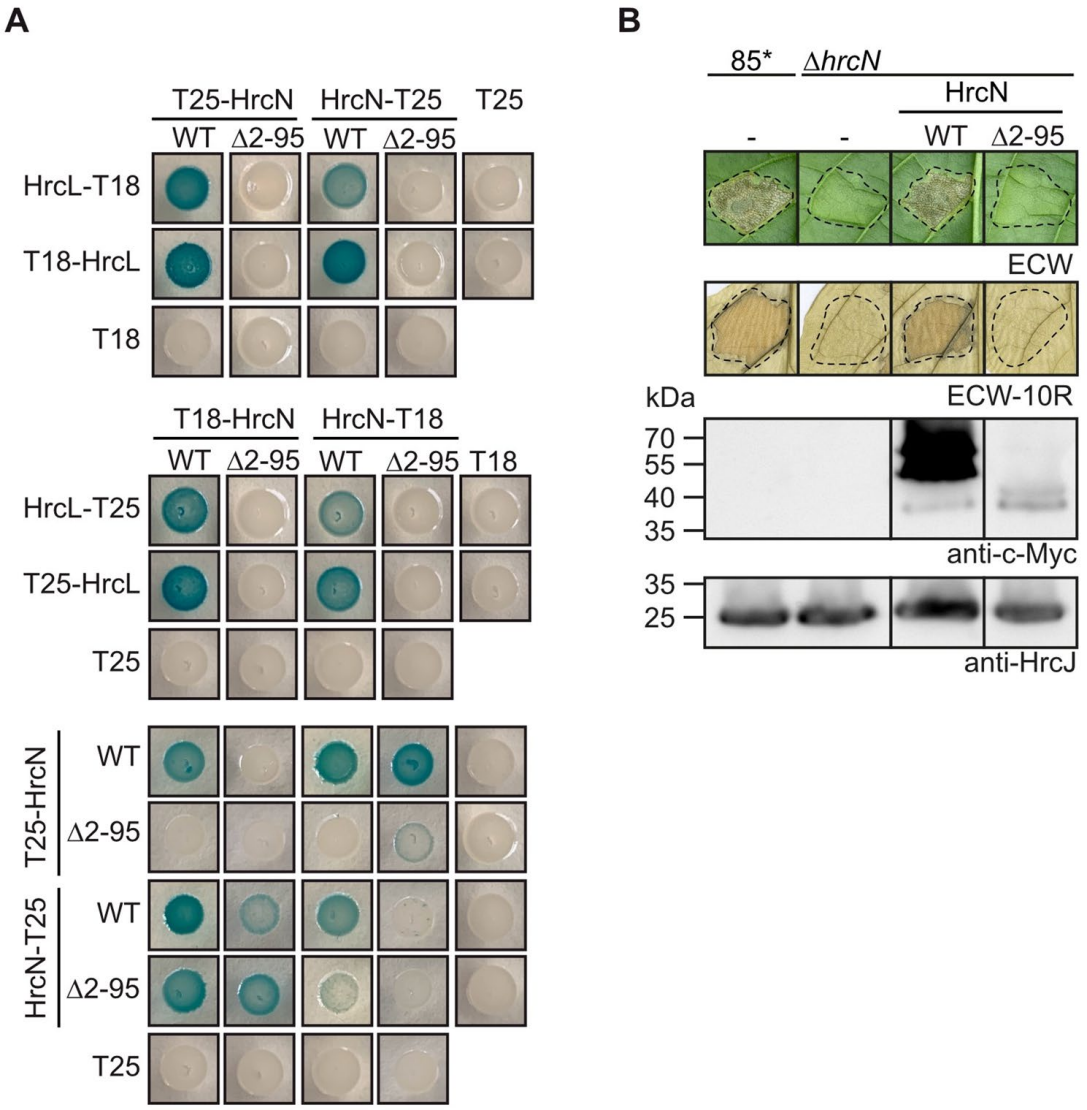
The N-terminal region of HrcN contributes to pathogenicity and to the interaction with HrcL. (**A**) The N-terminal 95 amino acids of HrcN are essential for the interaction with HrcL. T18 and T25 fusions of HrcN (WT), the N-terminal deletion derivative HrcN_∆2−95_ (∆2–95) and HrcL as indicated were analysed in *E. coli* DHM1 cells and bacteria were cultivated on indicator plates. Photographs were taken after 5–7 days. As a negative control, every protein was tested against the T18 and T25 domain. Immunoblot analysis showed that all fusion proteins were stably synthesized (Fig. S5 and S6) [37]. Experiments were performed at least three times with independent transformants and similar results. One representative colony for every combination is shown. (**B**) The N-terminal region of HrcN is essential for protein function. *Xe* strains 85* and 85*∆*hrcN* (∆*hrcN*) with or without (-) expression constructs encoding HrcN (WT) or HrcN_∆2−95_ (∆2–95) as indicated were infiltrated into leaves of susceptible ECW and resistant ECW-10R pepper plants. Disease symptoms were photographed 7 dpi. For the better visualization of the HR, leaves were bleached in ethanol 2 dpi. Dashed lines indicate the infiltrated areas. Experiments were performed three times with similar results. Representative images are shown. For protein studies, bacteria were grown in minimal medium and equal amounts of cell extracts were analysed by immunoblotting, using antibodies specific for the c-Myc epitope and HrcJ. The original blots are shown in Fig. S8

According to the AlphaFold 2 predictions, HrcL forms spoke-like structures in the predicted sorting platform and interacts via its N-terminal region with HrcQ complexes and via the C-terminal region with HrcN (Fig. 2B and C, Fig. S3). Homo- and heterooligomerization of HrcN and HrcL was previously shown by in vitro pull-down assays [58] and was confirmed in the present study by the results of BACTH assays with T18 and T25 fusions of HrcN and HrcL (Fig. S4 and Fig. S5). According to AlphaFold 2 predictions, the N-terminal region of HrcN comprising the first 95 amino acids provides the binding site for HrcL (Fig. 2B and Fig. S3). In agreement with this model, deletion of the N-terminal 95 amino acids of HrcN abolished the detectable interaction with HrcL but not the self-interaction of HrcN_∆2−95_ in BACTH assays (Fig. 4A). To analyse the contribution of the N-terminal region of HrcN to protein function, we performed complementation studies in *Xe hrcN* mutants. For this, expression constructs encoding C-terminally c-Myc epitope-Please correct the line break.tagged derivatives of HrcN and HrcN_∆2−95_ were introduced into the *Xe hrcN* deletion mutant strain 85*∆*hrcN.* Strain 85* (85-10hrpG*) is a derivative of the wild-type strain 85 − 10 and contains HrpG*, a constitutively active version of the key regulator HrpG. HrpG* activates *hrp* gene expression under non-inducing conditions and leads to enhanced plant reactions [79]. For complementation studies, strains 85* and 85*∆*hrcN* with or without HrcN-c-Myc or HrcN_∆2−95_-c-Myc were infiltrated into leaves of susceptible ECW and resistant ECW-10R pepper plants. ECW-10R plants express the *Bs1* resistance gene and activate the HR upon recognition of the T3E AvrBs1 [80]. As expected, strain 85* induced water soaking and the HR in ECW and ECW-10R plants, respectively, whereas no plant reactions were detected after infiltration of strain 85*∆*hrcN* (Fig. 4B) [63]. The *hrcN* mutant phenotype was complemented by HrcN-c-Myc but not by the HrcN deletion derivative, suggesting that the N-terminal protein region is required for HrcN function (Fig. 4B). Similar results were observed with strain 85 − 10∆*hrcN* which contains the wild-type HrpG regulator and does not overexpress the T3S genes (Fig. S7). Notably, immunoblot analyses revealed reduced protein levels of HrcN_∆2−95_-c-Myc when compared with the wild-type protein, suggesting that the N-terminal region contributes to stability of HrcN (Fig. 4B and Fig. S8).

**Fig. 5:**
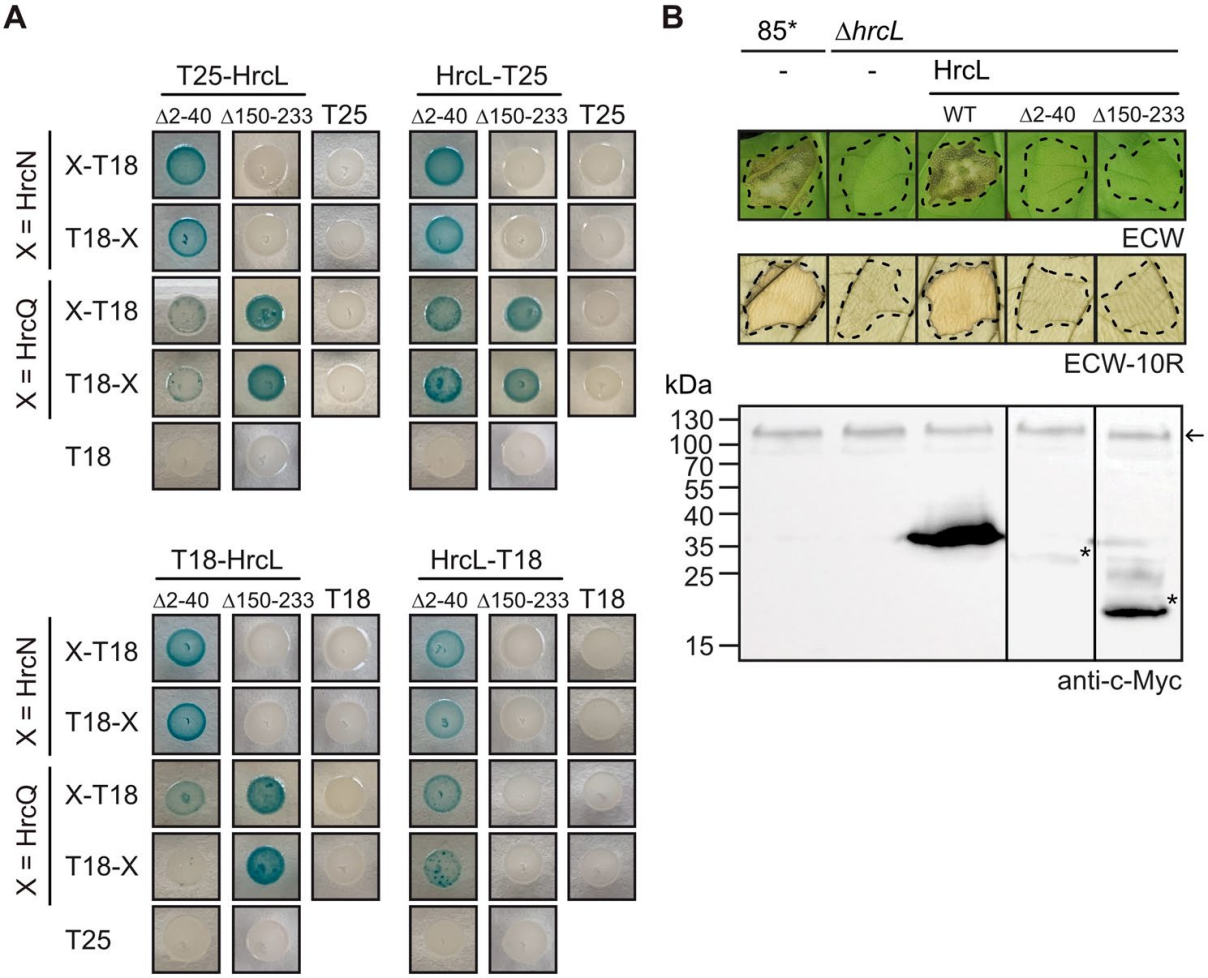
The N- and C-terminal regions of HrcL contribute to the interaction with HrcQ and HrcN, respectively. (**A**) The C-terminal region of HrcL contributes to the interaction with HrcN. Combinations of T18 and T25 fusions of HrcL_∆2−40_ (∆2–40), HrcL_∆150−223_ (∆150–233), HrcN and HrcQ were analysed in *E. coli* DHM1 cells as indicated and bacterial cultures were grown on indicator plates. Photographs were taken after 5–7 days. Experiments were performed at least three times with independent transformants and similar results. One representative colony is shown. All fusion proteins were tested against the T18 and T25 domain alone as indicated and were stably synthesized as was shown by immunoblot analysis (Fig. S5 and S6) [37]. (**B**) Complementation studies with HrcL derivatives. Strains 85* and 85*∆*hrcL* (∆*hrcL*) with or without (-) expression constructs encoding HrcL-c-Myc (WT) or deletion derivatives thereof lacking amino acids 2–40 or 150–233 as indicated were infiltrated into leaves of susceptible ECW and resistant ECW-10R pepper plants as indicated. Dashed lines indicate the infiltrated areas. Disease symptoms were photographed 7 dpi. For the better visualization of the HR, leaves were bleached in ethanol 2 dpi. Experiments were performed three times with similar results, representative images are shown. For the immunological detection of HrcL-c-Myc and derivatives thereof, bacteria were grown in minimal medium and equal amounts of cell extracts were analysed by immunoblotting, using a c-Myc epitope-specific antibody. Signals corresponding to HrcL-c-Myc deletion derivatives are marked by asterisks. The arrow indicates an unspecific signal which indicates equal loading. The original blots are shown in Fig. S9

To investigate the contribution of the N- and C-terminal regions of HrcL to the interaction with HrcN and HrcQ, we performed additional BACTH assays with HrcL derivatives lacking the N-terminal 40 (HrcL_∆2−40_) or the C-terminal 83 amino acids (HrcL_∆150−233_). T18 and T25 fusions of HrcL_∆2−40_ interacted with corresponding T25 and T18 fusions of HrcN whereas the interaction with HrcQ was reduced in some but not all combinations (Fig. 5A). In contrast, deletion of the C-terminal region of HrcL abolished the detectable interaction with HrcN (Fig. 5A). However, HrcL_∆150−233_ interacted with HrcQ in three out of four possible combinations, suggesting that the C-terminal region of HrcL is dispensable for the binding of HrcQ as was predicted by structural modelling (Figs. 2A and 5A). Immunoblot analyses confirmed that all fusion proteins were stably synthesized (Fig. S5 and S6). When analysed by in vivo complementation studies, HrcL deletion derivatives did not restore the wild-type phenotype in strains 85*∆*hrcL* and 85 − 10∆*hrcL*, suggesting that the N- and C-terminal protein regions are essential for HrcL function (Fig. 5B and Fig. S7). Lack of complementation was presumably not caused by a possible dominant-negative effect of HrcL and HrcN deletion derivatives on pathogenicity because ectopic expression of the corresponding genes in strain 85 − 10 did not alter the wild-type phenotype (Fig. S7). Notably, as observed for HrcN, deletion of the N-terminal region led to reduced HrcL levels (Fig. 5B and Fig. S9). It is possible that the N-terminal regions of HrcN and HrcL contribute to protein folding and thus stability. Alternatively, they might be required for stabilization of HrcN and HrcL in the context of the sorting platform. Taken together, we assume that the N- and C-terminal regions of HrcL contribute to pathogenicity and the interaction with HrcQ and HrcN, respectively, as predicted by AlphaFold 2.

### HrcL contributes to HrcQ complex formation

**Fig. 6:**
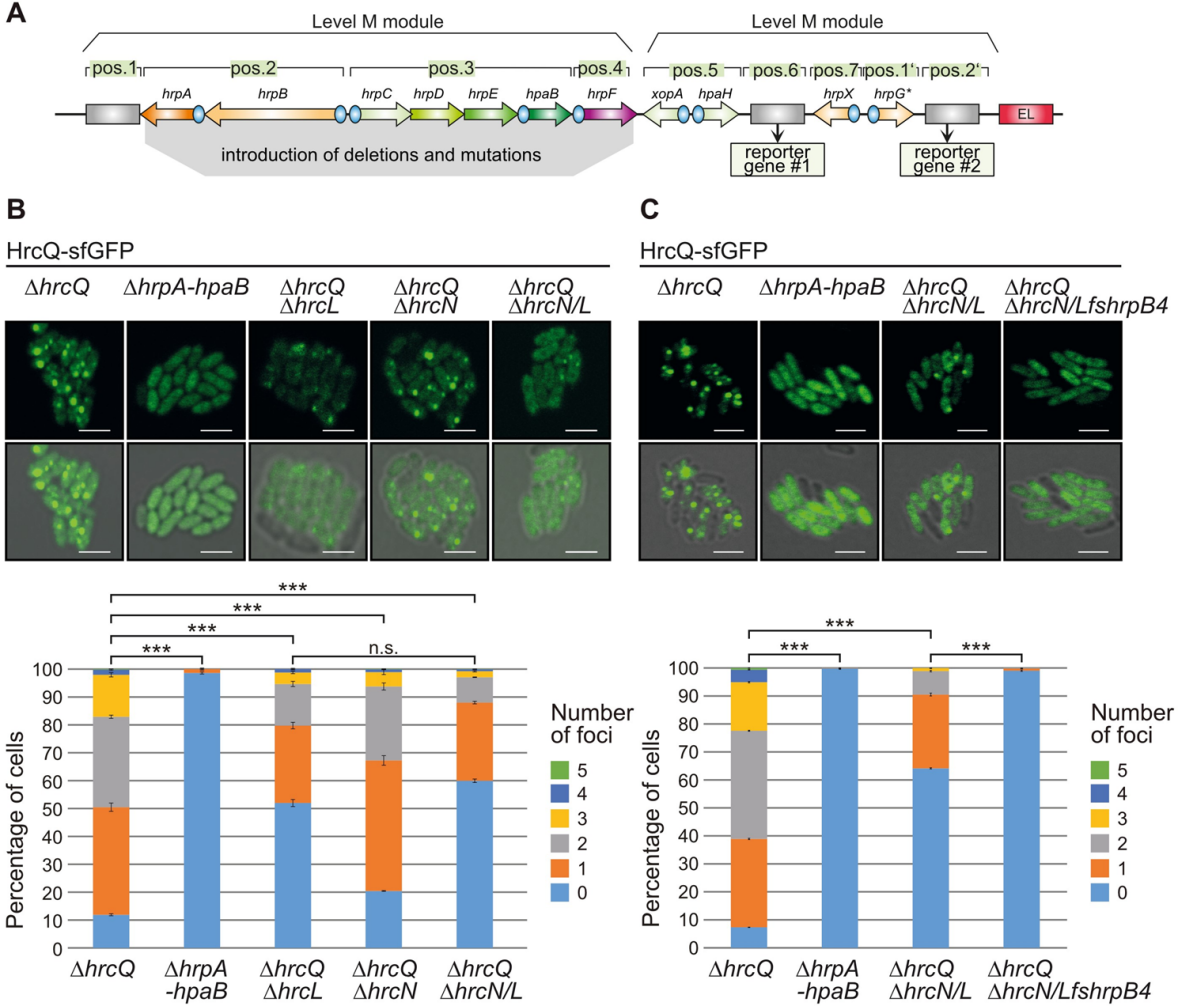
HrcL contributes to fluorescent foci formation by HrcQ-sfGFP. (**A**) Overview of modular level P T3S gene cluster constructs encoding HrcQ-sfGFP. Operons are represented by arrows and promoters by blue circles. Grey rectangles indicate the position of dummy modules, which were replaced by reporter genes. Promoters and genes were assembled by stepwise modular Golden Gate cloning and level P constructs result from the final assembly of two level M modules. EL, end-linker. (**B**) Fluorescent foci formation by HrcQ-sfGFP is compromised in the absence of HrcL. Derivatives of *Xe* strain 85*∆*hrp_*fs*HAGX* containing modular level P constructs with deletions in *hrcQ*,* hrpA-hpaB* operons, *hrcL* and/or *hrcN* and encoding HrcQ-sfGFP as indicated were incubated under T3S-permissive conditions and HrcQ-sfGFP foci formation was analysed by fluorescence microscopy. One representative image for each strain is shown. The scale bar corresponds to 2 μm. The pictures in the lower panel result from an overlay of fluorescent signals with the images of the brightfield channel. The diagrams represent the percentage of cells with zero to five fluorescent foci. For this, fluorescent foci were counted in approximately 500–1000 cells per strain from three different transconjugants. Three asterisks indicate a significant difference between the number of foci in different strains with a *p* value of < 0.001 based on the results of χ^2^ test (*** mean *p* value < 0.001; n, s, not significant). Fig. S12 exemplifies the foci with the weakest fluorescence which were included in the quantification. (**C**) HrcQ-sfGFP foci formation is abolished in the absence of HrcN, HrcL and HrpB4. HrcQ-sfGFP foci formation was analysed as described in (**B**). Foci formation was compared in strains lacking *hrcQ*,* hrcN* and *hrcL* (∆*hrcQ*∆*hrcN/hrcL*) or containing an additional frameshift mutation in *hrpB4* (fs*hrpB4*) as indicated. HrcQ-sfGFP was stably synthesized in all strains as was shown by immunoblot analyses of bacterial cell extracts (Fig. S11 and S13)

We previously described that a HrcQ-sfGFP fusion forms complexes which likely represent the formation of the predicted sorting platform and were visualized by fluorescence microscopy. For these studies, we used *Xe* strains with a modular T3S gene cluster which was assembled from single promoter and gene modules and contains all necessary structural, regulatory and accessory genes [61] (Fig. 6A and Fig. S10). The modular design facilitates genetic approaches for functional studies as well as the insertion of reporter fusions by Golden Gate-based modular cloning [61]. The modular T3S gene cluster is functional in *Xe* strain 85*∆*hrp_*fs*HAGX*, which lacks the native T3S gene cluster and contains frameshift mutations in the regulatory and accessory genes *hpaH*,* xopA*,* hrpG* and *hrpX* [61]. *hpaH* and *xopA* encode a predicted lytic transglycosylase and a putative translocon protein, respectively, and thus likely contribute to the assembly of the T3S system and to T3E delivery [81, 82]. HrpG and HrpX are the key regulators, which activate T3S gene expression when the bacteria enter the plant apoplast or are cultivated in specific minimal media [47, 48, 79]. As reported previously, HrcQ-sfGFP restored pathogenicity when encoded by the modular T3S gene cluster lacking the native *hrcQ* gene, suggesting that the sfGFP fusion partner did not interfere with HrcQ function [61] (Fig. S11). When bacteria were incubated under T3S-permissive conditions at pH 5.3 in minimal medium and analysed by fluorescence microscopy, HrcQ-sfGFP formed one to five fluorescent foci at the cell periphery, suggesting that it assembles in complexes in the presence of a functional T3S system [61] (Fig. 6B and Fig. S12). Foci formation was specifically caused by HrcQ, as sfGFP without fusion partner localized in the bacterial cytoplasm and did not form fluorescent foci (Fig. S12).

**Fig. 7:**
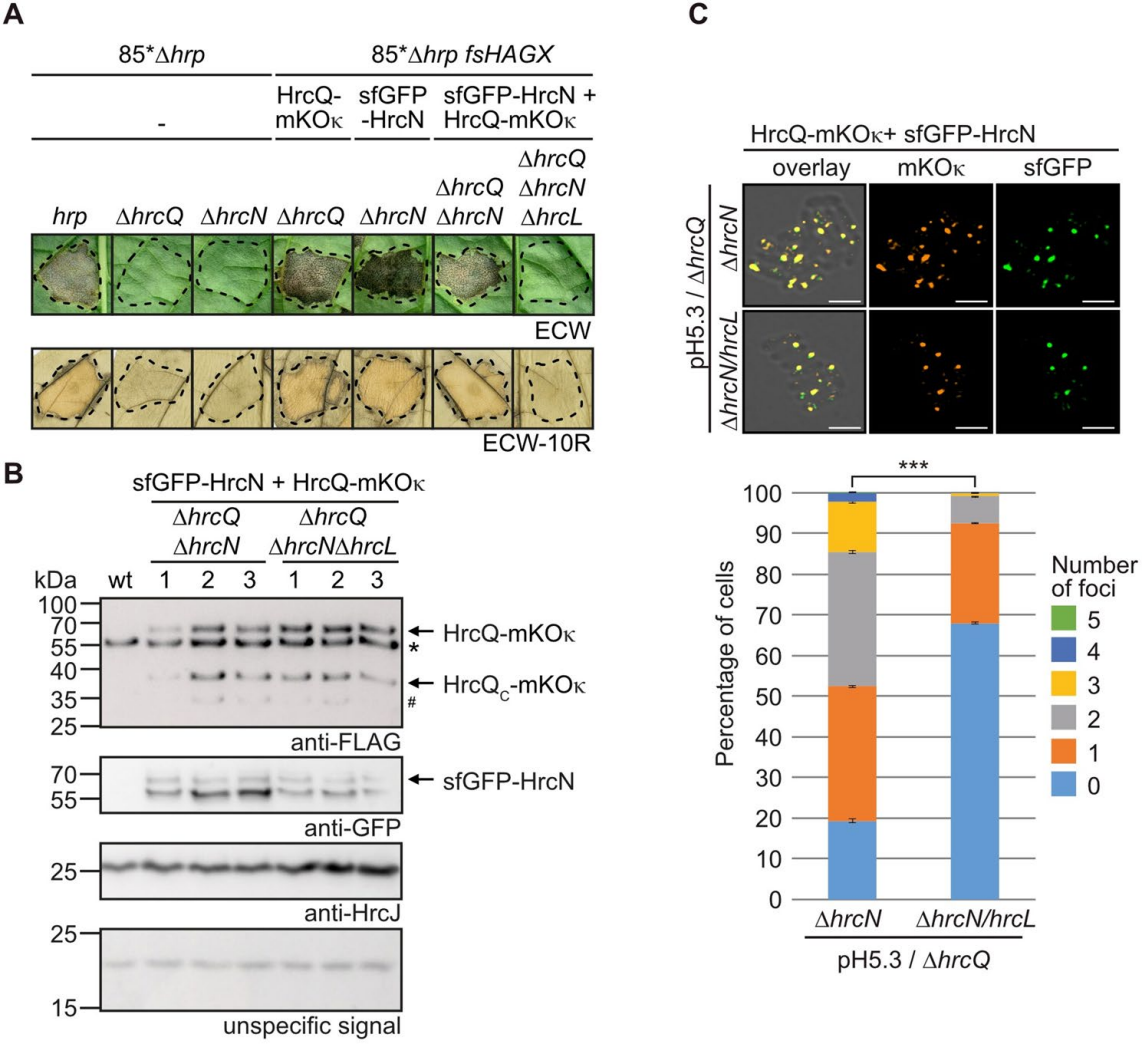
HrcN and HrcQ colocalize and depend on HrcL for efficient complex formation. (**A**) HrcQ-mKO_κ_ and sfGFP-HrcN are functional in *Xe.* For complementation studies, strain 85*∆*hrp* containing the modular T3S gene cluster (*hrp*) with or without deletions in *hrcQ* (∆*hrcQ*) or *hrcN* (∆*hrcN*) and strain 85*∆*hrp_*fs*HAGX* containing modular level P *hrp-HAGX* constructs with deletions in *hrcQ*,* hrcN* and *hrcL* (∆*hrcQ*, ∆*hrcN*,* ∆hrcQ*∆*hrcN* or ∆*hrcQ*∆*hrcN*∆*hrcL*) and encoding the fluorescent fusions proteins HrcQ-mKO_κ_, sfGFP-HrcN or both as indicated were infiltrated into leaves of susceptible ECW and resistant ECW-10R pepper plants. Dashed lines indicate the infiltrated areas. Disease symptoms were photographed 10 dpi. For the better visualization of the HR, leaves were bleached in ethanol 2 dpi. (**B**) Immunological detection of HrcQ-mKO_κ_ and sfGFP-HrcN in *Xe* cell extracts. Strain 85*∆*hrp_*fs*HAGX* containing the wild-type modular T3S gene cluster (wt) and derivatives thereof with deletions in *hrcQ*,* hrcN* and *hrcL* (*∆hrcQ*∆*hrcN* or ∆*hrcQ*∆*hrcN*∆*hrcL*) and encoding HrcQ-mKO_κ_ and sfGFP-HrcN as indicated were incubated in T3S-inducing minimal medium. Equal amounts of cell extracts from three transconjugants (labeled as 1, 2 and 3) of each strain were analysed by immunoblotting using antibodies specific for GFP, HrcJ or the FLAG epitope, which is present in the HrcQ-mKO_κ_ fusion, as indicated. An unspecific signal detected by the FLAG epitope-specific antibody demonstrates equal loading and is marked with an asterisk. The original blots are shown in Fig. S21. (**C**) Fluorescence microscopy analysis of HrcQ-mKO_κ_ and sfGFP-HrcN. *Xe* strains containing modular T3S gene cluster constructs as described in (**B**) were incubated in T3S-permissive minimal medium at pH 5.3 without antibiotics and foci formation was analysed by fluorescence microscopy. One representative image per strain is shown. Cells without fluorescent signals likely lost the expression construct during overnight incubation. The scale bar corresponds to 2 μm. The pictures in the left panel result from an overlay of the fluorescent signals with the images of the brightfield channel. To enhance or sharpen fine structures and details of sfGFP-HrcN, the images were processed using the Leica LIGTHNING detection concept. The diagram shows the number of foci in bacterial cells which were counted in approximately 500–1000 cells per strain from three different transconjugants. Significant differences between the number of foci with a *p* value of < 0.001 based on the results of a χ^2^ test are indicated by three asterisks. Experiments were performed three times with similar results. Fig. S12 exemplifies the foci with the weakest fluorescence which were included in the quantification

We previously showed that HrcQ-sfGFP complex formation depends on the IM ring component HrcD [37, 59]. In the present study, we analysed the contribution of HrcL and HrcN to HrcQ-sfGFP foci formation. For this, the corresponding genes were deleted individually or in combination from the modular T3S gene cluster (Fig. S10). Infection studies with derivatives of strain 85*∆*hrp_*fs*HAGX* containing the respective modular T3S gene cluster constructs revealed that deletion of *hrcL* or *hrcN* led to a loss of pathogenicity as expected (Fig. 7A and Fig. S11) [58, 61]. Complementation studies confirmed that the *hrcN* mutant phenotype is specifically caused by the absence of HrcN and not by a polar effect of the deletion on downstream genes [58, 61] (Fig. 7A). Similarly, the *hrcL* mutant phenotype was complemented by ectopic expression of *hrcL* (Fig. S11). When bacteria were analysed by fluorescence microscopy, deletion of *hrcL* or *hrcN* led to reduced HrcQ-sfGFP foci (Fig. 6B). The reduced foci formation observed in the *hrcL* mutant remained almost unaffected by the additional deletion of *hrcN*, indicating that HrcN and HrcL do not have an additive effect on HrcQ complex assembly (Fig. 6B). In the absence of HrcL, HrcN and HrpB4, HrcQ-sfGFP foci formation was nearly abolished (Fig. 6B and C). Differences in foci formation were not caused by alterations in protein stabilities because HrcQ-sfGFP was stably synthesized in all strains (Fig. S11 and Fig. S13). Our findings support the previous observation that HrpB4 acts as a linker between HrcQ and the IM ring component HrcD [37]. Additionally, we conclude that HrpB4 is essential for HrcQ docking to the T3S system in the absence of HrcL and HrcN.

### HrcQ and HrcN colocalize at the T3S system

To investigate a possible association of HrcN with HrcQ complexes, we performed colocalization studies. For this, HrcQ and HrcN were analysed as fusion partners of the fluorescent reporter proteins mKOκ and sfGFP, respectively [83, 84]. The corresponding gene fusions were inserted individually or in combination into the flanking region of modular T3S gene clusters deleted in the native *hrcQ* and/or *hrcN* genes and the resulting expression constructs were transferred into strain 85*∆*hrp_*fs*HAGX.* When bacteria were infiltrated into susceptible ECW and resistant ECW-10R pepper plants, HrcQ-mKO_κ_ and sfGFP-HrcN complemented the *hrcQ* and *hrcN* mutant phenotypes with respect to disease symptoms and HR induction, respectively, suggesting that both proteins were functional (Fig. 7A). Similarly, simultaneous expression of *hrcQ-mKO*_*κ*_ and *sfgfp-hrcN* restored pathogenicity of a *hrcQ/hrcN* double deletion mutant (Fig. 7A). Both proteins were stably synthesized as was shown by immunoblot analyses of bacterial cell extracts (Fig. 7B). Fluorescence microscopy revealed that HrcQ-mKO_κ_ and sfGFP-HrcN assembled in fluorescent foci, which colocalized in the presence of an active T3S system, suggesting that HrcQ and HrcN are part of a common protein complex (Fig. 7C and Fig. S12). Foci formation by HrcQ-mKO_κ_ was specifically attributed to HrcQ as a fusion between mKO_κ_ and the type III effector protein XopB localized to the cytoplasm (Fig. S14). Lack of HrcL led to a significant reduction in the number of HrcQ/HrcN-containing foci (Fig. 7C). This indicates that HrcL promotes complex formation by both HrcQ (see above, Fig. 6) and HrcN which is in agreement with the predicted architecture of the sorting platform and the role of HrcL as linker protein. For additional colocalization studies, we generated fluorescent fusions of HrpB4, HrcL, the stalk-like protein HrpB7, the IM ring components HrcJ and HrcD as well as the OM secretin HrcC. Proteins were analysed in fusion with different flexible linkers and fluorescent reporters including sfGFP, mCherry, mKOκ, TagBFP and sfTq2oc as is summarized in Fig. S15 and Table S3. Localization studies, however, were not possible because the fusion proteins were either unstable or the fluorescent reporters interfered with protein function as was shown by complementation studies (data not shown).

### Stability of the ATPase HrcN depends on HrcL and HrcQ

**Fig. 8:**
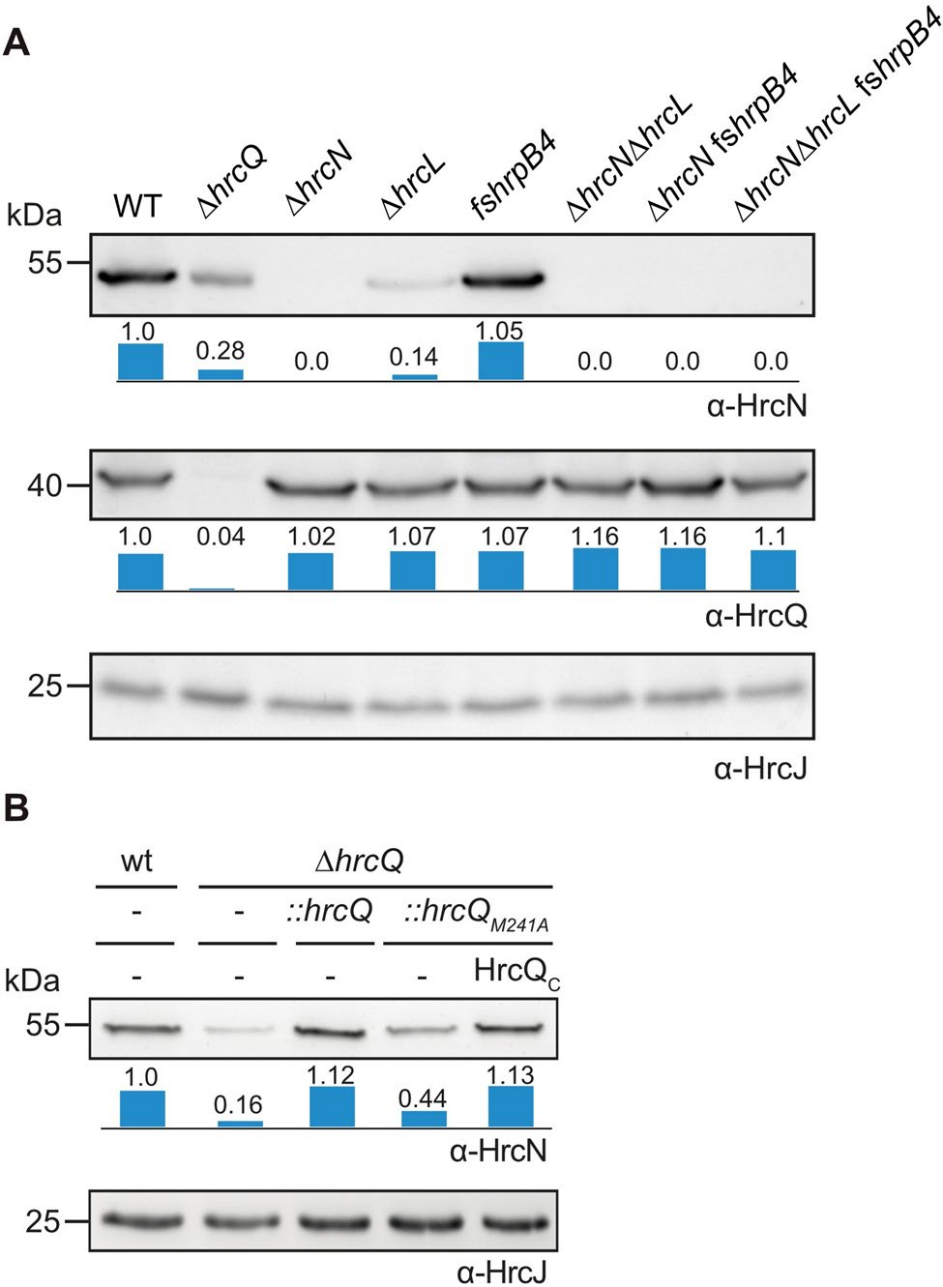
The ATPase is stabilized by HrcQ and HrcL. (**A**) HrcL and HrcQ contribute to the stability of the ATPase HrcN. Strain 85*∆*hrp* containing the wild-type modular T3S gene cluster (wt) or derivatives thereof with deletions in *hrcQ*,* hrcN*,* hrcL* or a frameshift mutation (fs) in *hrpB4* as indicated were grown in minimal medium. Equal amounts of cell extracts from bacteria in the exponential growth phase were analysed by immunoblotting using antibodies specific for HrcN, HrcQ and HrcJ, respectively. Detection of the IM ring protein HrcJ served as loading control. The amounts of HrcN and HrcQ were compared to HrcJ levels and quantified by immunoblot signal intensity measurements as described in Material and Methods. Signal intensities in the wild-type strain were set to 1. The original blots are shown in Fig. S16. (**B**) The alternative translation initiation product of HrcQ, HrcQ_C_, contributes to HrcN stability. The contribution of HrcQ_C_ to HrcN stability was analysed in strains 85* (wt) and 85*∆*hrcQ* (∆*hrcQ*) with or without (-) chromosomal insertion of *hrcQ* or *hrcQ*_*M241A*_ as indicated. For complementation studies, an expression construct encoding HrcQ_C_ under control of the *lac* promoter was introduced into strain 85*∆*hrcQ::hrcQ*_*M241A*_ as indicated. Equal amounts of cell extracts were analysed by immunoblotting using HrcN- and HrcJ-specific antibodies. Signal intensities were analysed as described in (**A**). Experiments were performed three times with similar results. One representative immunoblot is shown. The original blots are shown in Fig. S17

Considering that HrcL plays a role in the assembly of HrcQ and HrcN complexes, we wondered whether it also promotes the stability of both proteins. Consistent with our earlier discovery that HrcL stabilizes HrcN in *Xe* [58], we observed reduced levels of sfGFP-HrcN in a *hrcL* mutant (Fig. 7B). To investigate whether HrcN stability depends on additional sorting platform components, we performed immunoblot analyses of cell extracts from strain 85*∆*hrp* with modular T3S gene cluster constructs lacking *hrcQ*,* hrcN*,* hrcL* or *hrpB4.* HrcN was stably synthesized in the presence of the wild-type T3S gene cluster and in the *hrpB4* mutant whereas significantly reduced levels of HrcN were detected in the absence of *hrcQ* or *hrcL* (Fig. 8A and Fig. S16). In contrast to HrcN, the levels of HrcQ were unaffected in the analysed deletion mutants. The detection of equal amounts of the IM ring component HrcJ in all samples confirmed equal loading (Fig. 8A and Fig. S16).

To further investigate the contribution of HrcQ to HrcN stability, we analysed HrcN levels in the *hrcQ* deletion mutant strain 85*∆*hrcQ*, in which *hrcQ* or a derivative thereof containing a mutation in the internal translation start site (M241A mutation) were reinserted *in cis* into the flanking region of the T3S gene cluster [59]. Wild-type levels of HrcN were detected upon *in cis* expression of *hrcQ* but not of *hrcQ*_*M241A*_ in the *hrcQ* deletion mutant, suggesting that the alternative translation product HrcQ_C_ contributes to HrcN stability (Fig. 8B and Fig. S17). *In trans* expression of *hrcQ*_*C*_ in the *hrcQ*_*M241A*_ mutant restored wild-type levels of HrcN, indicating that reduced HrcN stability in strain 85*∆*hrcQ::hrcQ*_*M241A*_ was specifically caused by the absence of HrcQ_C_ (Fig. 8B and Fig. S17). Our findings suggest that the stability of the ATPase HrcN depends on HrcL, HrcQ and HrcQ_C_, and thus possibly on the association of HrcN with a HrcL-HrcQ/HrcQ_C_ complex. In agreement with this hypothesis, deletion of the N-terminal region of HrcN, which provides the binding site for HrcL, led to reduced HrcN stability (see above, Fig. 4B).

### The association of HrcQ and HrcN with the T3S system depends on the external pH

**Fig. 9:**
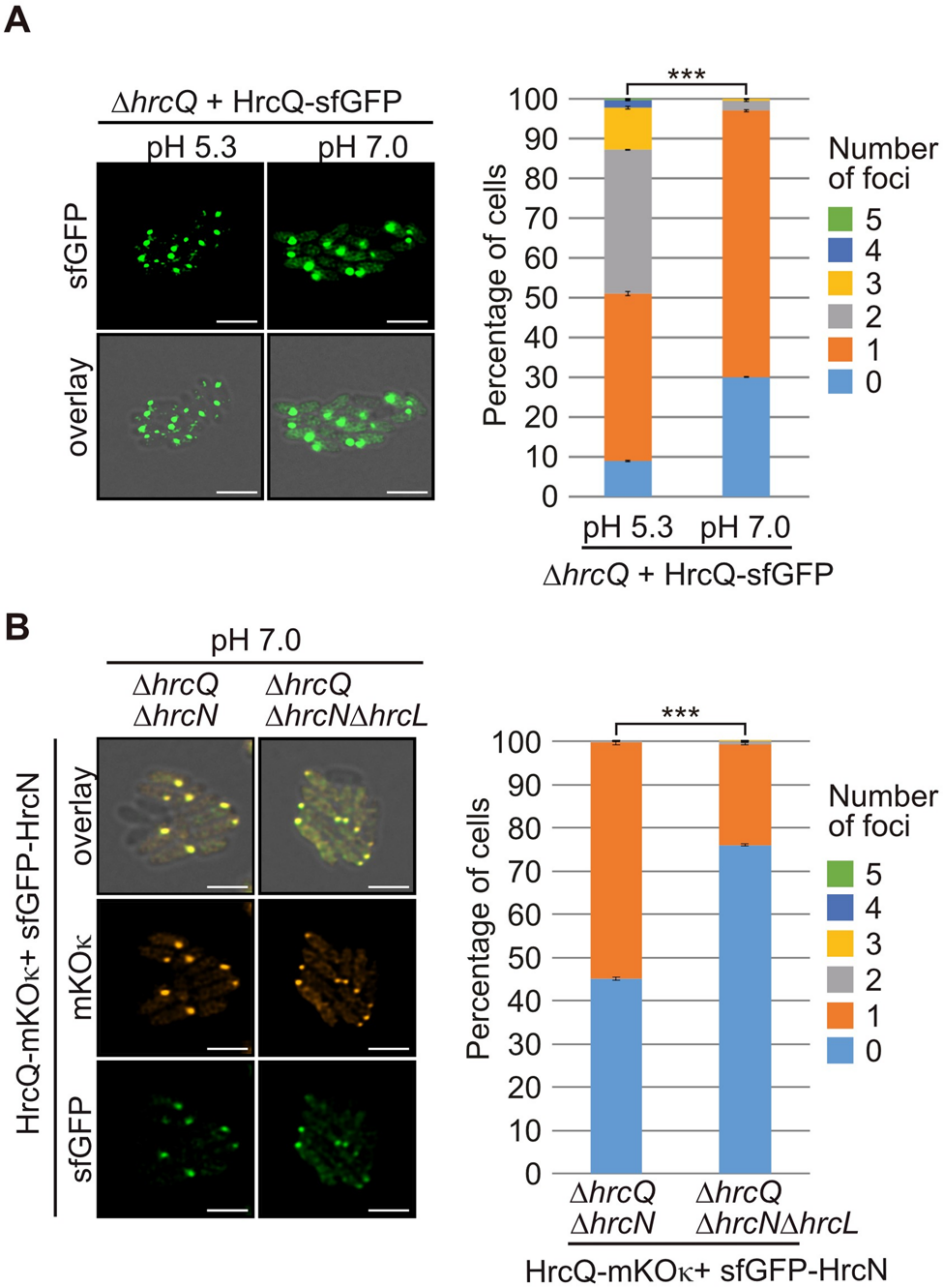
Localization of HrcQ and HrcN at the cell periphery depends on the external pH. (**A**) HrcQ-sfGFP assembles in cytoplasmic complexes when bacteria are incubated at pH 7.0. Strain 85*∆*hrp_*fs*HAGX* containing modular level P *hrp-HAGX* constructs with a deletion in *hrcQ* and encoding HrcQ-sfGFP were incubated in minimal medium at pH 5.3 and pH 7.0 without antibiotics. Foci formation was monitored by fluorescence microscopy. Cells without fluorescent signals likely lost the expression construct during overnight incubation. One representative image for each strain is shown. The scale bar corresponds to 2 μm. The pictures in the lower panel show the overlay of the fluorescent signals with the images of the brightfield channel. The diagram summarizes foci formation in approximately 500–1000 cells per strain at pH 5.3 and pH 7.0. Significant differences between the number of foci with a *p* value of < 0.001 based on the results of a χ^2^ test are indicated by three asteriscs. Fig. S12 exemplifies the foci with the weakest fluorescence which were included in the quantification. (**B**) HrcL contributes to foci formation of HrcQ and HrcN in the bacterial cytoplasm at pH 7.0. Strain 85*∆*hrp_*fs*HAGX* containing modular level P *hrp-HAGX* constructs deleted in *hrcQ*,* hrcN* or *hrcL* as indicated and encoding HrcQ-mKO_κ_ and sfGFP-HrcN were incubated in minimal medium at pH 7.0. Fluorescent foci formation was monitored as described in (A). To enhance or sharpen fine structures and details of sfGFP-HrcN, the images were processed using the Leica LIGTHNING detection concept

The sorting platform from animal-pathogenic bacteria was previously described as dynamic structure which changes its localization from the bacterial cytoplasm to the T3S system and thus possibly promotes substrate loading into the secretion channel [27, 34, 35]. To investigate a similar dynamic localization of HrcN-HrcL-HrcQ complexes in *Xe*, we analysed HrcQ-sfGFP complex formation in bacteria which were incubated in minimal medium at pH 5.3 or pH 7.0. T3S in *Xe* is activated in minimal medium at pH 5.3, leading to the detection of secreted proteins in bacterial culture supernatants whereas T3S is abolished in minimal medium at pH 7.0 [62]. As shown previously, HrcQ-sfGFP formed one to five fluorescent foci when bacteria were incubated at pH 5.3 under T3S-permissive conditions [61] (Fig. 9A). In contrast, at pH 7.0, only one bright fluorescent spot was detected which likely reflects a cytoplasmic HrcQ-sfGFP complex (Fig. 9A). The cytoplasmic localization of HrcQ-sfGFP complexes at pH 7.0 remained unchanged in the absence of the IM ring component HrcD, suggesting that it was not caused by the docking of HrcQ to the T3S system (Fig. S18 and Fig. S19). As reported previously, the cytoplasmic domain of HrcD is required for the localization of HrcQ complexes at the T3S system at pH 5.3 [59].

**Fig. 10:**
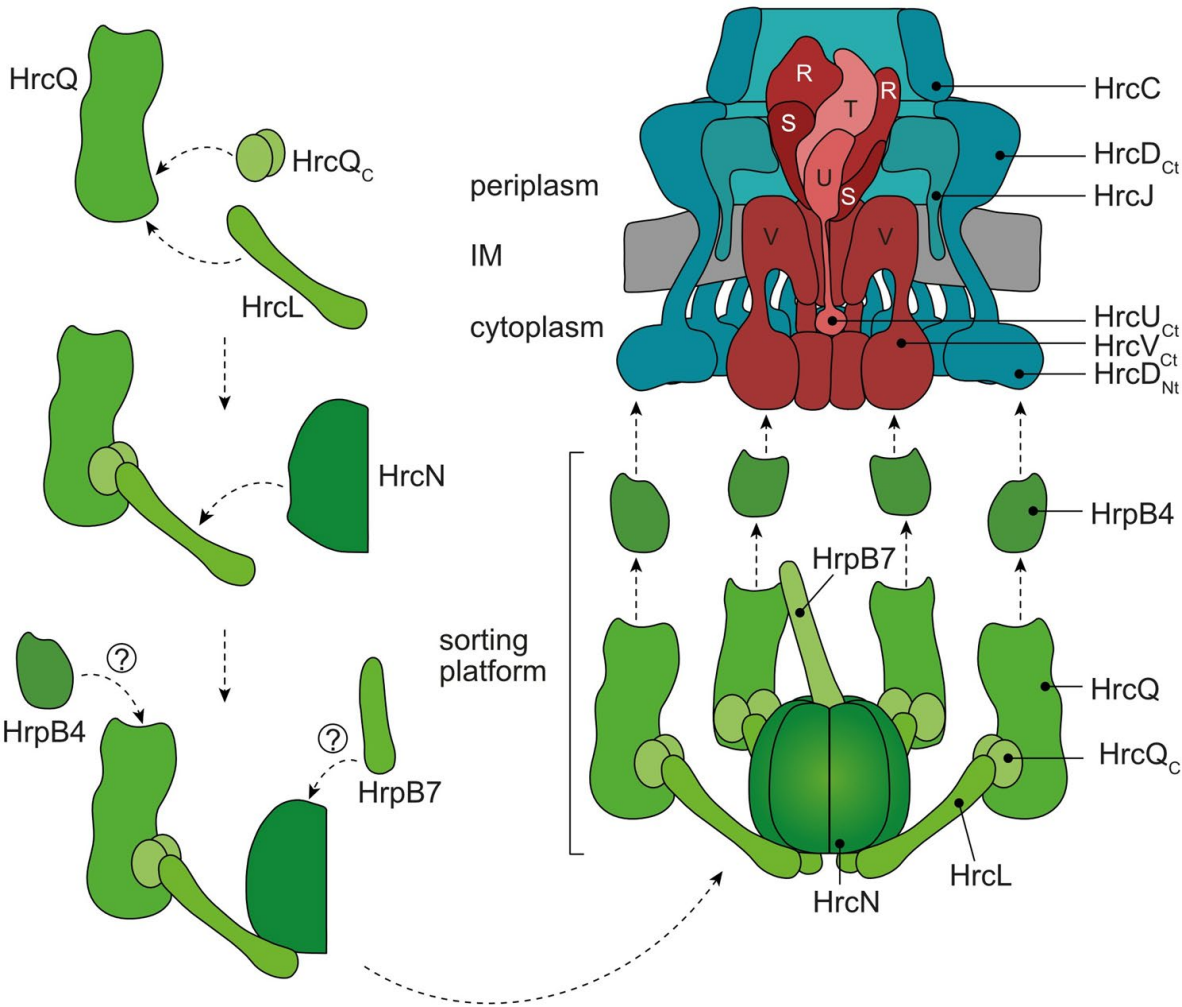
Model of the predicted attachment of the sorting platform to the T3S system from *Xe*. A complex of HrcQ, a HrcQ_C_ dimer and HrcL likely assembles in the bacterial cytoplasm and allows the subsequent association of the ATPase HrcN to the C-terminal region of HrcL. HrcN forms a hexamer which associates with the SctO-like protein HrpB7. The predicted HrcN-HrcL-HrcQ complex binds via HrpB4 to the cytoplasmic domain of the IM ring component HrcD, preferentially under T3S-permissive conditions at a low external pH (see text for details)

To investigate whether cytoplasmic HrcQ-sfGFP complexes represent the predicted assembled sorting platform, we performed colocalization studies with HrcQ-mKO_κ_ and sfGFP-HrcN fusions encoded by corresponding modular expression constructs as described above (Fig. 7C and Fig. S10). Both HrcQ-mKO_κ_ and sfGFP-HrcN were detected in one bright fluorescent spot when bacteria were incubated under T3S non-permissive conditions at pH 7.0 (Fig. 9B and Fig. S20). This suggests that HrcQ and HrcN integrate into the same cytoplasmic protein complex, which presumably corresponds to the sorting platform and detaches from the T3S system under T3S non-permissive conditions. Additional deletion of *hrcL* in the modular T3S gene cluster reduced the number of bacteria with one fluorescent spot (Fig. 9B and Fig. S20). This supports the above observation that HrcL stabilizes HrcQ and HrcN complexes. Taken together, we conclude that HrcN, HrcL and HrcQ assemble in a common dynamic protein complex which presumably corresponds to the sorting platform and changes its localization from the cytoplasm to the T3S system in response to the external pH (Fig. 10).

## Discussion

The existence, architecture and assembly of the sorting platform in plant-pathogenic bacteria has long been enigmatic because the low copy number of the T3S system made its isolation and in situ visualization challenging. In the present study, we identified an interaction network of the predicted sorting platform components HrcN, HrcL and HrcQ from *Xe* and showed that they dynamically associate with the T3S system in response to the external pH level. According to structural modelling, HrcN, HrcL and HrcQ form a hexagonal cage-like complex which resembles the sorting platform from animal pathogens and consists of the central hexameric ATPase HrcN which is linked via HrcL to six peripheral HrcQ/HrcQ_C_ complexes. The C-terminal region of HrcN faces the IM and interacts with HrpB7, a small SctO-like protein which forms highly stable complexes. HrpB7 presumably connects the ATPase with the cytoplasmic domain of the export apparatus component HrcV as was reported for homologous Sct proteins from animal-pathogenic bacteria [6, 75, 85]. Structure predictions, in vivo interaction assays and complementation studies with HrcN and HrcL deletion derivatives indicate that the N-terminal region of HrcN interacts with the C-terminal region of HrcL. This interaction presumably maintains HrcN function and stability. HrcL likely forms spoke-like structures between HrcN and HrcQ as is suggested by structural modelling and our interaction studies. Given that HrcL can interact with itself, it might be present as a homo-dimer in the HrcN-HrcL-HrcQ complex as was shown for SctL proteins from animal-pathogenic bacteria [24, 25, 86, 87].

We previously studied the assembly of the predicted sorting platform in *Xe* by the analysis of a HrcQ-sfGFP fusion protein using fluorescence microscopy and a modular T3S gene cluster [59, 61]. Prerequisite for localization studies with fluorescent reporter fusions is their stability and functionality in *Xe*. To date, HrcQ and HrcN are the only suitable candidates for localization studies in *Xe* because other T3S system components were either unstable or not functional when analysed as fusion partners of fluorescent reporters. As shown previously, efficient foci formation by HrcQ-sfGFP depends on its C-terminal alternative translation product, HrcQ_C_, which interacts with HrcQ and likely acts as a chaperone [59]. The finding that HrcQ_C_ also associates with the ATPase complex, the linker protein HrpB4 and the IM ring protein HrcD suggests that it contributes to the assembly and the docking of the predicted sorting platform to the T3S system [59]. We assume that the broad binding specificity of HrcQ_C_ is due to a SPOA (surface presentation of antigen) domain which is also present in SctQ_C_ proteins from animal-pathogenic bacteria and is likely involved in protein-protein interactions [33, 59, 60, 88]. Structural modelling suggests that HrcQ_C_ dimerizes and associates with HrcQ in pod-like structures at the periphery of the predicted sorting platform. Interestingly, the HrcQ/HrcQ_C_ complex shares structural similarities with corresponding SctQ/SctQ_C_ complexes from animal pathogens and the corresponding FliM-FliN_3_ complex of the flagellar C ring [32, 76].

According to structure predictions and interaction studies, the C-terminal region of HrcQ associates with HrcL whereas its N-terminal region binds to the SctK-like protein HrpB4. We previously reported that HrpB4 connects the HrcQ/HrcQ_C_ pod-like structures to the cytoplasmic domain of the IM ring component HrcD and thus acts similarly to SctK proteins from animal-pathogenic bacteria [37]. Notably, deletion of *hrpB4* significantly reduced but did not completely abolish foci formation by HrcQ-sfGFP, suggesting that HrcQ attaches to the IM rings of the T3S system in the absence of HrpB4 [37]. Lack of the IM ring component HrcD or its cytoplasmic domain, however, leads to the formation of cytoplasmic HrcQ-sfGFP complexes as expected [37]. HrcQ stability and complex formation in the cytoplasm is independent of HrpB4, indicating that HrpB4 is dispensable for the assembly of the predicted sorting platform in the cytoplasm [37] (Fig. 10). It is yet unknown whether HrpB4 detaches together with the HrcQ-HrcL-HrcN complex from the membranes or whether its association with HrcQ depends on HrcD as was reported for SctQ and SctK proteins from *Salmonella* spp [74]. Future studies in *Xe* will, therefore, focus on the analysis of interactions between HrcQ, HrpB4 and HrcD in different mutant backgrounds using in vivo photocrosslinking approaches.

Our fluorescence microscopy studies showed that the efficient docking of HrcQ complexes to the T3S system requires HrcL which connects HrcQ to the ATPase HrcN. This is reminiscent of the finding that complex formation between SctQ and SctL in *Salmonella* spp. is a prerequisite for the interaction of SctQ with the linker protein SctK. It was, therefore, proposed that binding of SctL induces a conformational change in SctQ which promotes the SctQ-SctK interaction [74]. In *S. flexneri*, however, SctL is dispensable for the incorporation of the SctQ and SctK proteins Spa33 and MxiK into pod-like structures [89], suggesting a different assembly of the sorting platform. In *Xe*, the formation of HrcQ-HrcL complexes presumably precedes the attachment of the ATPase HrcN which plays a minor role in the formation of fluorescent HrcQ-sfGFP foci (Fig. 10). Given that HrcQ, HrcQ_C_ and HrcL are required to maintain wild-type HrcN levels, binding to the HrcQ-HrcL complex likely stabilizes HrcN. Similar findings were reported for the ATPases from *Salmonella* spp. and *S. flexneri* which depend on the assembly of the sorting platform for oligomerization and stabilization [24, 25, 27, 33, 74]. In *Yersinia* spp., however, the ATPase YscN stabilizes YscQ complexes and promotes interactions of sorting platform components which presumably form a variety of dynamic cytoplasmic complexes [34, 36, 60, 86]. Fluorescence recovery after photobleaching measurements and single molecule tracking microscopy showed a dynamic localization of YscQ between the T3S system and the cytoplasm with increased exchange rates under secretion-permissive conditions, suggesting a continuous release and attachment of the sorting platform to the T3S system [34, 35, 90]. A similar dynamic localization of the sorting platform was reported in *Salmonella* spp. and was proposed to shuttle T3S substrates to the secretion apparatus [20, 27, 60, 91]. In *Yersinia* spp. and *S. flexneri*, sorting platform components dissociate from the T3S system at a low external pH and thus likely suppress T3S when the bacteria pass the stomach [39]. Interestingly, we also observed a dynamic localization of the predicted sorting platform from *Xe* in response to changes in the external pH level. Thus, under T3S-permissive conditions at pH 5.3, HrcQ and HrcN colocalize in fluorescent foci at the cell periphery in the presence of a functional T3S system [57, 59, 61]. Under T3S non-permissive conditions at pH 7.0, however, both HrcQ and HrcN were present in a common complex in the bacterial cytoplasm, suggesting that the change in pH leads to the detachment of the entire HrcQ/HrcQ_C_-HrcL-HrcN complex from the membranes. This is in line with our previous observation that HrcQ predominantly localizes to the cytoplasm at pH 7.0 whereas an acidic pH promotes its membrane association [57].

The dynamic attachment of the sorting platform to the T3S system in response to environmental conditions such as changes in the external pH possibly increases the local concentration of T3S cargo proteins and thus activates T3S [34, 39]. Given that the sorting platform associates with T3S substrates, it might provide a reservoir for secreted proteins in the cytoplasm [91]. A recent study in *Yersinia* spp. revealed a direct interaction of T3Es with SctQ and SctL in the bacterial cytoplasm and suggests that the binding of one T3E to one SctQ protein and the exchange rates of SctQ between the cytoplasm and the T3S system accounts for the delivery rate of T3Es [92]. In *Xe*, potential docking sites for T3S substrates in the predicted sorting platform are provided by HrcQ and HrcN [57, 58]. We assume that the acidic pH in the plant apoplast, possibly together with an additional yet unknown plant-derived signal, is required for T3S and thus leads to the attachment of the predicted sorting platform to the T3S system [62, 93, 94]. The mechanisms underlying pH sensing, recognition of T3S substrates and the activation of the T3S system are not yet understood. In *Yersinia* spp. and *S. flexneri*, the external pH is presumably sensed by the IM ring component SctD, which dissociates from the T3S system at an acidic pH [39]. Similarly, a rearrangement of SctD proteins from a 24mer oligomeric transmembrane ring to tetrameric complexes was described in *Salmonella* spp. and occurs upon docking of the sorting platform [25]. In *Xe*, a potential role of HrcD in the pH-mediated docking of the predicted sorting platform remains to be investigated. Furthermore, future in vivo approaches in *Xe* including localization studies and photocrosslinking are required for an in-depth analysis of the mechanisms leading to the recruitment of the HrcN-HrcL-HrcQ complex to the T3S system, the binding of cargo proteins and the activation of T3S. Unravelling the interplay between T3S substrates and structural components of the T3S system will elucidate the mechanisms underlying T3E delivery and might also help to develop strategies for the management of bacterial diseases in crop plants.

## Conclusions

Taken together, our study provides the first detailed analysis of a predicted sorting platform from a plant pathogen. Structural modelling suggests that HrcQ, HrcL and HrcN form a hexagonal cage-like structure, similar to sorting platforms found in animal pathogens, which is crucial for the function of the T3S system. As revealed by fluorescence microscopy studies, the predicted sorting platform dynamically associates with the T3S system in response to environmental stimuli and thus presumably functions as reservoir for T3S substrates in the cytoplasm. The proposed dynamics in protein-protein interactions shed light on T3S regulation in plant-pathogenic bacteria, which is still poorly understood. Our findings draw parallels between the sorting platforms of plant and animal pathogens, suggesting that similar mechanisms may be employed across different bacterial species.

## Electronic supplementary material

Below is the link to the electronic supplementary material.

Supplementary Material 1

## Data Availability

Data generated or analysed during this study are included within the article and its additional files. The Xe proteins with the following accession numbers were analysed: HrcD (CAJ22050), HrpB4 (AAB08459), HrcQ (AAD21320), HrcN (CAJ22063), HrcL (CAJ22062) and HrpB7 (AAB08462). The datasets underlying the analysis of fluorescent foci formation, which are summarized in this article, are available from the corresponding author on reasonable request.
