# Supplementary material for: The predicted sorting platform dynamically associates with the type III secretion system from Xanthomonas euvesicatoria in response to the external pH
Source: BMC Microbiol. 2025 Aug 25;25:545. doi: 10.1186/s12866-025-04227-6 (PMC12376730; doi:10.1186/s12866-025-04227-6)
Supplement: Supplementary file 1 — Supplementary Material 1 [file 12866_2025_4227_MOESM1_ESM.pdf]

Supplementary material

The predicted sorting platform of *Xanthomonas euvesicatoria* dynamically associates with the type III secretion system in response to the external pH

Christian Otten and Daniela Büttner*

Contents:

Tables:

Table S1 Bacterial strains and plasmids used in this study.

Table S2 Primers used in this study.

Table S3 Summary of studies with fluorescent fusions of T3S system components from *Xe.*

Figures:

Figure S1 Superimposition of the ATPase complexes from EPEC and *Xe.*
Figure S2 Structural models of interacting sorting platform and IM ring components
Figure S3 Structural modeling of HrcN-HrcL and HrcL-HrcQ complexes.
Figure S4 Overview of interactions between predicted sorting platform components of *Xe.*
Figure S5 Protein analysis and interaction studies with T18 and T25 fusions of HrcQ, HrcL and HrcN.
Figure S6 Original blots for Figure S5.
Figure S7 Complementation studies with N- and C-terminal deletion derivatives of HrcL and HrcN.
Figure S8 Original blots for Figure 4B.
Figure S9 Original blots for Figure 5B.
Figure S10 Schematic overview of modular T3S gene cluster constructs used in this study.
Figure S11 Infection and protein studies with *Xe* strains containing modular T3S gene cluster constructs.
Figure S12 Fluorescence microscopy imaging of HrcQ-sfGFP and GFP in *Xe*.
Figure S13 Original blots for Figure S11.
Figure S14 Localization studies with XopB-mKO$_{\kappa}$.
Figure S15 Overview of fluorescent reporter fusions analysed for this study.
Figure S16 Original blots for Figure 8A.
Figure S17 Original blots for Figure 8B.

Figure S18 Analysis of HrcQ-sfGFP complexes at pH 5.3 and pH 7.0.

Figure S19 Original blots for Figure S18.

Figure S20 HrcQ-mKO$_{\kappa}$ and sfGFP-HrcN are stably synthesized at pH 7.0.

Figure S21 Original blots for Figure S20**.**

Table S1 Bacterial strains and plasmids used in this study.

Strain or plasmid	Relevant characteristics[1]	Reference(s)
**Strain**		
Xe		
85-10	Pepper-race 2; wild type; Rif^R	Canteros, 1990; Kousik and Ritchie, 1998
85*	85-10 derivative containing the *hrpG** mutation	Wengelnik et al., 1999
85-10Δ*hrcN*	85-10 derivative deleted in codons 13 - 432 of *hrcN*	Lorenz and Büttner, 2009
85*Δ*hrcN*	Derivative of strain 85-10Δ*hrcN* containing *hrpG**	Lorenz and Büttner, 2009
85-10Δ*hrcL*	85-10 derivative deleted in codons 108 - 172 of *hrcL*	Rossier et al., 2000
85*Δ*hrcL*	Derivative of strain 85-10Δ*hrcL* containing *hrpG**	Rossier et al., 2000
85*Δ*hrcQ*	85* derivative deleted in codons 11 - 243 of *hrcQ* followed by a nonsense mutation	Lorenz et al., 2012
85*Δ*hrcQ::hrcQ*	Derivative of strain 85*Δ*hrcQ* carrying *hrcQ-c-myc* under control of the native promoter inserted into the *hpaFG* region	Lorenz et al., 2012
85*Δ*hrcQ::*$hrcQ_{M241A}$	Derivative of strain 85*Δ*hrcQ* carrying $hrcQ_{M241A}$-*c-myc* under control of the native promoter inserted into the *hpaFG* region	Otten et al., 2021b
85*Δ*hrp*	Derivative of strain 85* with a deletion of 21424 bp including the *hrpB* to *hrpF* operons as well as the promoter and the first 214 codons of *hrcC*	Hausner et al., 2019
85*Δ*hrp*_fs*HAGX*	Derivative of strain 85*Δ*hrp* with frameshift mutations after codons 26 of *hrpX*, 12 of *hrpG*, 16 of *xopA* and 7 of *hpaH*	Hausner et al., 2019
E.coli		
OneShot®TOP10	F^-, *mcrAΔ(mrr-hsdRMS-mcrBC)*, *Φ80lacZΔM15*, Δ*lacX74*, *recA1*, *araΔ139Δ(ara-leu)7697*, *galU*, *galK*, *rpsL*, *endA1*, *nupG*	Invitrogen
JM109	F^-, *traD36* $proA^+B^+$ $lacI^q$ Δ*(lacZ)M15*/ Δ*(lac-proAB) glnV44* $e14^-$ *gyrA96 recA1 relA1 endA1 thi hsdR17*	Yanisch-Perron et al., 1985
DHM1	F-, *cya-854*, *recA1*, *endA1*, *gyrA96* (Nal^R), *thi1*, *hsdR17*, *spoT1*, *rfbD1*, *glnV44*(AS)	Karimova et al., 2005
**Plasmids**		
pICH41021	Derivative of pUC19 with mutated *Bsa*I site; Ap^R	Gift from S. Marillonnet
pBRM-P	Derivative of pBRM containing a *lacZα* fragment flanked by *Bsa*I recognition sites and a 3 x c-Myc epitope-encoding sequence lacking the *lac* promoter upstream of the 5' *Bsa*I site, Gm^R	Szczesny et al., 2010
pB-PhrcL	Derivative of pBRM-P encoding HrcL-c-Myc under control of the native *hrpB* operon promoter	This study
pB-$PhrcL_{\Delta 2\text{-}40}$	Derivative of pBRM-P encoding $HrcL_{\Delta 2\text{-}40}$-c-Myc under control of the native *hrpB* operon promoter	This study
pB-$PhrcL_{\Delta 150\text{-}233}$	Derivative of pBRM-P encoding $HrcL_{\Delta 150\text{-}233}$-c-Myc under control of the native *hrpB* operon promoter	This study
pB-PhrcN	Derivative of pBRM-P encoding HrcN-c-Myc under control of the native *hrpB* operon promoter	This study

pB-PhrcN$_{\Delta 2\text{-}95}$	Derivative of pBRM-P encoding HrcN$_{\Delta 2\text{-}95}$-c-Myc under control of the native *hrpB* operon promoter	This study
pBhrcQ$_C$	Derivative of pBRM encoding HrcQ$_C$-c-Myc under control of the *lac* promoter	Otten et al., 2021
pBsfGFP	Derivative of pBRM encoding sfGFP under control of the *lac* promoter	This study
**BACTH vectors**		
pUT18	BACTH vector, derivative of pUC19, encodes the T18 fragment (amino acids 225 – 399) of CyaA downstream of the *lac* promoter and a multiple cloning site for classical cloning, Ap^R	Euromedex; Karimova et al., 2001
pUT18C	BACTH vector, derivative of pUT18, encodes the T18 fragment (amino acids 225 – 399) of CyaA downstream of a *lac* promoter; the multiple cloning site for classical cloning is inserted at the 3' end of the T18-encoding fragment, Ap^R	Euromedex; Karimova et al., 2001
pUT18$_{GG}$	Golden Gate-compatible derivative of pUT18 containing *lacP-eforRed* flanked by *Bsa*I sites upstream of the FLAG-T18 fragment; Gm^R	Otten and Büttner, 2021a
pUT18C$_{GG}$	Golden Gate-compatible derivative of pUT18C containing *lacP-eforRed* flanked by *Bsa*I sites downstream of the T18-FLAG fragment; Gm^R	Otten and Büttner, 2021a
pKT25	BACTH vector, derivative of low copy number plasmid pSU40, encodes the first 224 amino acids of the T25 fragment of CyaA downstream of the *lac* promoter, the multiple cloning site for classical cloning is inserted at the 3' end of the T25 encoding fragment, Km^R	Euromedex; Karimova et al., 2001
pKNT25	BACTH vector, derivative of pKT25, contains the T25-encoding fragment downstream of a multiple cloning site for classical cloning and the *lac* promoter, Km^R	Euromedex; Karimova et al., 2001
pKT25$_{GG}$	Golden Gate-compatible derivative of pKT25 encoding the T25 fragment downstream of the *lac* promoter and in frame with a C-terminal FLAG epitope-encoding sequence; contains *lacP-eforRed* flanked by *Bsa*I sites downstream of the *T25-FLAG* fragment; Km^R	Otten and Büttner, 2021a
pKNT25$_{GG}$	Golden Gate-compatible derivative of pKNT25, encodes the T25 fragment in frame with an N-terminal FLAG epitope-encoding sequence downstream of the *lac* promoter, contains *lacP-eforRed* flanked by *Bsa*I sites upstream of the *T25-FLAG* fragment; Km^R	Otten and Büttner, 2021a
pUT18$_{GG}$-hrcN	Derivative of pUT18$_{GG}$ encoding HrcN-FLAG-T18	Otten and Büttner, 2021a
pUT18C$_{GG}$-hrcN	Derivative of pUT18C$_{GG}$ encoding T18-FLAG-HrcN	Otten and Büttner, 2021a
pKT25$_{GG}$-hrcN	Derivative of pKT25$_{GG}$ encoding T25-FLAG-HrcN	Otten and Büttner, 2021a
pKNT25$_{GG}$-hrcN	Derivative of pKNT25$_{GG}$ encoding HrcN-FLAG-T25	Otten and Büttner, 2021a
pUT18$_{GG}$-hrcN$_{\Delta 2\text{-}95}$	Derivative of pUT18$_{GG}$ encoding HrcN$_{\Delta 2\text{-}95}$-FLAG-T18	This study
pUT18C$_{GG}$-hrcN$_{\Delta 2\text{-}95}$	Derivative of pUT18C$_{GG}$ encoding T18-FLAG-HrcN$_{\Delta 2\text{-}95}$	This study
pKT25$_{GG}$-hrcN$_{\Delta 2\text{-}95}$	Derivative of pKT25$_{GG}$ encoding T25-FLAG-HrcN$_{\Delta 2\text{-}95}$	This study

pKNT25GG-hrcN$_{\Delta2-95}$	Derivative of pKNT25GG encoding HrcN$_{\Delta2-95}$-FLAG-T25	This study
pUT18GG-hrcL	Derivative of pUT18GG encoding HrcL-FLAG-T18	Otten and Büttner, 2021a
pUT18CGG-hrcL	Derivative of pUT18CGG encoding T18-FLAG-HrcL	Otten and Büttner, 2021a
pKT25GG-hrcL	Derivative of pKT25GG encoding T25-FLAG-HrcL	Otten and Büttner, 2021a
pKNT25GG-hrcL	Derivative of pKTN25GG encoding HrcL-FLAG-T25	Otten and Büttner, 2021a
pUT18GG-hrcL$_{\Delta2-40}$	Derivative of pUT18GG encoding HrcL$_{\Delta2-40}$-FLAG-T18	This study
pUT18CGG-hrcL$_{\Delta2-40}$	Derivative of pUT18CGG encoding T18-FLAG-HrcL$_{\Delta2-40}$	This study
pKT25GG-hrcL$_{\Delta2-40}$	Derivative of pKT25GG encoding T25-FLAG-HrcL$_{\Delta2-40}$	This study
pKNT25GG-hrcL$_{\Delta2-40}$	Derivative of pKNT25GG encoding HrcL$_{\Delta2-40}$-FLAG-T25	This study
pUT18GG-hrcL$_{\Delta150-233}$	Derivative of pUT18GG encoding HrcL$_{\Delta150-233}$-FLAG-T18	This study
pUT18CGG-hrcL$_{\Delta150-233}$	Derivative of pUT18CGG encoding T18-FLAG-HrcL$_{\Delta150-233}$	This study
pKT25GG-hrcL$_{\Delta150-233}$	Derivative of pKT25GG encoding T25-FLAG-HrcL$_{\Delta150-233}$	This study
pKNT25GG-hrcL$_{\Delta150-233}$	Derivative of pKNT25GG encoding HrcL$_{\Delta150-233}$-FLAG-T25	This study
pUT18GG-hrcQ	Derivative of pUT18GG encoding HrcQ-FLAG-T18	This study
pUT18CGG-hrcQ	Derivative of pUT18CGG encoding T18-FLAG-HrcQ	This study
pKT25GG-hrcQ	Derivative of pKT25GG encoding T25-FLAG-HrcQ	This study
pKNT25GG-hrcQ	Derivative of pKNT25GG encoding HrcQ-FLAG-T25	This study
pUT18GG-hrcQATG	Derivative of pUT18GG encoding HrcQATG-FLAG-T18	This study
pUT18CGG-hrcQATG	Derivative of pUT18CGG encoding T18-FLAG-HrcQATG	This study
pKT25GG-hrcQATG	Derivative of pKT25GG encoding T25-FLAG-HrcQATG	This study
pKNT25GG-hrcQATG	Derivative of pKNT25GG encoding HrcQATG-FLAG-T25	This study
pUT18GG-hrpB4	Derivative of pUT18GG encoding HrpB4-FLAG-T18	Otten and Büttner, 2021a
pUT18CGG-hrpB4	Derivative of pUT18CGG encoding T18-FLAG-HrpB4	Otten and Büttner, 2021a
pKT25GG-hrpB4	Derivative of pKT25GG encoding T25-FLAG-HrpB4	Otten and Büttner, 2021a
pKNT25GG-hrpB4	Derivative of pKNT25GG encoding HrpB4-FLAG-T25	Otten and Büttner, 2021a
**Constructs for modular cloning**		
Destination vectors		
pAGM9121	pUC19-derived vector, *lacZα* fragment flanked by *Bpi*I sites; Sm^R	Addgene #51833; Weber et al., 2011
pAGM1311	pUC19-derived level -1 vector, *lacZα* fragment flanked by *Bsa*I sites; Km^R	Addgene #47983; Weber et al., 2011
pICH41276	pUC19-derived level 0 vector, *lacZα* fragment flanked by *Bpi*I sites; Sm^R	Addgene #47994; Weber et al., 2011
pICH41308	pUC19-derived level 0 vector, *lacZα* fragment flanked by *Bpi*I sites; Sm^R	Addgene #47998; Weber et al., 2011

pICH41331	pUC19-derived level 0 vector, *lacZα* fragment flanked by *Bpi*I sites; Sm^R	Addgene #47999; Weber et al., 2011
pICH47742	Level 1 destination vector derived from pBIN19 and pUC19, *lacZα* flanked by *Bsa*I sites, for level M position 2 (forward); Ap^R	Addgene #48001; Weber et al., 2011
pICH47781	Level 1 destination vector derived from pBIN19 and pUC19, *lacZα* fragment flanked by *Bsa*I sites, for level M position 6 (forward); Ap^R	Addgene #48005; Weber et al., 2011
pICH47811	Level 1 destination vector derived from pBIN19 and pUC19, *lacZα* flanked by *Bsa*I sites, for level M position 2 (reverse); Ap^R	Addgene #48008; Weber et al., 2011
pAGM8031	Level M vector derived from pBIN19 and pUC19, *lacZα* fragment flanked by *Bpi*I sites; Sm^R	Addgene #48037; Weber et al., 2011
pAGM8079	Level M vector derived from pBIN19 and pUC19, *lacZα* fragment flanked by *Bpi*I sites; Sm^R	Addgene #48041; Weber et al., 2011
pICH75322	Level P vector derived from pPZP200 and pUC19, *lacZα* fragment flanked by *Bsa*I sites, ColE1 and pVS1 ori, Km^R	Addgene #48051; Weber et al., 2011
End-linker constructs		
pICH50881	Derived from pUC19, level M end linker for position 3; ACTA/- *Bsa*I and ACTA/ GGGA *Bpi*I fusion sites; Ap^R,	Addgene #48045; Weber et al., 2011
pICH50900	Derived from pUC19, level M end linker for position 5; CAGA/ - *Bsa*I and CAGA/ GGGA *Bpi*I fusion sites; Ap^R	Addgene #48047; Weber et al., 2011
pICH79264	Derived from pUC19, level P end linker for position 3; ACTA / GGGA *Bsa*I and ACTA/- *Bpi*I fusion sites; Ap^R	Addgene #48059; Weber et al., 2011
Dummy modules		
pICH54011	Derived from pBIN19 and pUC19, 15-bp insert for level M position 1 with TGCC/ GCAA *Bpi*I fusion sites; Ap^R	Addgene #48065; Weber et al., 2011
pICH54022	Derived from pBIN19 and pUC19, 15-bp insert for level M position 2’ with GCAA/ ACTA *Bpi*I fusion sites; Ap^R	Addgene #48066; Weber et al., 2011
Level -2 constructs		
pAGB192	Level -2 construct; derivative of pAGM9121 containing *hrcL*; Sm^R	Hausner et al., 2019
pAGB193	Level -2 construct; derivative of pAGM9121 containing *hrcN*; Sm^R	Hausner et al., 2019
pAGB194	Level -2 construct; derivative of pAGM9121 containing *hrpB7*; Sm^R	Hausner et al., 2019
pAGB195	Level -2 construct; derivative of pAGM9121 containing *hrcT*; Sm^R	Hausner et al., 2019
pAGB196	Level -2 construct; derivative of pAGM9121 containing *hrcC* downstream of the native *hrpA* operon promoter; Sm^R	Hausner et al., 2019
pAGB514	Level -2 construct; derivative of pAGM9121 containing *hrcN* deleted in bp 102 – 1257; Sm^R	This study
pAGB608	Level -2 construct; derivative of pICH41021 containing *hrcN* including the stop codon and lacking the start codon for generation of N-terminal fusions; Ap^R	This study
pAGB659	Level -2 construct; derivative of pAGM9121 containing *hrcN/L* deletion module (deletion of entire *hrcL* and first 1283 bp of *hrcN*); Sm^R	This study
pAGB957	Level -2 construct; derivative of pAGM9121 containing *hrcL* deleted in bp 169 – 597; Sm^R	This study
pAGB1118	Level -2 construct; derivative of pAGM9121 containing *hrcL* including the stop codon and	This study

	lacking the start codon for generation of N-terminal fusions; Sm^R	
Level -1 constructs		
pAGB197	Level -1 construct; derivative of pAGM1311 containing the native *hrpB* operon promoter and *hrpB1*, *hrpB2*, *hrcJ* and *hrpB4*; Km^R	Hausner et al., 2019
pAGB478	Level -1 construct; derivative of pAGM1311 containing *hrcN* including the stop codon and lacking the start codon for generation of N-terminal fusions; Km^R	This study
pAGB488	Level -1 construct; derivative of pICH41021 containing *sfgfp* with a linker (AKLEGPAGL)-encoding sequence for generation of N-terminal fusions; Ap^R	Otten and Büttner, 2021a
pAGB660	Level -1 construct; derivative of pAGM1311 containing *hrcL*, *hrcN*, *hrpB7*, *hrcT* and *hrcC* downstream of the native *hrpA* operon promoter deleted in *hrcN/L*; Km^R	This study
pAGB674	Level -1 construct; derivative of pAGM1311 containing *hrcL*, *hrcN*, *hrpB7*, *hrcT* and *hrcC* downstream of the native *hrpA* operon promoter deleted in *hrcN*; Km^R	This study
pAGB776	Level -1 construct; derivative of pAGM1311 containing the *hrpB* operon promoter upstream of *hrpB1*, *hrpB2*, *hrcJ* and *hrpB4* with a frameshift mutation in *hrpB4*; Km^R	Otten and Büttner, 2021a
pAGB805	Level -1 construct; derivative of pICH41021 containing *hrcQ* without stop codon for generation of C-terminal fusions; Ap^R	This study
pAGB961	Level -1 construct; derivative of pAGM1311 containing *hrcL*, *hrcN*, *hrpB7*, *hrcT* and *hrcC* downstream of the native *hrpA* operon promoter deleted in *hrcL*; Km^R	This study
pAGB1000	Level -1 construct; derivative of pICH41021 containing a linker (2 x AKLEGPAGL)-encoding sequence; Ap^R	Otten et al., 2021b
pAGB1001	Level -1 construct; derivative of pICH41021 containing a linker (2 x *GGAGGAGG*)-encoding sequence; Ap^R	This study
pAGB1047	Level -1 construct; derivative of pICH41021 containing *sfgfp* for generation of N-terminal fusions; Ap^R	This study
pAGB1119	Level -1 construct; derivative of pAGM1311 containing *hrcL* including the stop codon and lacking the start codon for generation of N-terminal fusions; Km^R	This study
pAGB1153	Level -1 construct; derivative of pICH41021 containing *mKO*κ for generation of C-terminal fusions; Ap^R	Otten et al., 2021b
Level 0 constructs		
pAGB231	Level 0 construct; derivative of pICH41276 containing a transcriptional terminator; Sm^R	Hausner et al., 2019
pAGB232	Level 0 construct; derivative of pICH41276 containing a native transcriptional terminator of *Xe*; Sm^R	This study
pAGB249	Level 0 construct; derivative of pICH41295 containing the native *hrpD* operon promoter; Sm^R	Hausner et al., 2019

pAGB512	Level 0 construct; derivative of pICH41295 containing the native *hrpB* operon promoter; Sm^R	Otten and Büttner, 2021a
pAGB661	Level 0 construct; derivative of pICH41331 containing the *hrpA* and *hrpB* operons deleted in *hrcN/L*; Sm^R	This study
pAGB678	Level 0 construct; derivative of pICH41331 containing the *hrpA* and *hrpB* operons deleted in *hrcN*; Sm^R	This study
pAGB831	Level 0 construct; derivative of pICH41308 encoding sfGFP-HrcN with a linker (AKLEGPAGL); Sm^R	This study
pAGB965	Level 0 construct; derivative of pICH41331 containing the *hrpA* and *hrpB* operons deleted in *hrcL*; Sm^R	This study
pAGB1121	Level 0 construct; derivative of pICH41308 encoding sfGFP-2xGGAGGAGG-HrcL; Sm^R	This study
pAGB1172	Level 0 construct; derivative of pICH41308 encoding HrcQ-2xAKLEGPAGL-mKOκ; Sm^R	This study
pAGB1201	Level 0 construct; derivative of pICH 41331 containing the *hrpA* and *hrpB* operons deleted in *hrcN/L* and with a frameshift mutation in *hrpB4*; Sm^R	This study
pAGB1270	Level 0 construct; derivative of pICH 41331 containing the *hrpA* and *hrpB* operons deleted in *hrcN* and with a frameshift mutation in *hrpB4*; Sm^R	This study
Level 1 constructs		
pAGB155	Level 1 construct; derivative of pICH47751 containing the *hrpC*, *hrpD*, *hrpE* and *hpaB* operons; Ap^R	Hausner et al., 2019
pAGB156	Level 1 construct; derivative of pICH47761 containing the *hrpF* operon; Ap^R	Hausner et al., 2019
pAGB157	Level 1 construct; derivative of pICH47772 containing *xopA* and *hpaH*; Ap^R	Hausner et al., 2019
pAGB160	Level 1 construct; derivative of pICH47861 containing *hrpX*; Ap^R	Hausner et al., 2019
pAGB163	Level 1 construct; derivative of pICH47732 containing *hrpG**; Ap	Hausner et al., 2019
pAGB275	Level 1 construct; derivative of pICH47751 containing the *hrpC*, *hrpD*, *hrpE* and *hpaB* operons deleted in *hrcQ*; Ap^R	Hausner et al., 2019
pAGB662	Level 1 construct; derivative of pICH47811 containing the *hrpA* and *hrpB* operons deleted in *hrcN/L*; Ap^R	This study
pAGB681	Level 1 construct; derivative of pICH47811 containing the *hrpA* and *hrpB* operons deleted in *hrcN*; Ap^R	This study
pAGB839	Level 1 construct; derivative of pICH47781 containing *sfgfp-hrcN* downstream of the native *hrpB* operon promoter; Ap^R	This study
pAGB969	Level 1 construct; derivative of pICH47811 containing the *hrpA* and *hrpB* operons deleted in *hrcL*; Ap^R	This study
pAGB1122	Level 1 construct; derivative of pICH47781 encoding sfGFP-2xGGAGGAGG-HrcL downstream of the native *hrpB* operon promoter; Ap^R	This study
pAGB1173	Level 1 construct; derivative of pICH47781 containing HrcQ-2xAKLEGPAGL-mKOκ	This study

	downstream of the native *hrpD* operon promoter; Ap^R	
pAGB1202	Level 1 construct; derivative of pICH47811 containing the *hrpA* and *hrpB* operons deleted in *hrcN/L* and with a frameshift mutation in *hrpB4*, Ap^R	This study
pAGB1273	Level 1 construct; derivative of pICH47811 containing the *hrpA* and *hrpB* operons deleted in *hrcN* and with a frameshift mutation in *hrpB4*; Ap^R	This study
pAGB1342	Level 1 construct; derivative of pICH47742 encoding sfGFP-HrcN downstream of the native *hrpB* operon promoter; Ap^R	This study
Level M constructs		
pAGB168	Level M construct; derivative of pAGM8031 containing the *hrp* gene cluster; Sm^R	Hausner et al., 2019
pAGB273	Level M construct; derivative of pAGM8031 containing the *hrp* gene cluster with a deletion in *hrcQ*; Sm^R	Hausner et al., 2019
pAGB322	Level M construct; derivative of pAGM8079 containing *xopA*, *hpaH*, *hrcQ-sfgfp*, *hrpX* and *hrpG**; Sm^R	Hausner et al., 2019
pAGB666	Level M construct; derivative of pAGM8031 containing the *hrp* gene cluster with a deletion in *hrcL/N*; Sm^R	This study
pAGB737	Level M construct; derivative of pAGM8031 containing the *hrp* gene cluster with a deletion in *hrcN*; Sm^R	This study
pAGB762	Level M construct; derivative of pAGM8031 containing the *hrp* gene cluster with a deletion in *hrcN* and *hrcQ*; Sm^R	This study
pAGB764	Level M construct; derivative of pAGM8031 containing the *hrp* gene cluster with a deletion in *hrcL/N* and *hrcQ*; Sm^R	This study
pAGB847	Level M construct; derivative of pAGM8079 containing *xopA*, *hpaH*, *sfgfp-hrcN*, *hrpX* and *hrpG**; Sm^R	This study
pAGB932	Level M construct; derivative of pAGM8031 containing the *hrp* gene cluster with a frameshift mutation in *hrpB4*; Sm^R	Otten and Büttner, 2021a
pAGB973	Level M construct; derivative of pAGM8031 containing the *hrp* gene cluster with a deletion in *hrcL* and *hrcQ*; Sm^R	This study
pAGB979	Level M construct; derivative of pAGM8031 containing the *hrp* gene cluster with a deletion in *hrcL*; Sm^R	This study
pAGB1124	Level M construct; derivative of pAGM8079 containing *xopA*, *hpaH*, *sfgfp-2xGGAGGAGG-hrcL*, *hrpX* and *hrpG**; Sm^R	This study
pAGB1184	Level M construct; derivative of pAGM8079 containing *xopA*, *hpaH*, *hrcQ-2xAKLEGPAGL-mKOκ*, *hrpX* and *hrpG**; Sm^R	This study
pAGB1203	Level M construct; derivative of pAGM8031 containing the *hrp* gene cluster with a deletion in *hrcN/L*, *hrcQ* and a frameshift mutation in *hrpB4*; Sm^R	This study
pAGB1275	Level M construct; derivative of pAGM8031 containing the *hrp* gene cluster with a deletion in *hrcN* and a frameshift mutation in *hrpB4*; Sm^R	This study

pAGB1343	Level M construct; derivative of pAGM8079 containing *xopA*, *hpaH*, *hrcQ-2xAKLEGPAGL-mKOκ*, *hrpX, hrpG** and *sfgfp-hrcN*; Sm^R	This study
pAGB1394	Level M construct; derivative of pAGM8031 containing the *hrp* gene cluster with a deletion in *hrcN/L* and a frameshift mutation in *hrpB4*; Sm^R	This study
Level P constructs		
pAGB279	Level P construct; derivative of pICH75322 containing the *hrp* gene cluster, *xopA*, *hpaH*, *hrpX* and *hrpG**; Km^R	Hausner et al., 2019
pAGB324	Level P construct; derivative of pICH75322 containing the *hrp* gene cluster (with a deletion in *hrcQ*), *xopA*, *hpaH*, *hrcQ-sfgfp*, *hrpX* and *hrpG**; Km^R	Hausner et al., 2019
pAGB556	Level P construct; derivative of pICH75322 containing the *hrp* gene cluster (with *hrpA* to *hpaB* operons replaced by dummy modules), *xopA*, *hpaH*, *hrcQ-sfgfp*, *hrpX* and *hrpG**; Km^R	Hausner et al., 2019
pAGB765	Level P construct; derivative of pICH75322 containing the *hrp* gene cluster (with deletions in *hrcN* and *hrcQ*), *xopA*, *hpaH*, *hrcQ-sfgfp*, *hrpX* and *hrpG**; Km^R	This study
pAGB767	Level P construct; derivative of pICH75322 containing the *hrp* gene cluster (with deletions in *hrcL/N* and *hrcQ*), *xopA*, *hpaH*, *hrcQ-sfgfp*, *hrpX* and *hrpG**; Km^R	This study
pAGB855	Level P construct; derivative of pICH75322 containing the *hrp* gene cluster (with a deletion in *hrcN*), *xopA*, *hpaH*, *sfgfp-hrcN*, *hrpX* and *hrpG**; Km^R	This study
pAGB866	Level P construct; derivative of pICH75322 containing the *hrp* gene cluster (with deletions in *hrcQ* and *hrcD*), *xopA*, *hpaH*, *hrcQ-sfgfp*, *hrpX* and *hrpG**; Km^R	Otten and Büttner, 2021a
pAGB990	Level P construct; derivative of pICH75322 containing the *hrp* gene cluster (with deletions in *hrcL* and *hrcQ*), *xopA*, *hpaH*, *hrcQ-sfgfp*, *hrpX* and *hrpG**; Km^R	This study
pAGB1126	Level P construct; derivative of pICH75322 containing the *hrp* gene cluster (with a deletion in *hrcL*), *xopA*, *hpaH*, *sfgfp-2xGGAGGAGG-hrcL*, *hrpX* and *hrpG**; Km^R	This study
pAGB1185	Level P construct; derivative of pICH75322 containing the *hrp* gene cluster (with a deletion in *hrcQ*), *xopA*, *hpaH*, *hrcQ-2xAKLEGPAGL-mKOκ*, *hrpX* and *hrpG**; Km^R	This study
pAGB1205	Level P construct; derivative of pICH75322 containing the *hrp* gene cluster (with deletions in *hrcN/L, hrcQ* and a frameshift mutation in *hrpB4*), *xopA*, *hpaH*, *hrcQ-sfgfp*, *hrpX* and *hrpG**; Km^R	This study
pAGB1260	Level P construct; derivative of pICH75322 containing the *hrp* gene cluste, *xopA*, *hpaH*, *hrpX, hrpG** and *xopB-2xAKLEGPAGL-mKOκ*; Km^R	This study
pAGB1261	Level P construct; derivative of pICH75322 containing the *hrp* gene cluster (with deletion in *hrcQ*), *xopA*, *hpaH*, *hrcQ-sfgfp*, *hrpX*, *hrpG** and *xopB-2xAKLEGPAGL-mKOκ*; Km^R	This study
pAGB1344	Level P construct; derivative of pICH75322 containing the *hrp* gene cluster (with deletions in	This study

	hrcN and *hrcQ*), *xopA*, *hpaH*, *hrcQ-2xAKLEGPAGL-mKOκ*, *hrpX*, *hrpG** and *sfgfp-hrcN*; Km^R	
pAGB1376	Level P construct; derivative of pICH75322 containing the *hrp* gene cluster (with deletions in *hrcN/L* and *hrcQ*), *xopA*, *hpaH*, *hrcQ-2xAKLEGPAGL-mKOκ*, *hrpX*, *hrpG** and *sfgfp-hrcN*; Km^R	This study

[1]Ap, ampicillin; Gm, gentamycin; Km, kanamycin; Nal, nalidixic acid; Rif, rifampicin; Sm, spectinomycin; R, resistant.

References

Canteros, B.I. (1990). Diversity of plasmids and plasmid-encoded phenotypic traits in *Xanthomonas campestris* pv. *vesicatoria*. PhD thesis. University of Florida.

Diepold, A., Amstutz, M., Abel, S., Sorg, I., Jenal, U., and Cornelis, G.R. (2010). Deciphering the assembly of the *Yersinia* type III secretion injectisome. EMBO J **29,** 1928-1940.

Dinh, T., and Bernhardt, T.G. (2011). Using superfolder green fluorescent protein for periplasmic protein localization studies. J Bacteriol **193,** 4984-4987.

Hausner, J., Jordan, M., Otten, C., Marillonnet, S., and Buttner, D. (2019). Modular cloning of the type III secretion gene cluster from the plant-pathogenic bacterium *Xanthomonas euvesicatoria*. ACS Synth Biol **8,** 532-547.

Karimova, G., Ullmann, A., and Ladant, D. (2001). Protein-protein interaction between *Bacillus stearothermophilus* tyrosyl-tRNA synthetase subdomains revealed by a bacterial two-hybrid system. J Mol Microbiol Biotechnol **3,** 73-82.

Karimova, G., Dautin, N., and Ladant, D. (2005). Interaction network among *Escherichia coli* membrane proteins involved in cell division as revealed by bacterial two-hybrid analysis. J Bacteriol **187,** 2233-2243.

Kousik, C.S., and Ritchie, D.F. (1998). Response of bell pepper cultivars to bacterial spot pathogen races that individually overcome major resistance genes. Plant Disease **82,** 181-186.

Lorenz, C., and Büttner, D. (2009). Functional characterization of the type III secretion ATPase HrcN from the plant pathogen *Xanthomonas campestris* pv. vesicatoria. J Bacteriol **191,** 1414-1428.

Lorenz, C., Hausner, J., and Büttner, D. (2012). HrcQ provides a docking site for early and late type III secretion substrates from *Xanthomonas*. PLoS ONE **7,** e51063.

Otten, C., and Büttner, D. (2021a). HrpB4 from *Xanthomonas campestris* pv. *vesicatoria* acts similarly to SctK proteins and promotes the docking of the predicted sorting platform to the type III secretion system. Cell Microbiol **23,** e13327.

Otten, C., Seifert, T., Hausner, J., and Büttner, D. (2021b). The contribution of the predicted sorting platform component HrcQ to type III secretion in *Xanthomonas campestris* pv. *vesicatoria* depends on an internal translation start site. Front Microbiol **12,** 752733.

Rossier, O., Van den Ackerveken, G., and Bonas, U. (2000). HrpB2 and HrpF from *Xanthomonas* are type III-secreted proteins and essential for pathogenicity and recognition by the host plant. Mol Microbiol **38,** 828-838.

Szczesny, R., Jordan, M., Schramm, C., Schulz, S., Cogez, V., Bonas, U., and Büttner, D. (2010). Functional characterization of the Xps and Xcs type II secretion systems from the plant pathogenic bacterium *Xanthomonas campestris* pv. *vesicatoria*. New Phytol **187,** 983-1002.

Weber, E., Engler, C., Grützner, R., Werner, S., and Marillonnet, S. (2011). A modular cloning system for standardized assembly of multigene constructs. PLoS ONE **6,** e16765.

Wengelnik, K., Rossier, O., and Bonas, U. (1999). Mutations in the regulatory gene *hrpG* of *Xanthomonas campestris* pv. vesicatoria result in constitutive expression of all *hrp* genes. J Bacteriol **181,** 6828-6831.

Yanisch-Perron, C., Vieira, J., and Messing, J. (1985). Improved M13 phage cloning vectors and host strains: nucleotide sequences of the M13mp18 and pUC19 vectors. Gene **33,** 103-119.

Table S2 Primers used in this study.

**Name**[1]	**Sequence**[2]
**Primer for pBRM and BACTH constructs**	
hrcL-TATG-F	TTT **GGTCTC** T *TATG* CGTCTGTGGTTGAGGTCAACG
hrcL-GGTG-R	TTT **GGTCTC** T *CACC* GCCAGCATCCGCCGGCTC
hrcL-Δ2-40-TATG-F	TTT **GGTCTC** T *TATG* TGCGAACAGGCCCTGAGCCAG
hrcL-Δ150-233-CACC-R	TTT **GGTCTC** T *CACC* GGCCTCGTCCAGGGCACCTTC
hrcN-Δ2-95-TATG-F	TTT **GGTCTC** T *TATG* TTGGCGGTGCCCGTTGGAC
hrcN-CACC-R	TTT **GGTCTC** T *CACC* CGCATCGTCGGTCACGCTGGT
hrcQ-+30aa-TATG-F	TTT **GGTCTC** T *TATG* CTAACCGAGCAGAGCCAGAC
hrcQ-CACC-R	TTT **GGTCTC** T *CACC* GGCATCTGCATGCGTGCTCTC
GentR-TGTG-F	TT **GAAGAC** TT *TGTG* TTAGGTGGCGGTACTTGGGTC
GentR-CTGT-R	TT **GAAGAC** TT *ACAG* TTGACATAAGCCTGT
pUTmod-res-CTGT-F	TT **GAAGAC** TT *CTGT* ACTGTCAGACCAAGTTTACTCATATATACTTTAG
pUTmod-res-ACAC-R	TT **GAAGAC** TT *CACA* TTTCCCCGAAAAGTGCCAC
sfGFP-pB-F	TTT **GGTCTC** T *TATG* GCTAAGCTGGAAGGCCCG
sfGFP-pB-R	AAA **GGTCTC** T *CACC* TCATCATTTGTACAG
**Primer for MoClo constructs**	
hrcN-Del-MoClo-F	P-CAACCGACCGACCAGCTCAGTG
hrcN-Del-MoClo-R	P-GTCAGTGCCGACGACTTCGACCAC
hrcNL-Del-MoClo-F	TTT **GAAGAC** AA *CTCA* ACAT TCAA CGAAAATACGCTGGAACTGCTG
hrcNL-Del-MoClo-R	TTT **GAAGAC** AA *CTCG* GGCA GGCTCACGCATCGTCGGTCAC
hrcL-MoClo-F1	TTT **GAAGAC** AA *CTCA* ACAT TCAA CGCCCGATGCGATC
hrcL-Del-MoClo-R1	TTT **GAAGAC** AA *ATCG* ACCAGCGTCTGTGCACGTG
hrcL-Del-MoClo-F2	TTT **GAAGAC** AA *CGAT* ACGGGCGTGTTCGAGACTG
hrcL-MoClo-R2	TTT **GAAGAC** AA *CTCG* GTCT CCAGCAGGGGCGTC
hrcN-CTM-MoClo-F	TTT **GGTCTC** T *ACAT* CCTG CTGGCTGAGACGCCCCTGCTG
hrcN-CTM-MoClo-R	TTT **GGTCTC** T *ACAA* AAGC TCACGCATCGTCGGTCACGCTGGTC
hrcL-CTM-MoClo-F	TTT **GAAGAC** AA *CTCA* ACAT CCTG CGTCTGTGGTTGAGGTCAACG
hrcL-CTM-MoClo-R	TTT **GAAGAC** AA *CTCG* ACAA AAGC TCA GCCAGCATCCGCCGGCTC
hrcQ-NTM-MoClo-F	TTT **GAAGAC** TT *AATG* TTCGGCGACCCACGCGCAGCAC
hrcQ-NTM-MoClo-R	TTT **GAAGAC** TT *TAGC* GGCATCTGCATGCGTGCTCTCCG
xopN-NTM-F1	TTT **GAAGAC** AA CTCA ACAT AATG AAGGCAGAGCTCACACG
xopB-NTM-R1	TTT **GAAGAC** AA ACGG GACACCAGCTCTAGTC
xopB-NTM-F2	TTT **GAAGAC** AA CCGT CGAGATCCGGAGAAAGCG
xopB-NTM-R2	TTT **GAAGAC** AA CTCG ACAA TAGC CGGCTCAGGCGCGGGTTGG
sfGFP-NTM-woLink-F	TTT **GAAGAC** AA *AATG* CGTAAAGGCGAGGAG
sfGFP-NTM-woLink-R	TTT **GAAGAC** AA *TAGC* TTTGTACAGTTCATCCATACCATGCG
MoClo-LinkDiepold-5-3	TTT **GAAGAC** AA *GCTA* AGGGTGGCGCAGGCGGTGCCGGAGGTGGAGGTGCTGGAGGCGC GGGTGG *CCTG* TT **GTCTTC** AAA
MoClo-LinkDiepold-3-5	TTT **GAAGAC** AA *CAGG* CCACCCGCGCCTCCAGCACCTCCACCTCCGGCACCGCCTGCGCCA CCCT *TAGC* TT **GTCTTC** AAA
termX2-MoClo-F	TTT **GAAGAC** TT *GCTT* CTTCGCGCCGTCCGCATCCCGC
termX2-MoClo-R	TTT **GAAGAC** TT *AGCG* CTGAGCGCGCATCATGCCAC

[1] NTM, N-terminal module, allows fusion with a reporter gene at the 3' end; CTM, C-terminal module, allows fusion with a reporter gene at the 5' end; F, forward; R, reverse.

[2] P, phosphorylated 5' end; *Bsa*I and *Bpi*I recognition sites are shown in bold and overhangs generated by *Bsa*I and *Bpi*I restriction in italics.

Table S3 Summary of studies with fluorescent fusions of T3S system components from *Xe.*

Fluorescent fusion[1]	Linker[2]	Protein synthesis[3]	Protein function[4]	Fluorescent foci formation[5]
**OM secretin HrcC**				
HrcC-sfGFP	AKLEGPAGL	(+)	+	-
sfGFP-HrcC	AKLEGPAGL	+	+/-	-
	2x AKLEGPAGL	+	+	+/-
	2x GGAGGAGG	+	(+)	+/-
HrcC-mCherry	2x AKLEGPAGL	+	+	+*
	2x GGAGGAGG	+	+	+*
HrcC-TagBFP	2x AKLEGPAGL	+	+	-
HrcC-sfTq2ox	2x AKLEGPAGL	+	+	-
**IM ring components**				
HrcD-sfGFP	AKLEGPAGL	-	+	-
	2x AKLEGPAGL	-	+	-
	2x GGAGGAGG	-	+	-
sfGFP-HrcD	AKLEGPAGL	(+)	-	-
	2x AKLEGPAGL	-	-	-
	2x GGAGGAGG	-	-	-
sfGFP-HrcJ	AKLEGPAGL	(+)	(+)	-
HrcJ-sfGFP	2x AKLEGPAGL	+/-	-	-
	2x GGAGGAGG	+/-	-	-
**ATPase HrcN and HrcL**				
HrcN-sfGFP	AKLEGPAGL	+	(+)	+
	2x AKLEGPAGL	+	(+)	+
HrcN- mKOκ	2x AKLEGPAGL	+	(+)	+
sfGFP-HrcL	2x AKLEGPAGL	(+)	+	-
	2x GGAGGAGG	(+)	+	-
**SctK-like linker protein HrpB4**				
sfGFP-HrpB4	AKLEGPAGL	-	+	-
HrpB4-sfGFP	AKLEGPAGL	(+)	-	-
**SctO-like protein HrpB7**				
sfGFP-HrpB7	AKLEGPAGL	-	+	-
HrpB7-sfGFP	AKLEGPAGL	-	+	-

[1] Reporter genes encoding the listed fluorescent fusion proteins were inserted into modular level P T3S gene cluster constructs which were deleted in the respective native genes.

[2] Fusion partners are separated by linker sequences. Letters refer to the amino acid sequence, 2 x, indicates that the linker sequence has been doubled.

[3] Protein stability was analysed in *Xe* strain 85*Δ*hrp*_fs*HAGX* containing the corresponding modular T3S gene cluster constructs. Bacteria were cultivated in minimal medium and protein extracts were analysed by immunoblotting. sfGFP and mCherry fusion proteins were detected by GFP- and mCherry-specific antibodies, respectively. mKOκ, sfTq2ox and TagBFP fusions contained a FLAG epitope tag for detection by a FLAG epitope-specific antibody. +, stable synthesis; (+), detection of cleavage products; +/- weak synthesis; -, proteins not detectable.

[4] Protein functions were analysed by infection studies. For this, strain 85*Δ*hrp*_fs*HAGX* containing modular T3S gene cluster constructs with deletions in the native *hrcC, hrcD, hrcN, hrcL, hrpB4* and *hrpB7* genes and encoding reporter fusions as indicated were infiltrated into leaves of susceptible and resistant pepper plants. Disease symptoms and the HR were monitored 7 and 2 days post infiltration, respectively. +, wild-type phenotype; (+) reduced disease symptoms and reduced HR; -, no visible plant reactions.

[5] Foci formation was analysed by fluorescence microscopy as described in Material and Methods. As control, foci were analysed in a strain lacking operons *hrpA* to *hpaB* and thus a functional T3S system. +, formation of fluorescent foci which were dependent on the presence of a functional T3S system; +/-, reduced foci formation; +*, formation of fluorescent foci which were independent of a functional T3S system, -, no fluorescent foci were detectable.

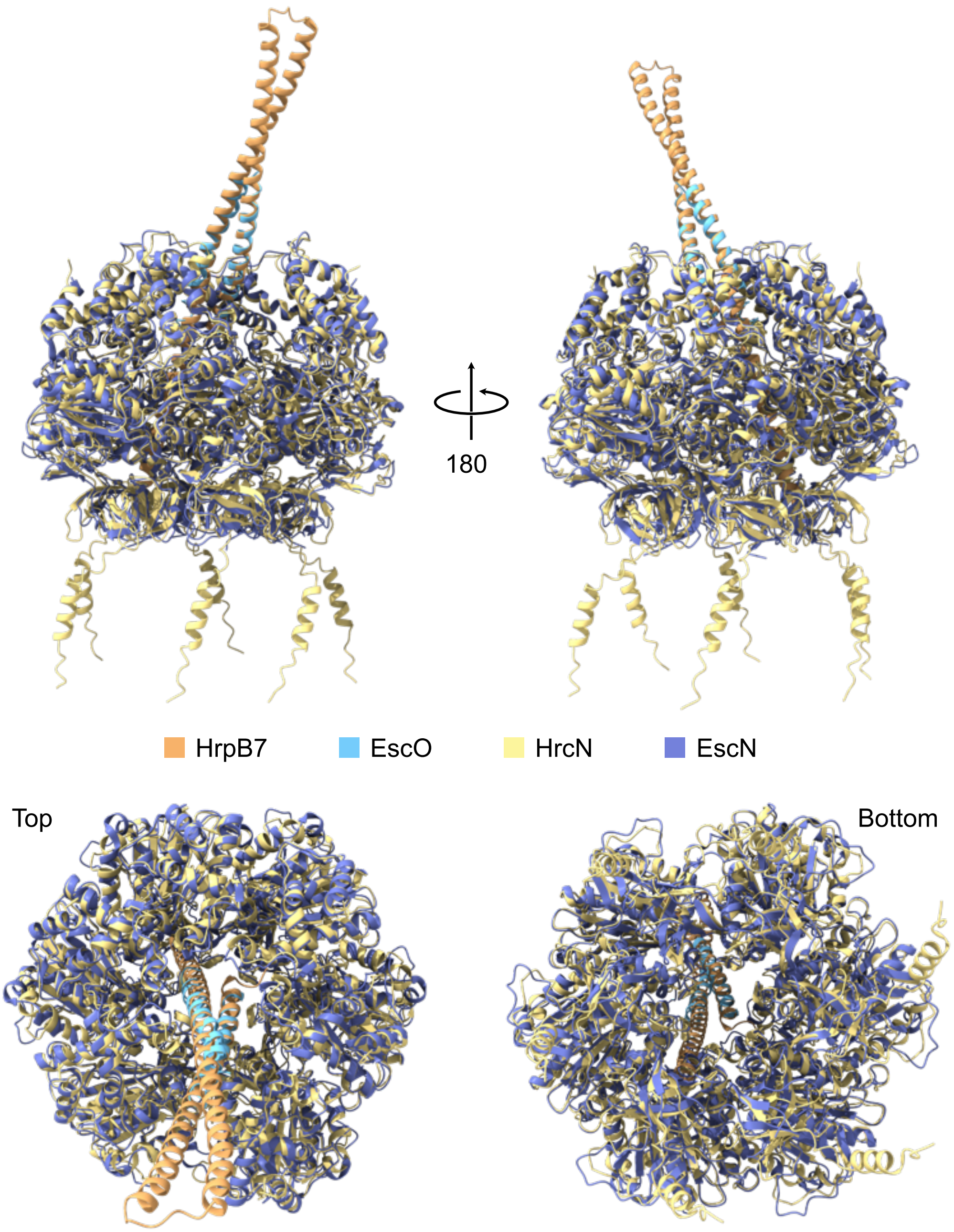

Figure S1
Otten and Büttner

Figure S1 Superimposition of the ATPase complexes from EPEC and *Xe.*

The figure displays the structural alignment of the hexameric ATPase complex from *Xe* consisting of HrcN and the predicted stalk protein HrpB7 with the structure of the EscN hexamer and the associated EscO stalk (PDB: 6NJP) from enteropathogenic *E. coli* (PDB: 6NJP). Structures of HrcN and HrpB7 were predicted by AlphaFold2 and superimposed onto the cryo-electron microscopy structure of the EscN-EscO complex using UCSF ChimeraX. Side views and views from the top and the bottom are shown. Different colours refer to HrpB7, EscO, HrcN and EscN as indicated.

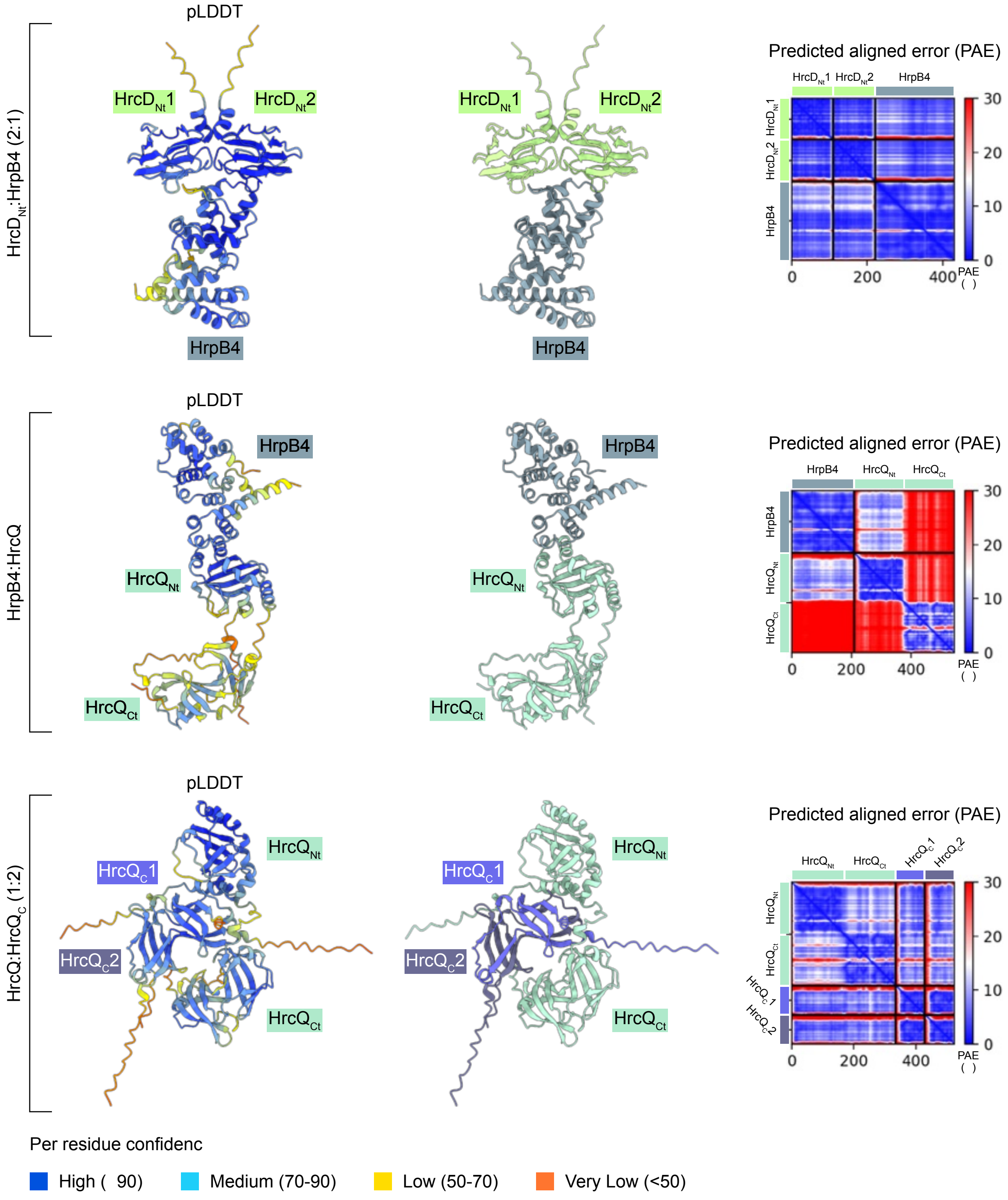

Figure S2
Otten and Büttner

Figure S2 Structural models of interacting sorting platform and IM ring components. AlphaFold 2 models are shown for a predicted dimer of the N-terminal cytoplasmic domain of HrcD ($HrcD_{Nt}$) associated with HrpB4, a HrpB4 - HrcQ dimer and the HrcQ/$HrcQ_C$ complex. The ribbon model on the left side shows the per-residue model confidence score (pLDDT, predicted local distance difference test) coloured from blue (very high confidence) to red (low confidence) as indicated. A predicted aligned error (PAE) plot on the right side shows regions of high (blue colour, low PAE value) and low (red colour, high PAE value) confidence for the predicted structures. N- and C-terminal domains are designated as Nt and Ct. HrcQ is predicted to interact with two molecules of $HrcQ_C$ indicated with “1” and “2”.

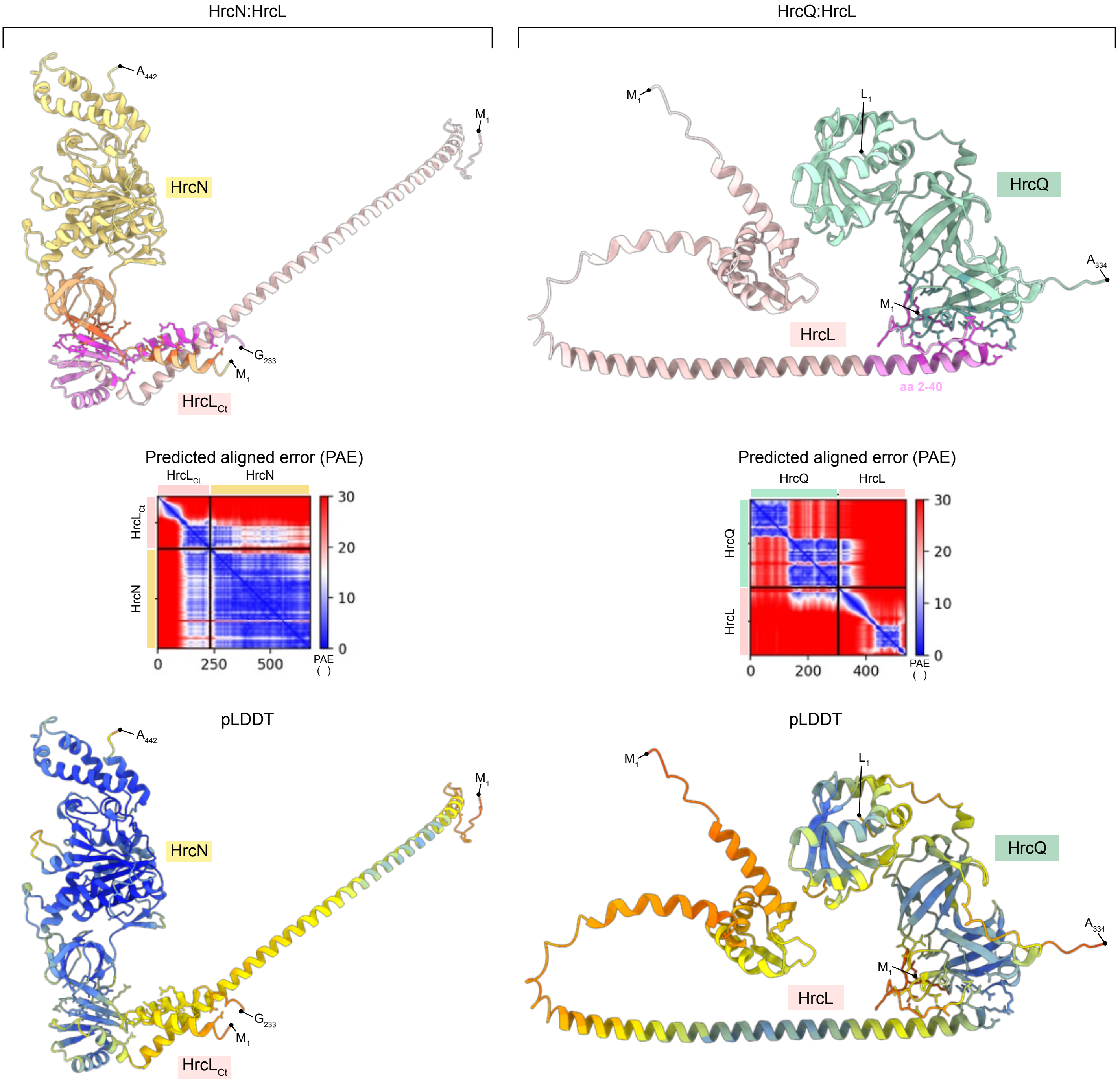

Figure S3
Otten and Büttner

Figure S3 Structural modeling of HrcN-HrcL and HrcL-HrcQ complexes*.*

Interaction sites HrcN-HrcL and HrcQ-HrcL complexes were predicted by AlphaFold 2. Numbers refer to amino acid positions, letters to amino acids. The ribbon models in the lower panels show the per-residue model confidence score (pLDDT, predicted local distance difference test) coloured from blue (very high confidence) to red (low confidence) as indicated. Predicted aligned error (PAE) plots show regions of high (blue colour, low PAE value) and low (red colour, high PAE value) confidence for the predicted structures.

	HrcD		HrpB4		HrcQ		HrcQ$_C$		HrcN		HrcL	
	BACTH	pull-down	BACTH	pull-down	BACTH	pull-down	BACTH	pull-down	BACTH	pull-down	BACTH	pull-down
HrcD	+ d	n.t.	+ d	+ d	+ d	n.t.	+ c	n.t.	n.t.	n.t.	n.t.	n.t.
HrpB4			+ d	n.t.	+ d	+ d	+ c	n.t.	- d	- d	- d	- d
HrcQ					+ c	+ b	+ c	+ c	+	+ b	+	+ b
HrcQ$_C$							+ c	+ c	+	n.t.	+	n.t.
HrcN									+	+ a	+	+ Co-IP a
HrcL											+	+ a

Figure S4
Otten and Büttner

Figure S4 Overview of interactions between predicted sorting platform components of *Xe.* The results of previous interaction studies with HrcD, HrpB4, HrcQ, $HrcQ_C$, HrcN and HrcL are summarized. Protein-protein interactions were analysed by BACTH assays (BACTH), *in vitro* pull-down experiments (pull-down) and co-immunoprecipitation in *Xe* (CoIP). +, interaction detected; -, no interaction detected; n.t., not tested. Symbols in bold refer to the results of the present study, letters to the references listed below.

[a] Lorenz, C., and Büttner, D. (2009). Functional characterization of the type III secretion ATPase HrcN from the plant pathogen *Xanthomonas campestris* pv. vesicatoria. J Bacteriol 191, 1414-1428.

[b] Lorenz, C., Hausner, J., and Büttner, D. (2012). HrcQ provides a docking site for early and late type III secretion substrates from *Xanthomonas*. PLoS ONE 7, e51063.

[c] Otten, C., Seifert, T., Hausner, J., and Büttner, D. (2021). The contribution of the predicted sorting platform component HrcQ to type III secretion in *Xanthomonas campestris* pv. *vesicatoria* depends on an internal translation start site. Front Microbiol 12, 752733.

[d] Otten, C., and Büttner, D. (2021). HrpB4 from *Xanthomonas campestris* pv. *vesicatoria* acts similarly to SctK proteins and promotes the docking of the predicted sorting platform to the type III secretion system. Cell Microbiol 23, e13327.

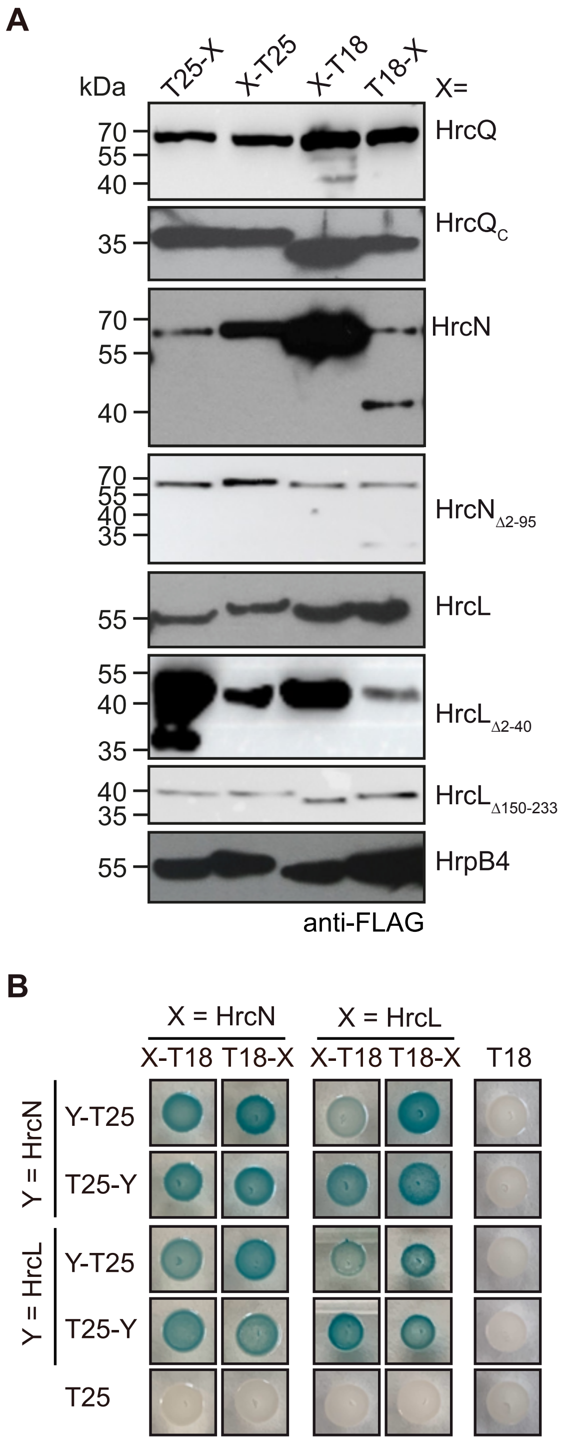

Figure S5
Otten and Büttner

Figure S5 Protein analysis and interaction studies with T18 and T25 fusions of HrcQ, HrcL and HrcN.

(A) Immunodetection of T18 and T25 fusion proteins. T18 and T25 fusions of HrcQ, $HrcQ_C$, HrcN, HrcL, HrpB4 and deletion derivatives of HrcL and HrcN as indicated were analysed in *E. coli* JM109 cells. For this, bacteria were grown in LB medium and gene expression was induced in the presence of IPTG. Total cell extracts were analysed by immunoblotting, using a FLAG epitope-specific antibody. Additional signals detected for $HrcN_{\Delta 2\text{-}95}$ and $HrcL_{\Delta 2\text{-}40}$ likely correspond to degradation products. T25 and T18 fusions of each protein were detected on the same blot. Fusion proteins containing HrcQ, $HrcN_{\Delta 2\text{-}95}$, $HrcL_{\Delta 2\text{-}40}$ and $HrcL_{\Delta 150\text{-}233}$ were detected using a chemiluminescence imager while blots with fusions of $HrcQ_C$, HrcN, HrpB4 and HrcL were visualized by exposure to X-ray films. All images were converted to black and white.

(B) HrcL and HrcN homo- and heterooligomerize. T18 and T25 fusions of HrcN and HrcL as indicated were analysed in *E. coli* DHM1 cells. As negative control, every fusion protein was tested against the T18 and T25 subdomain alone. Bacteria were cultivated on indicator plates and photographs were taken after five days. Experiments were performed at least three times with four independent transformants and led to similar results. One representative colony for each combination is shown.

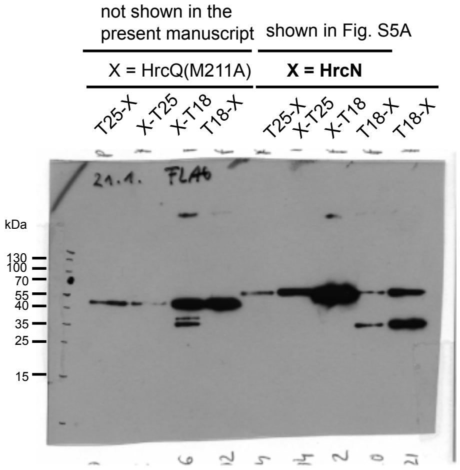

Original blot for the synthesis of HrcN fusions

X-ray film of one membrane
Edges of the membrane are not visible after developing of X-ray films.
The entire film on which the membrane was placed is shown.
Ten lanes including the marker were loaded.
10‘ exposure, original blot

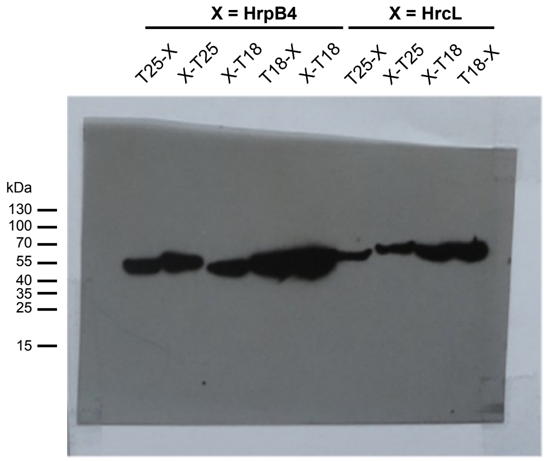

Original blot for the synthesis of HrpB4 and HrcL fusions

X-ray film of one membrane
Edges of the membrane are not visible after developing of X-ray films.
The entire film on which the membrane was placed is shown. Ten lanes including the marker were loaded.
5‘ exposure, original blot

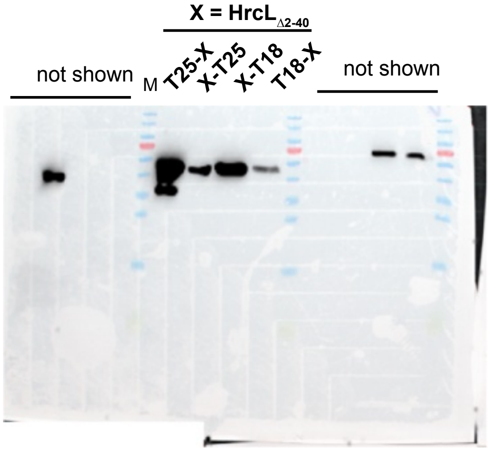

Original blot for the synthesis of HrcL derivatives

Chemiluminescence imager
20‘ exposure, original file.15 lanes were loaded.
(not modified, contrast was not changed)

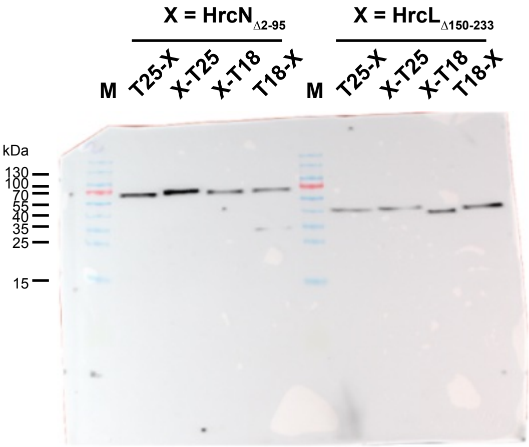

Original blot for the synthesis of HrcN + HrcL derivatives

Chemiluminescence imager
10‘ exposure, original file.10 lanes were loaded.
(not modified, contrast was not changed)

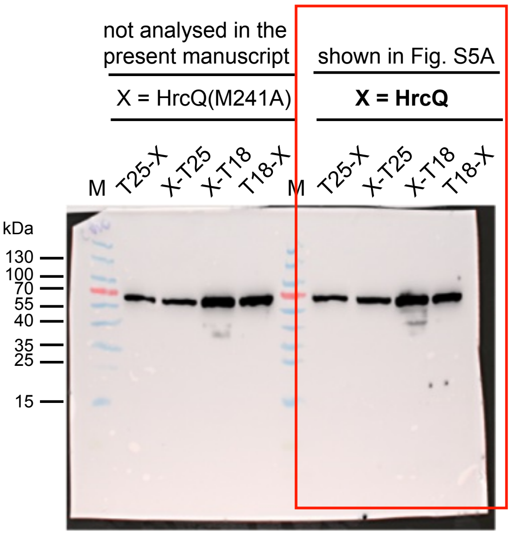

Original blot for the synthesis of HrcQ fusions

Chemiluminescence imager
10‘ exposure, original file.Ten lanes were loaded.
(not modified, contrast was not changed)

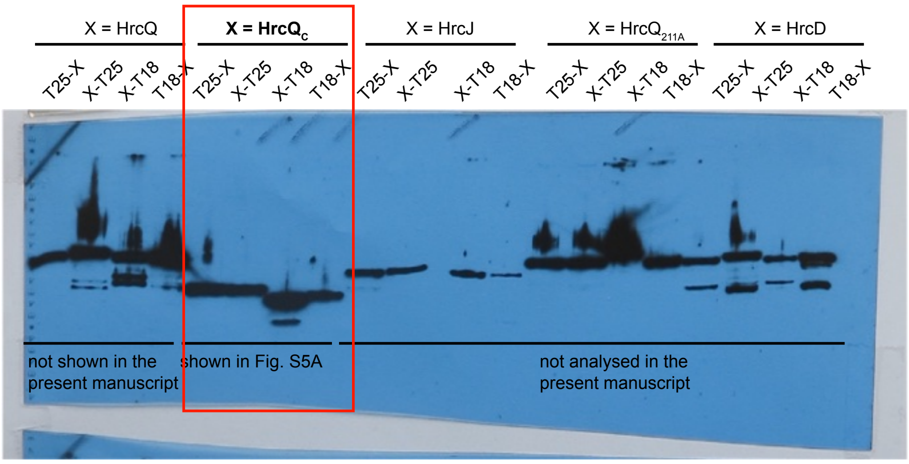

Original blot for the synthesis of HrcQc fusions

X-ray film of two membranes placed next to each other
(Edges of the membrane as requested are not visible after developing of X-ray films.
Therefore, the entire film is presented on which the blots were exposed.
Ten lanes per gel were loaded.)
10‘ exposure, original blot (not modified, contrast was not changed)

Figure S6
Otten and Buettner

Figure S6 Original blots for Figure S5

Immunodetection of T18 and T25 fusion proteins used for BACTH assays.

T18 and T25 fusions of HrcQ, $HrcQ_C$, HrcN, HrcL, HrpB4 and deletion derivatives of HrcL and HrcN as indicated were analysed in *E. coli* JM109 cells. For this, bacteria were grown in LB medium and gene expression was induced in the presence of IPTG. Total cell extracts were analysed by immunoblotting, using a FLAG epitope-specific antibody. Additional signals detected for $HrcN_{\Delta 2\text{-}95}$ and $HrcL_{\Delta 2\text{-}40}$ likely correspond to degradation products. Full uncut blots are shown and represent the signals presented in Figure S5. Fusion proteins containing HrcQ, $HrcN_{\Delta 2\text{-}95}$, $HrcL_{\Delta 2\text{-}40}$ and $HrcL_{\Delta 150\text{-}233}$ were detected using a chemiluminescence imager (Vilber Fusion FX Edge) as indicated. The overlay of the chemiluminescent signal with the image of the membrane taken under white light allows the visualization of the protein signals and the size marker on the same image. Blots showing fusions of $HrcQ_C$, HrcN, HrpB4 and HrcL were visualized by exposure to X-ray films. In case of $HrcQ_C$, a blue X-ray film was used. In figure S5 of the manuscript, all images were converted to black and white. The original files are shown. M, marker.

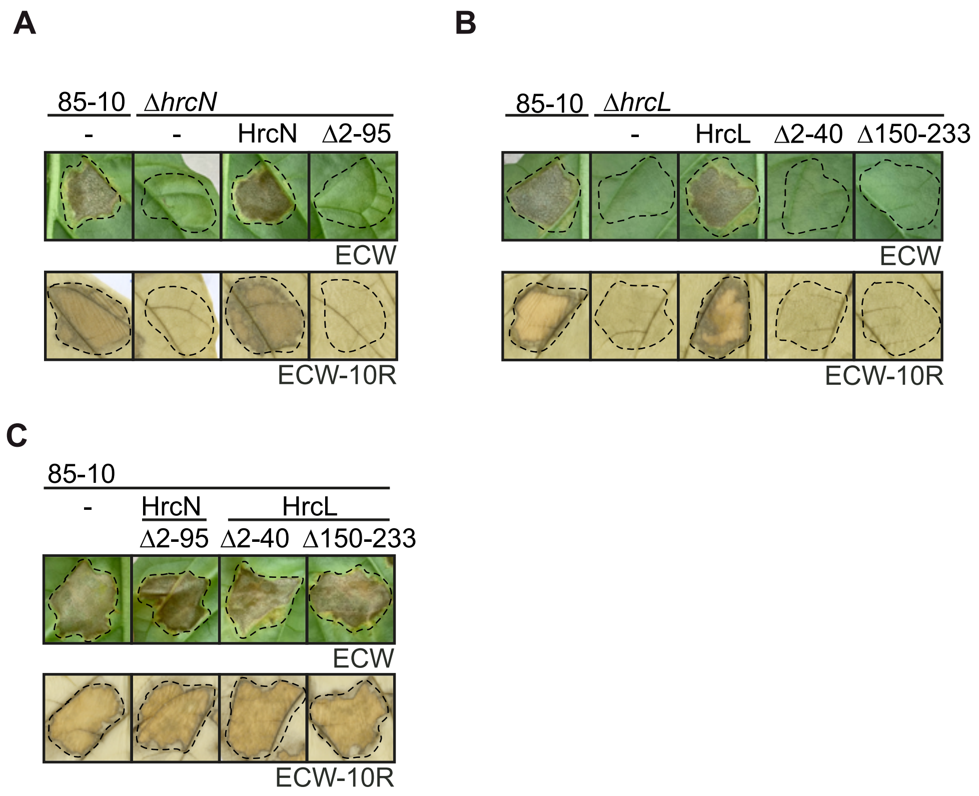

Figure S7
Otten and Büttner

Figure S7 Complementation studies with N- and C-terminal deletion derivatives of HrcL and HrcN.

(A) Deletion of the N-terminal region of HrcN abolishes protein function. *Xe* strains 85-10 and 85-10Δ*hrcN* (Δ*hrcN*) with or without (-) expression constructs encoding HrcN or $HrcN_{\Delta 2\text{-}95}$ (Δ2-95) as indicated were infiltrated into leaves of susceptible ECW and resistant ECW-10R pepper plants. Disease symptoms were photographed 10 dpi. For the better visualization of the HR, leaves were bleached in ethanol 2 dpi. Dashed lines indicate the infiltrated areas.

(B) HrcL derivatives with N- or C-terminal deletions are not functional. *Xe* strains 85-10 and 85-10Δ*hrcL* (Δ*hrcL*) with or without (-) expression constructs encoding HrcL or derivatives thereof deleted in amino acids 2 – 40 or 150 – 233 as indicated were infiltrated into leaves of susceptible ECW and resistant ECW-10R pepper plants. Plant reactions were documented as described in (A).

(C) Ectopic expression of HrcN or HrcL deletion derivatives does not interfere with pathogenicity of *Xe.* Strain 85-10 with or without (-) expression constructs encoding HrcN or HrcL deletion derivatives as indicated was infiltrated into leaves of susceptible and resistant pepper plants and plant reactions were documented as described in (A). All infection experiments were performed three times with similar results.

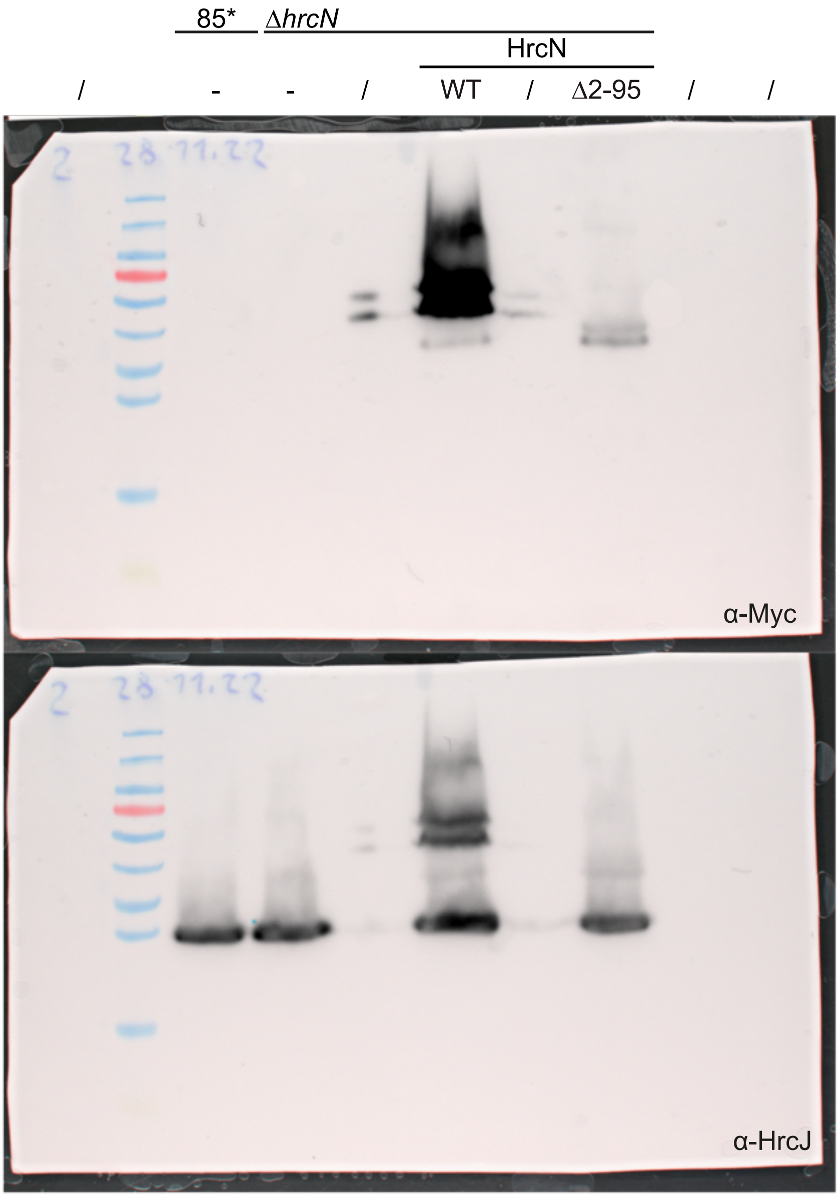

Figure S8

Otten and Büttner

Figure S8 Original blots for Figure 4B

Xe strains 85* and 85*Δ*hrcN* (Δ*hrcN*) with or without (-) expression constructs encoding HrcN (WT) or $HrcN_{\Delta 2\text{-}95}$ (Δ2-95) as indicated were grown in minimal medium and equal amounts of cell extracts were analysed by immunoblotting, using antibodies specific for the c-Myc epitope and HrcJ. The symbol "/" indicates empty lanes of the gel. Signals were detected using a chemiluminescence imager (Vilber Fusion FX Edge). The original files are shown.

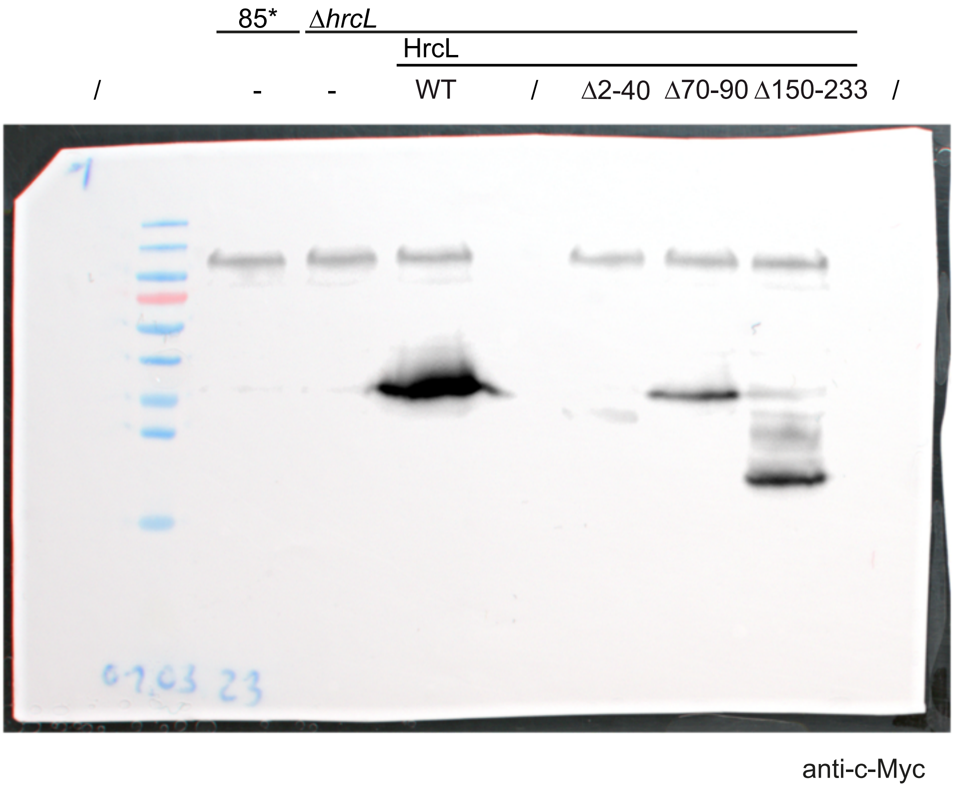

Figure S9

Otten and Büttner

Figure S9 Original blots for Figure 5B

Strains 85* and 85*Δ*hrcL* (Δ*hrcL*) with or without (-) expression constructs encoding HrcL-c-Myc (WT) or deletion derivatives thereof lacking amino acids 2 – 40, 70 – 90 (not analysed in the present manuscript) or 150 - 233 as indicated were grown in minimal medium and equal amounts of cell extracts were analysed by immunoblotting, using a c-Myc epitope-specific antibody. The symbol "/" indicates empty lanes of the gel. Signals were detected using a chemiluminescence imager (Vilber Fusion FX Edge). The original file is shown.

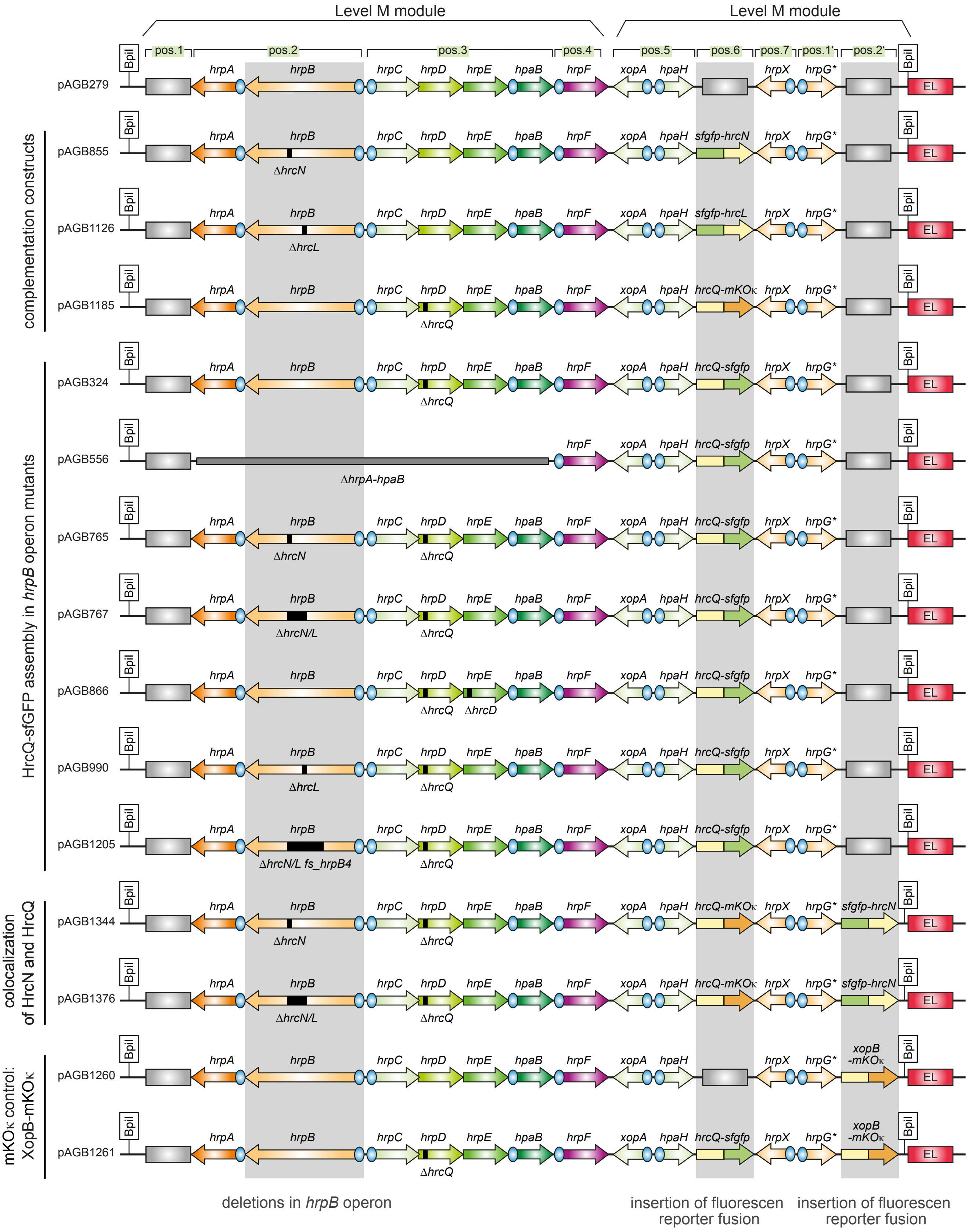

Figure S10
Otten and Büttner

Figure S10 Schematic overview of modular T3S gene cluster constructs used in this study. Modular level P T3S gene cluster constructs resulted from assembly of two level M constructs as indicated. Operons are represented by arrows, promoters by blue circles and grey rectangles indicate the position of dummy modules. The introduction of deletions is indicated by black boxes. *Bpi*I sites and the names of the constructs are indicated. EL, end-linker.

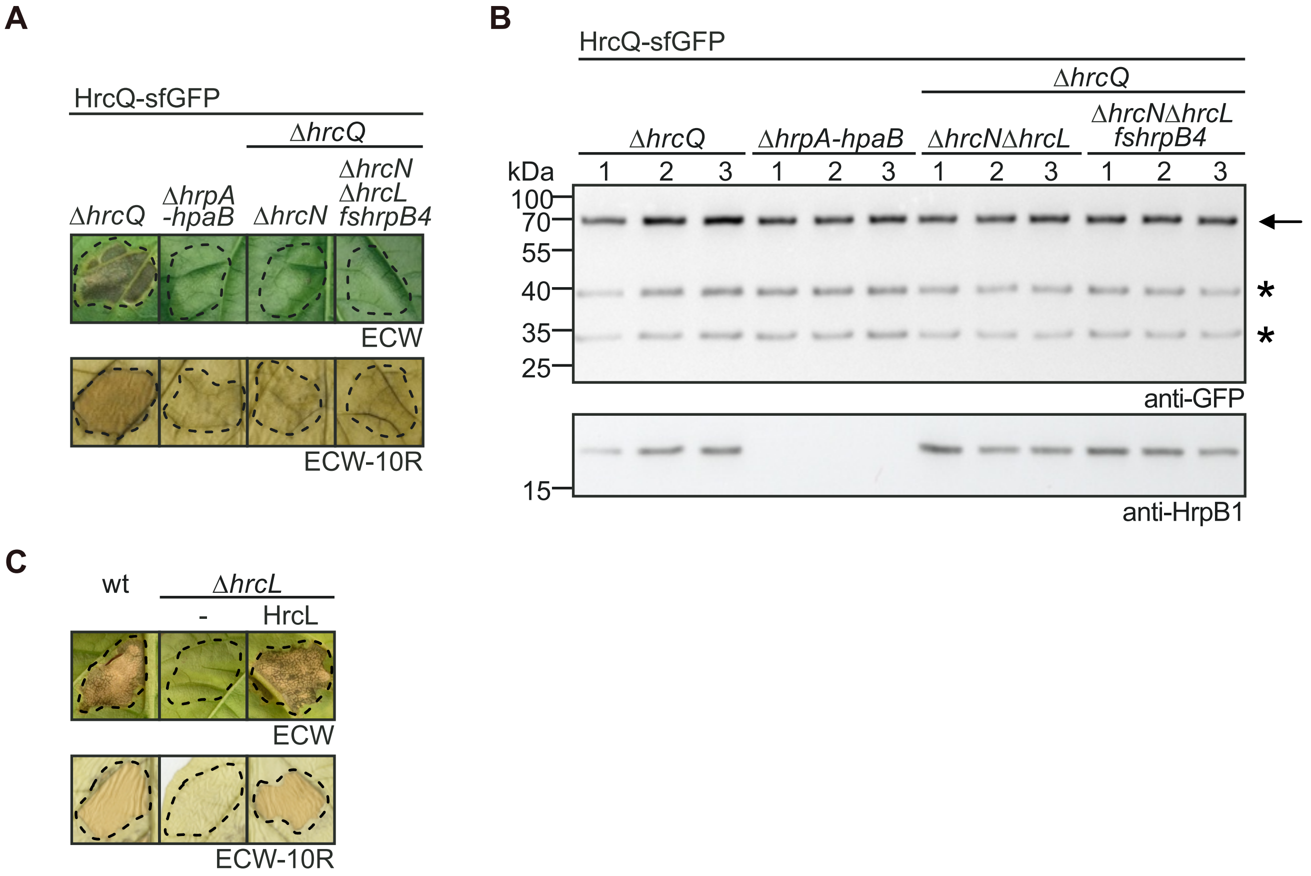

Figure S11
Otten and Büttner

Figure S11 Infection and protein studies with *Xe* strains containing modular T3S gene cluster constructs.

(A) HrcN, HrcL and HrpB4 are essential for pathogenicity. *Xe* strain 85*Δ*hrp*_fs*HAGX* with modular T3S gene cluster constructs encoding HrcQ-sfGFP and containing deletions in *hrcQ*, *hrpA* – *hpaB*, *hrcN*, *hrcL* and a frameshift (fs) mutation in *hrpB4* as indicated were infiltrated into leaves of susceptible ECW and resistant ECW-10R pepper plants. Disease symptoms were photographed 10 dpi. For the better visualization of the HR, leaves were bleached in ethanol 2 dpi. Dashed lines indicate the infiltrated areas.

(B) HrcQ-sfGFP is stably synthesized when encoded by modular T3S gene cluster constructs. *Xe* strains as listed in (A) were cultivated under T3S-permissive conditions, and equal amounts of cell extracts from three transconjugants (labeled as 1, 2 and 3) of each strain were analysed by immunoblotting, using a GFP-specific antibody. The upper signal indicated by an arrow corresponds to HrcQ-sfGFP, lower signals (indicated by asterisks) represent cleavage or degradation products. The blot was reprobed with an antibody specific for the periplasmic protein HrpB1 to show equal loading.

(C) Complementation of the *hrcL* mutant using a modular T3S gene cluster construct. Strains 85* (wt) and 85*Δ*hrp*_fs*HAGX* containing modular level P *hrp-HAGX* constructs with a deletion in *hrcL* (Δ*hrcL*) with or without (-) an *in cis* expression cassette encoding sfGFP-HrcL (HrcL) were infiltrated into leaves of susceptible ECW and resistant ECW-10R pepper plants. Plant reactions were documented as described in (A). Experiments were performed three times with similar results. Note that the sfGFP-HrcL fusion was not detectable by immunoblot analysis using a GFP-specific antibody, which was likely due to the cleavage of the sfGFP fusion partner (data not shown).

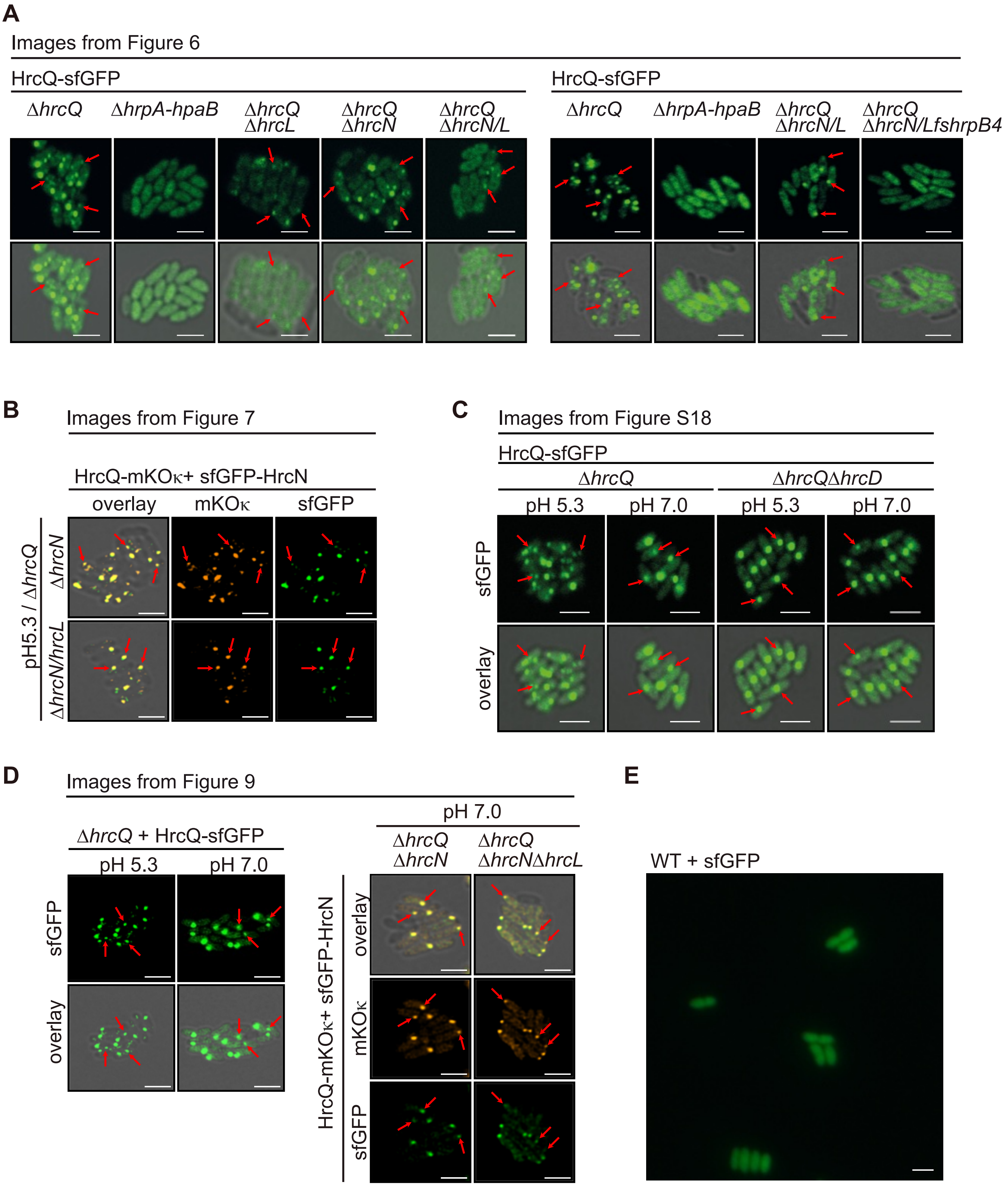

Figure S12
Otten and Büttner

Figure S12 Fluorescence microscopy imaging of HrcQ-sfGFP and GFP in *Xe*.

(A - D) Fluorescence microscopy images from Figure 6, 7, 9 and S18 are shown as indicated (see figure legends for details). Red arrows in each image indicate three foci with the weakest fluorescence which were included in the quantitative analyses. Weaker signals than the indicated threshold were not considered as foci. The scale bar corresponds to 2 µm.

(E) sfGFP localizes to the bacterial cytoplasm. Strain 85* encoding sfGFP under control of the *lac* promoter was incubated in minimal medium at pH 5.3 and bacteria were inspected by fluorescence microscopy. One representative image is shown. The scale bar corresponds to 2 µm.

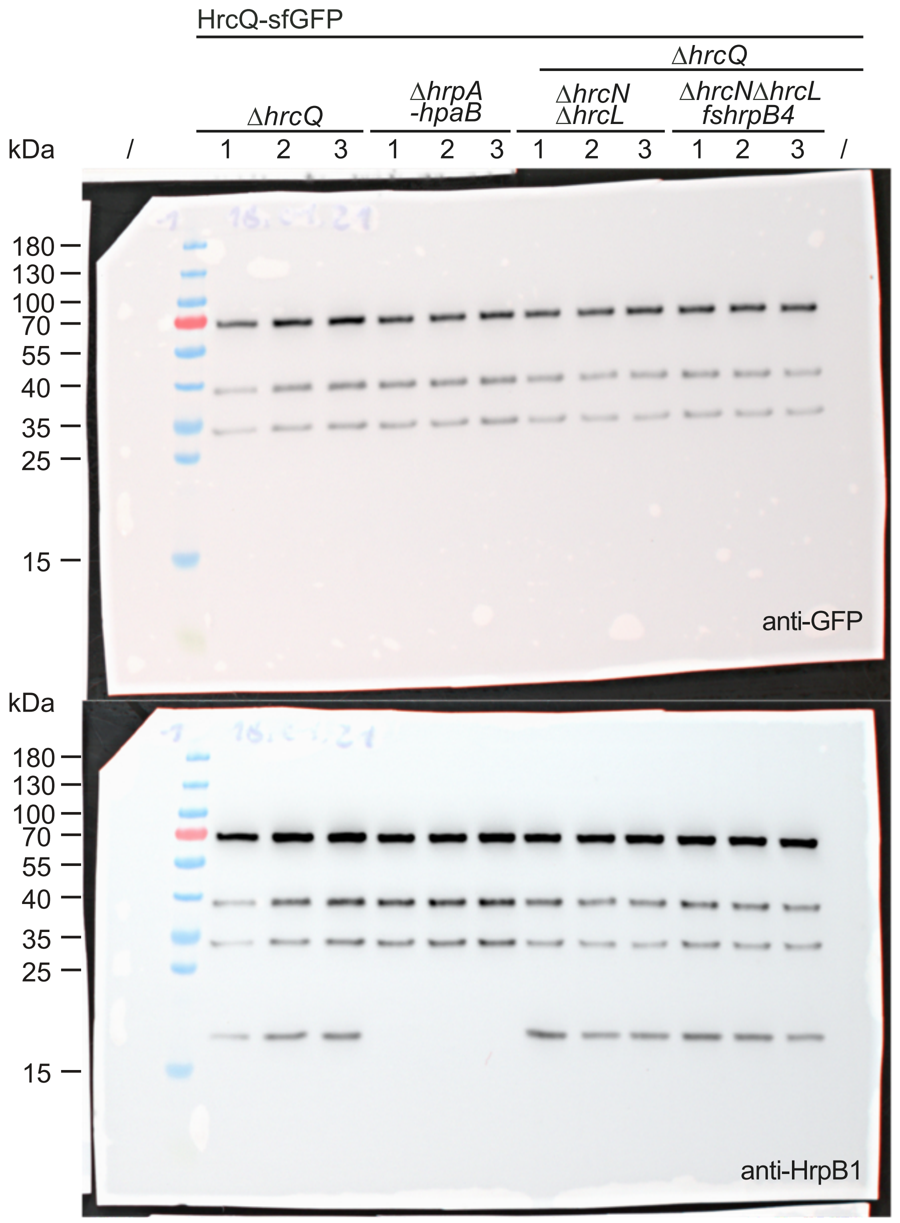

Figure S13

Otten and Büttner

Figure S13 Original blots for Figure S11

Xe strain 85*Δ*hrp*_fs*HAGX* with modular T3S gene cluster constructs encoding HrcQ-sfGFP and containing deletions in *hrcQ*, *hrpA* – *hpaB*, *hrcN*, *hrcL* and a frameshift (fs) mutation in *hrpB4* as indicated were cultivated under T3S-permissive conditions, and equal amounts of cell extracts from three transconjugants (labeled as 1, 2 and 3) of each strain were analysed by immunoblotting, using a GFP-specific antibody. The blot was reprobed with an antibody specific for the periplasmic protein HrpB1 to show equal loading. The symbol "/" indicates empty lanes of the gel. Signals were detected using a chemiluminescence imager (Vilber Fusion FX Edge). The original files are shown.

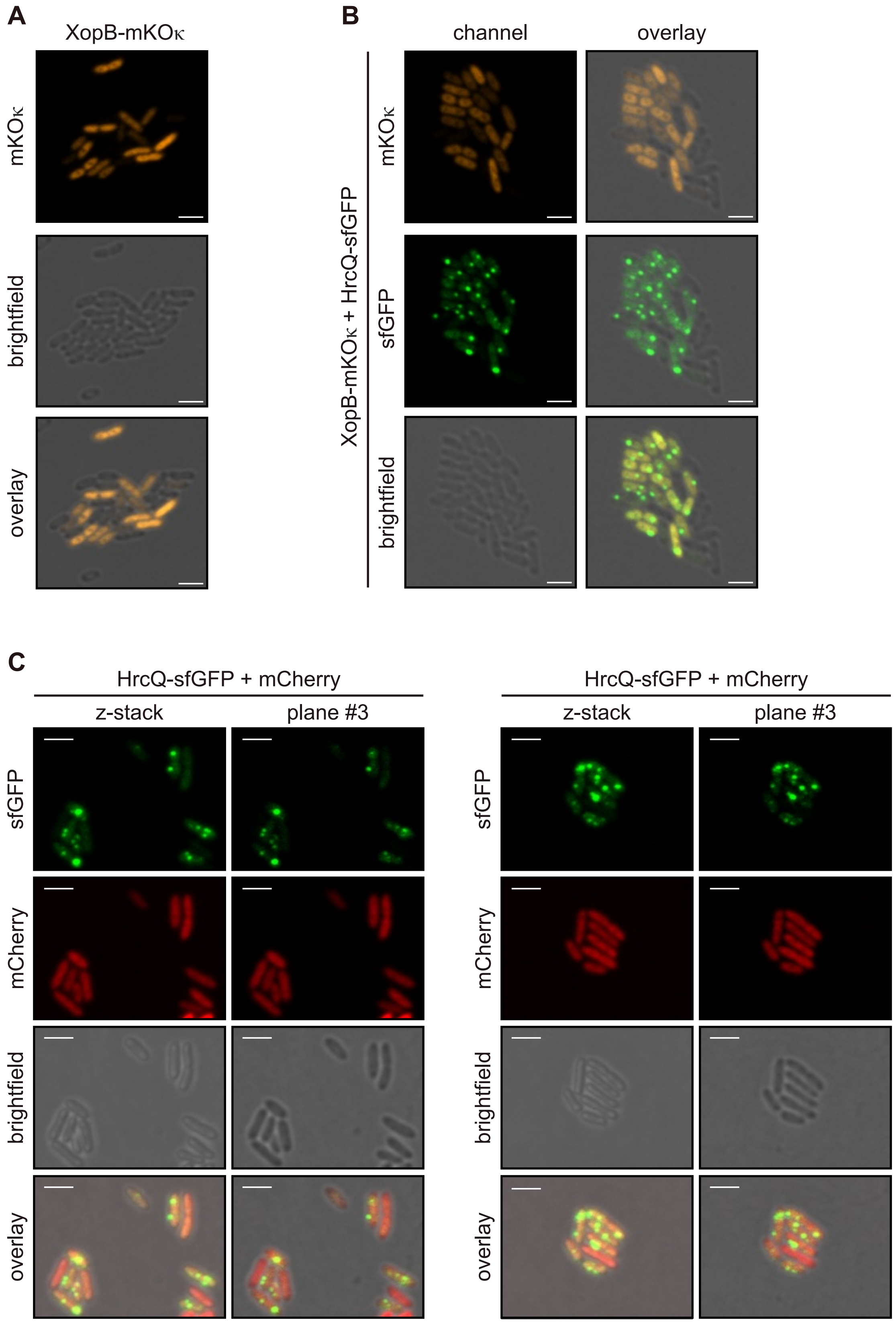

Figure S14
Otten and Büttner

Figure S14 Localization studies with XopB-mKO$_\kappa$.

(A) XopB-mKO$_\kappa$ localizes to the bacterial cytoplasm. *Xe* strain 85*Δ*hrp*_fs*HAGX* containing a modular T3S gene cluster construct encoding XopB-mKO$_\kappa$ was incubated under T3S-permissive conditions and analysed by fluorescence microscopy. mKOκ was excited using a white light laser at 551 nm and fluorescence signals were captured using HyD S detectors with emission detection windows at 563 nm. One representative image is shown. The scale bars correspond to 2 µm. The pictures in the lower panel result from an overlay of the fluorescent signals with the images of the brightfield channel. Since bacteria were cultivated overnight without antibiotics, some cells might have lost the plasmid, resulting in a lack of fluorescence.

(B) Colocalization studies with XopB-mKO$_\kappa$ and HrcQ-sfGFP. Strain 85*Δ*hrp*_fs*HAGX* containing a modular T3S gene cluster construct encoding XopB-mKO$_\kappa$ and HrcQ-sfGFP was incubated under T3S-permissive conditions and analysed by fluorescence microscopy using filter sets specific for sfGFP and mKO$_\kappa$. One representative image is shown. The scale bars correspond to 2 µm. The pictures in the lower panel result from an overlay of fluorescent signals with the images of the brightfield channel.

(C) Comparison of z-stacks and a single plane for the localization of HrcQ-sfGFP. Strain 85*Δ*hrp*_fs*HAGX* containing a modular T3S gene cluster construct encoding HrcQ-sfGFP and mCherry was analysed by fluorescence microscopy using HyD S detectors. A z-stack was created by capturing images from eight different planes of the same sample and merging them into a single image. A comparison of the z-stack with a single plane is shown, which displayed the strongest foci signals and was selected for foci counting.

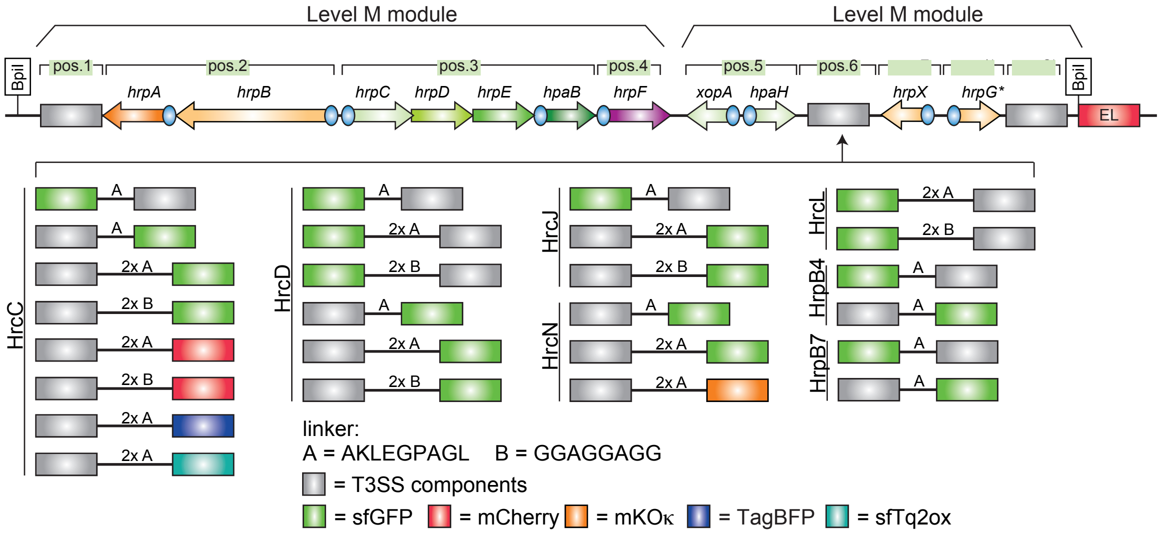

Figure S15
Otten and Büttner

Figure S15 Overview of fluorescent reporter fusions analysed for this study.

Fluorescent reporter fusions were inserted at position 6 of the modular T3S gene cluster construct as indicated. Arrows refer to *hrp* operons, blue circles to promoters and grey rectangles indicate the position of dummy modules. Translational fusions were generated with HrcC, HrcD, HrcN, HrcL, HrcJ, HrpB4 and HrpB7 using sfGFP, mCherry, mKOκ,TagBFP and sfTq2ox as reporter proteins as indicated. Both fusion partners were separated by single or duplicated linker sequences (A = AKLEGPAGI and B = GGAGGAGG) as indicated. Linkers were previously used for localization and assembly studies (Diepold et al., 2010[1]; Dinh and Bernhardt, 2011[2]). Proteins are represented by boxes. EL, end linker.

[1] Diepold, A., Amstutz, M., Abel, S., Sorg, I., Jenal, U., and Cornelis, G.R. (2010). Deciphering the assembly of the *Yersinia* type III secretion injectisome. EMBO J 29, 1928-1940.

[2] Dinh, T., and Bernhardt, T.G. (2011). Using superfolder green fluorescent protein for periplasmic protein localization studies. J Bacteriol 193, 4984-4987.

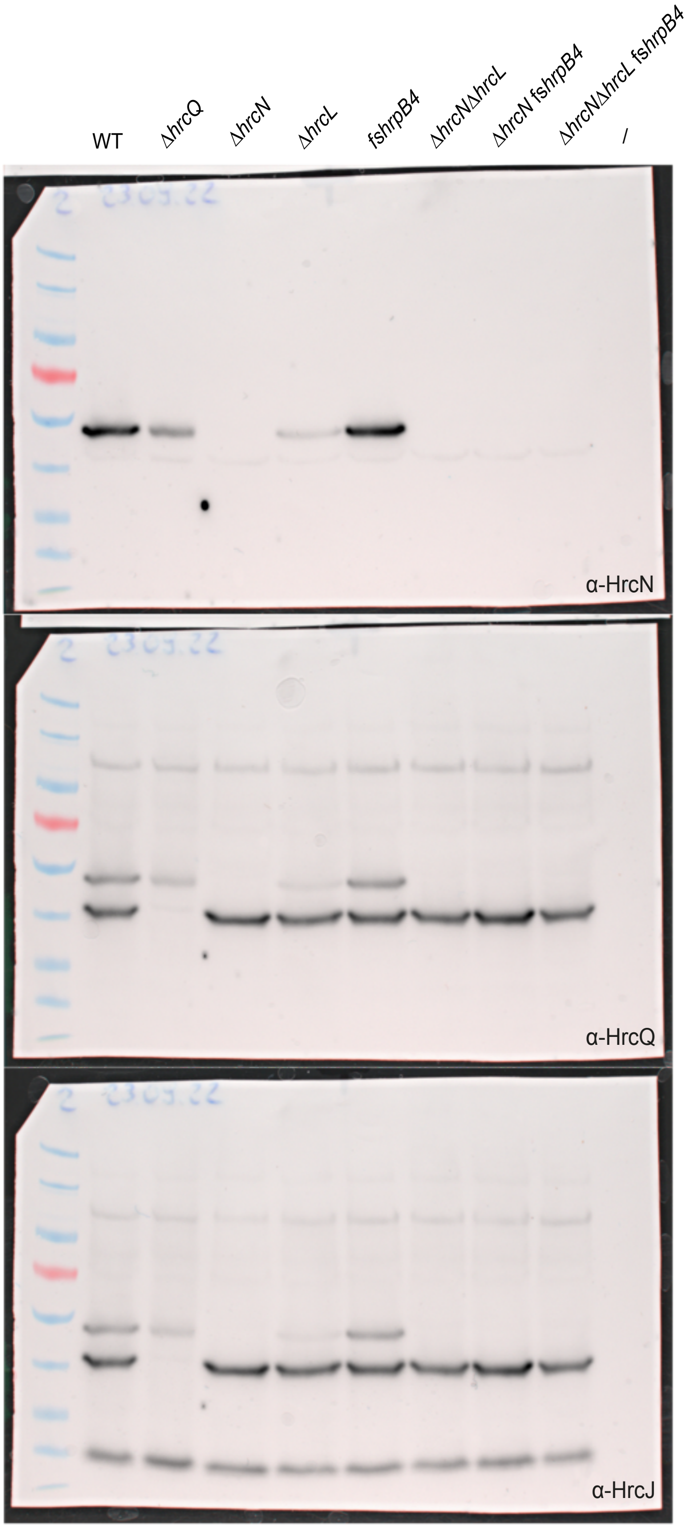

Figure S16

Otten and Büttner

Figure S16 Original blots for Figure 8A

Strain 85*Δ*hrp* containing the wild-type modular T3S gene cluster (WT) or derivatives thereof with deletions in *hrcQ, hrcN, hrcL* or a frameshift mutation (fs) in *hrpB4* as indicated were grown in minimal medium. Equal amounts of cell extracts from bacteria in the exponential growth phase were analysed by immunoblotting using antibodies specific for HrcN, HrcQ and HrcJ, respectively. Detection of the IM ring protein HrcJ served as loading control. The symbol "/" indicates empty lanes of the gel. Signals were detected using a chemiluminescence imager (Vilber Fusion FX Edge). The original files are shown.

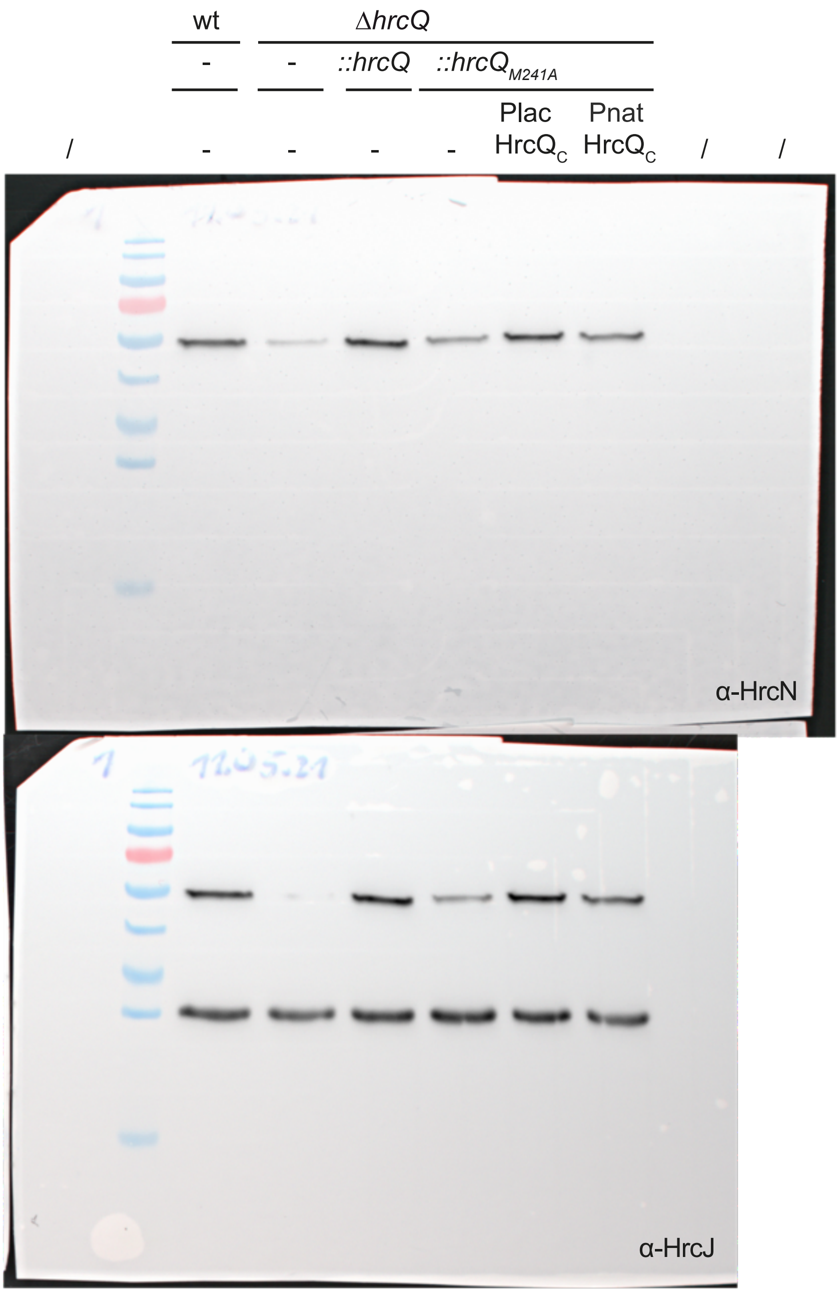

Figure S17

Otten and Büttner

Figure S17 Original blots for Figure 8B

The contribution of $HrcQ_C$ to HrcN stability was analysed in strains 85* (wt) and 85*Δ*hrcQ* (Δ*hrcQ*) with or without (-) chromosomal insertion of *hrcQ* or $hrcQ_{M241A}$ as indicated. For complementation studies, an expression construct encoding $HrcQ_C$ under control of the *lac* promoter was introduced into strain 85*Δ*hrcQ*::$hrcQ_{M241A}$ as indicated. Equal amounts of cell extracts were analysed by immunoblotting using HrcN- and HrcJ-specific antibodies. The symbol "/" indicates empty lanes of the gel. Signals were detected using a chemiluminescence imager (Vilber Fusion FX Edge). The original files are shown.

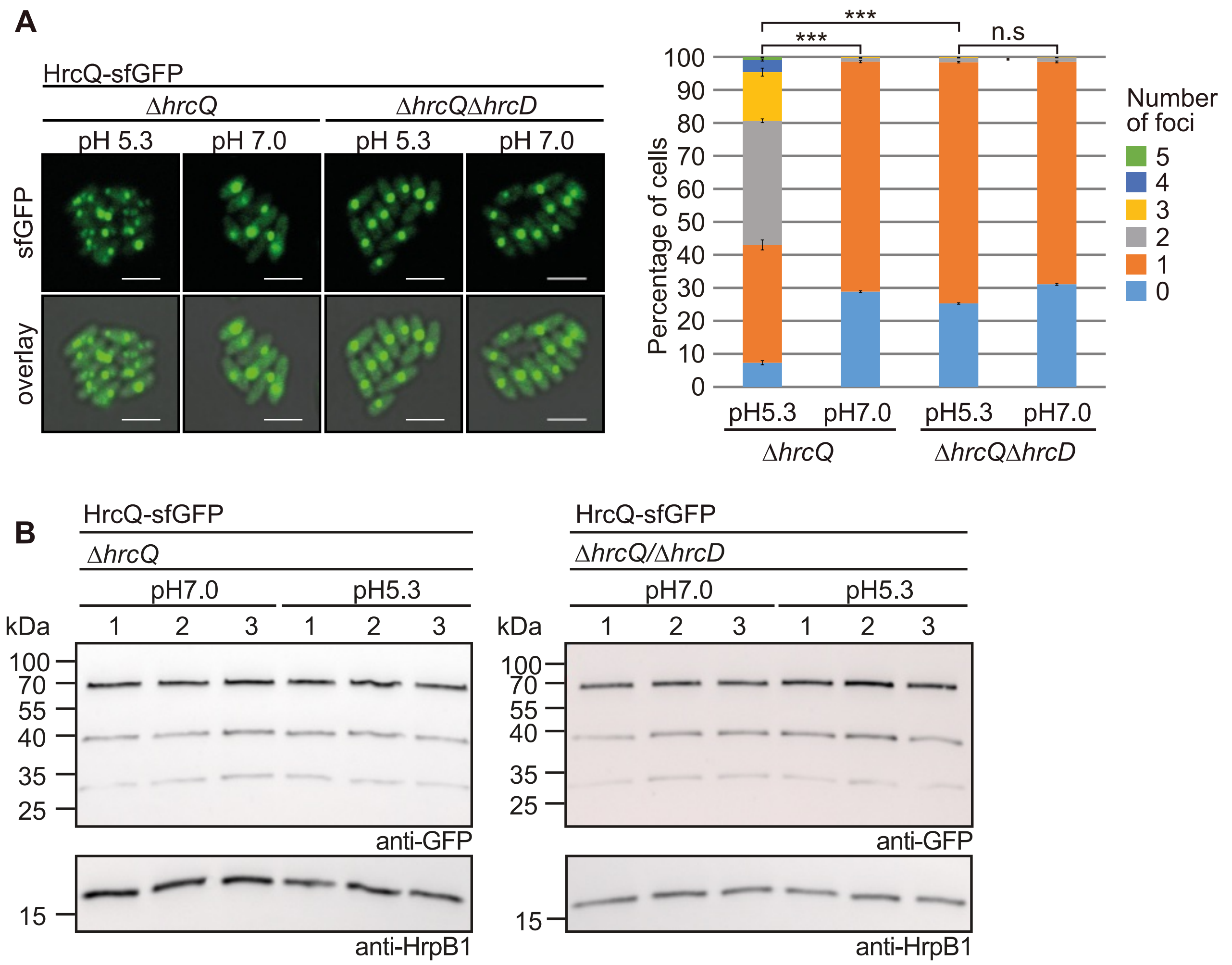

Figure S18
Otten and Büttner

Figure S18 Analysis of HrcQ-sfGFP complexes at pH 5.3 and pH 7.0.

(A) HrcQ-sfGFP foci formation at pH 7.0 is independent of the inner membrane ring component HrcD. Strain 85*Δ*hrp*_fs*HAGX* containing modular level P *hrp-HAGX* constructs with deletions in *hrcQ* and *hrcD* as indicated and encoding HrcQ-sfGFP were incubated in minimal medium at pH 5.3 and pH 7.0. Foci formation was monitored by fluorescence microscopy. One representative image for each strain is shown. The pictures in the lower panel show the overlay of the fluorescent signals with the images of the brightfield channel. The scale bar corresponds to 2 µm. Foci formation in approximately 300 cells per strain is summarized in a diagram. Significant differences between the number of foci with a *p* value of < 0.001 based on the results of χ^2 test are indicated by asterisks.

(B) HrcQ-sfGFP is stably synthesized when bacteria are cultivated in minimal medium at pH 5.3 and pH 7.0. Equal amounts of cell extracts from three transconjugants (labeled as 1, 2 and 3) of *Xe* strains as listed in (A) were analysed by immunoblotting, using a GFP-specific antibody. The upper signal indicated by an arrow corresponds to HrcQ-sfGFP, lower signals (indicated by asterisks) represent cleavage or degradation products. The blot was reprobed with an antibody specific for the periplasmic protein HrpB1 to show equal loading.

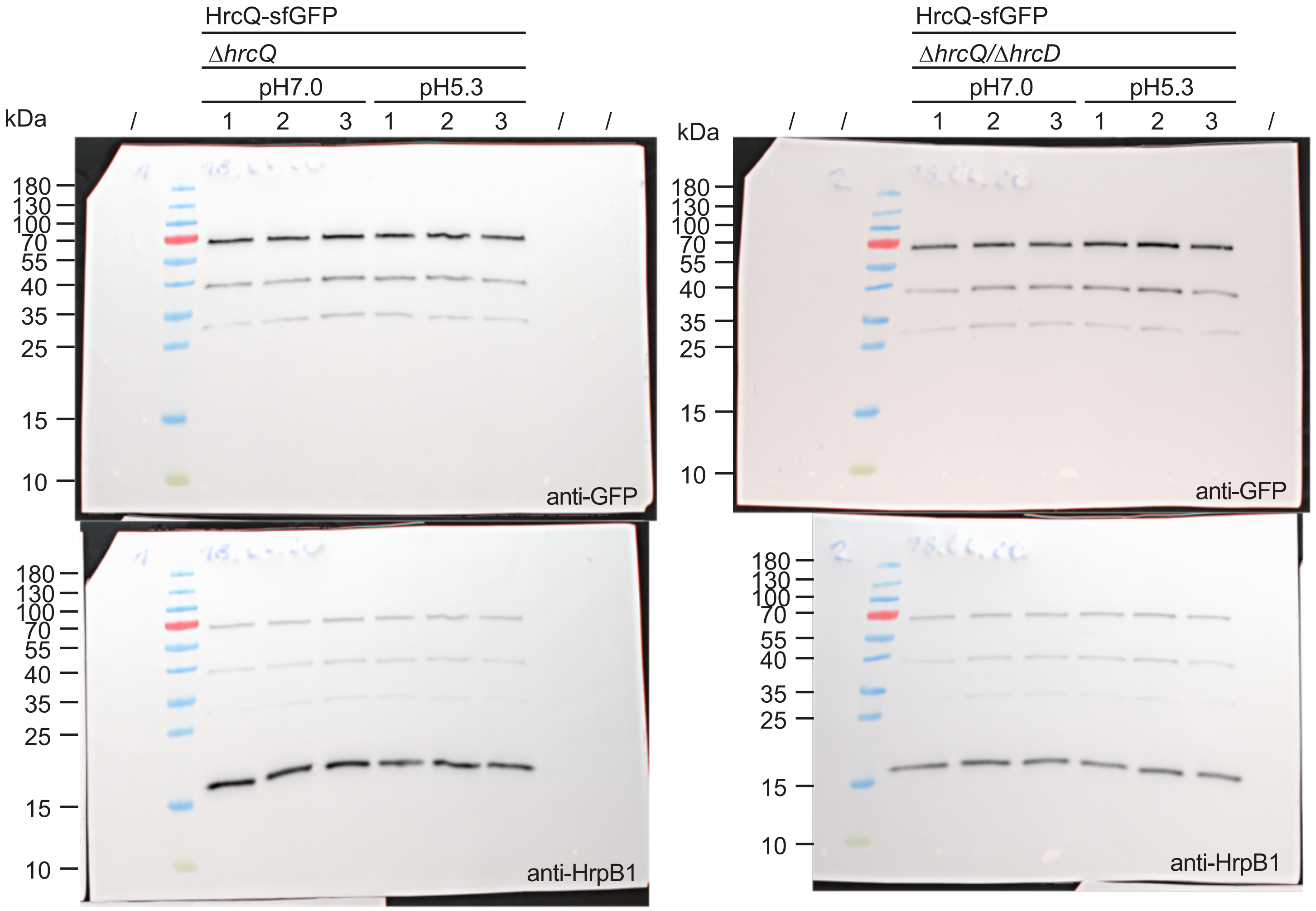

Figure S19

Otten and Büttner

Figure S19 Original blots for Figure S18

Strain 85*Δ*hrp*_fs*HAGX* containing modular level P *hrp-HAGX* constructs with deletions in *hrcQ* and *hrcD* as indicated and encoding HrcQ-sfGFP were incubated in minimal medium at pH 5.3 and pH 7.0. Equal amounts of cell extracts from three transconjugants (labeled as 1, 2 and 3) were analysed by immunoblotting, using a GFP-specific antibody. The blot was reprobed with an antibody specific for the periplasmic protein HrpB1 to show equal loading. The symbol "/" indicates empty lanes of the gel. Signals were detected using a chemiluminescence imager (Vilber Fusion FX Edge). The original files are shown.

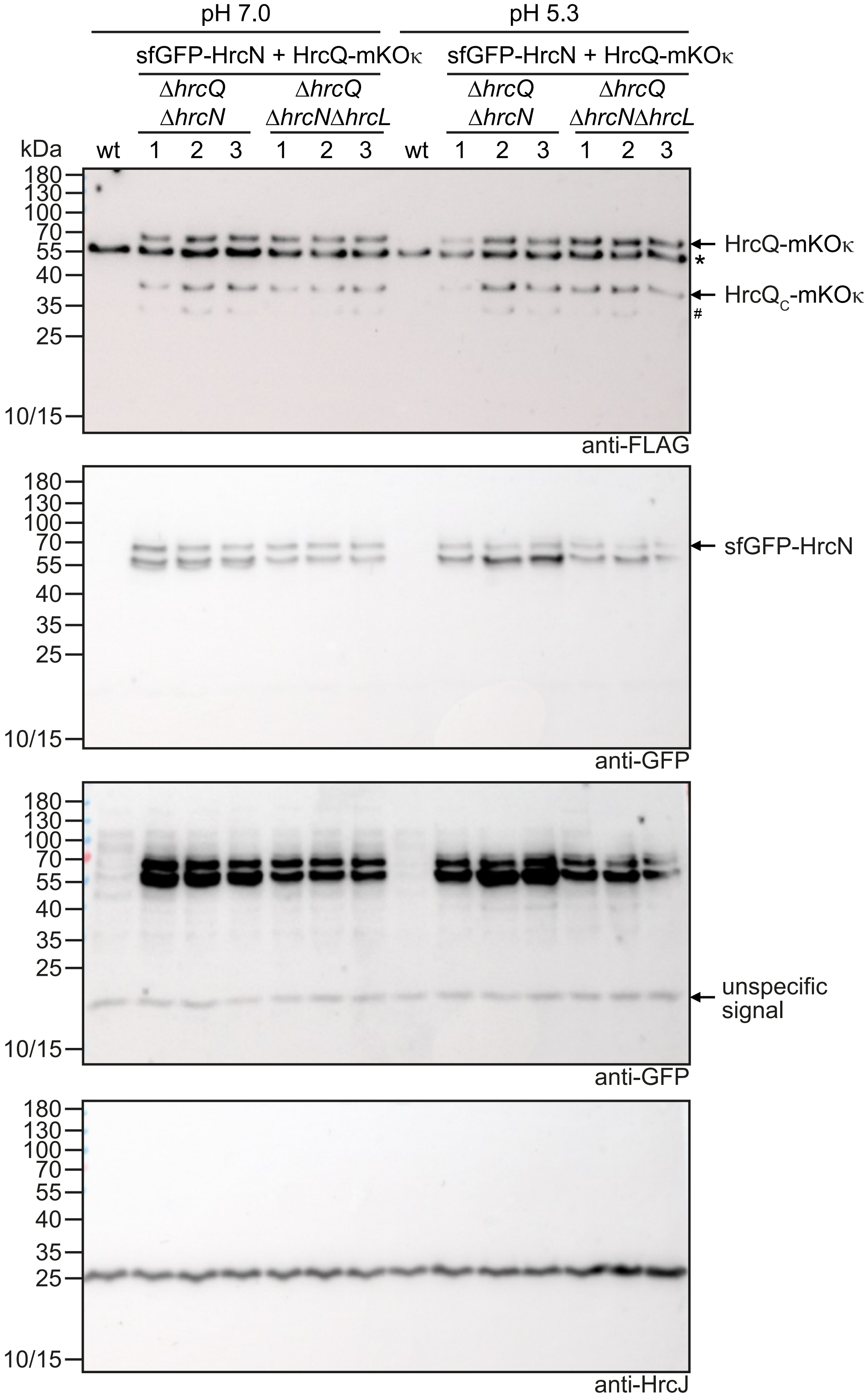

Figure S20
Otten and Büttner

Figure S20 HrcQ-mKO$_\kappa$ and sfGFP-HrcN are stably synthesized at pH 7.0 and pH 5.3. Immunological detection of HrcQ-mKO$_\kappa$ and sfGFP-HrcN in *Xe* cell extracts. Strain 85*Δ*hrp*_fs*HAGX* containing the wild-type (wt) modular T3S gene cluster or derivatives thereof with deletions in *hrcQ, hrcN* and *hrcL* (Δ*hrcQ*Δ*hrcN* or Δ*hrcQ*Δ*hrcN*Δ*hrcL*) and encoding HrcQ-mKO$_\kappa$ and sfGFP-HrcN as indicated were incubated in minimal medium under T3S-permissive (pH 5.3) and non-permissive (pH 7.0) conditions. Equal amounts of cell extracts from three transconjugants (labeled as 1, 2 and 3) of each strain were analysed by immunoblotting using antibodies specific for GFP, HrcJ or the FLAG epitope (which is present in the HrcQ-mKO$_\kappa$ fusion) as indicated. An unspecific signal detected by the FLAG-specific antibody demonstrates equal loading. Arrows indicate fusion proteins, degradation products are indicated with asterisks. Full, uncut immunoblots are shown. The signals shown in the uncut immunoblots on the right side for protein extracts from bacteria cultivated under T3S-permissive conditions at pH 5.3 correspond to the results presented in Fig. 7B.

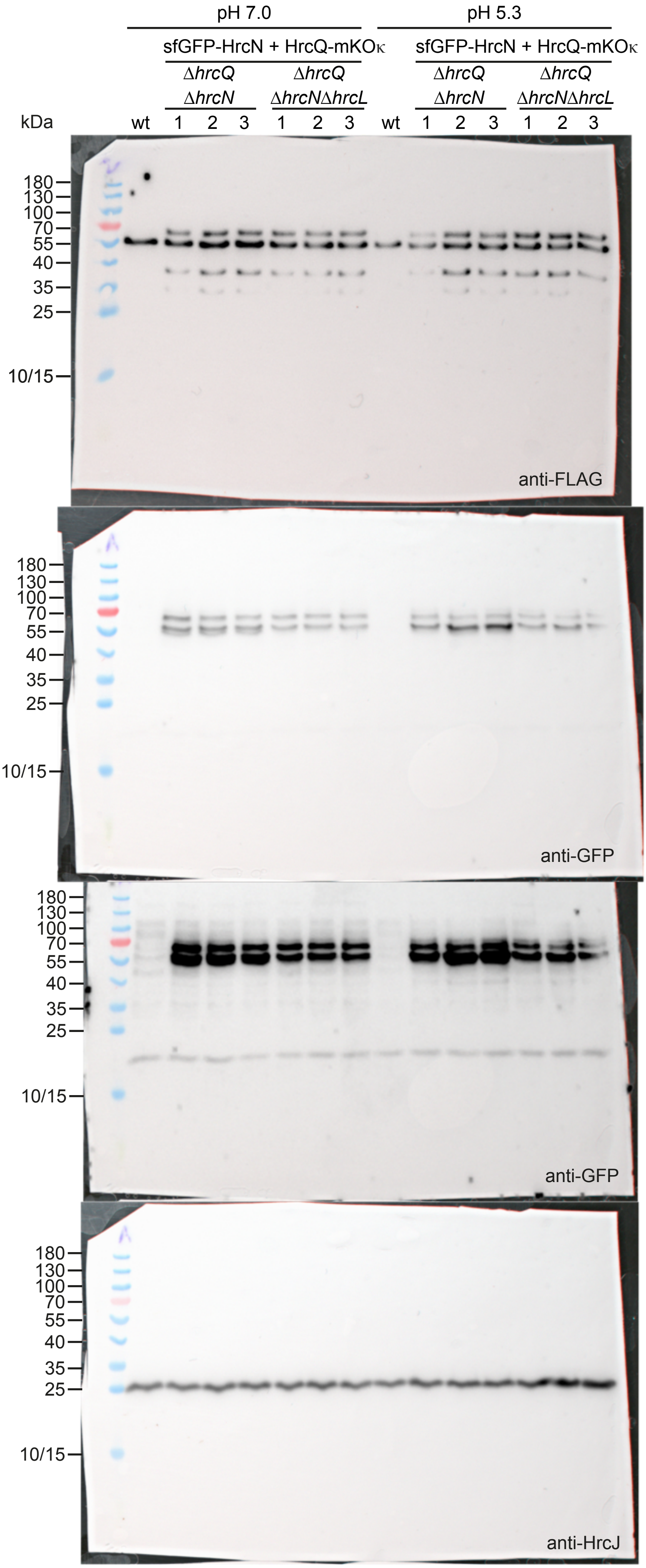

Figure S21

Otten and Büttner

Figure S21 Original blots for Figure S20

Strain 85*Δ*hrp*_fs*HAGX* containing the wild-type (wt) modular T3S gene cluster or derivatives thereof with deletions in *hrcQ, hrcN* and *hrcL* (Δ*hrcQ*Δ*hrcN* or Δ*hrcQ*Δ*hrcN*Δ*hrcL*) and encoding HrcQ-mKO$_{\kappa}$ and sfGFP-HrcN as indicated were incubated in minimal medium under T3S-permissive (pH 5.3) and non-permissive (pH 7.0) conditions. Equal amounts of cell extracts from three transconjugants (labeled as 1, 2 and 3) of each strain were analysed by immunoblotting using antibodies specific for GFP, HrcJ or the FLAG epitope (which is present in the HrcQ-mKO$_{\kappa}$ fusion) as indicated. Signals were detected using a chemiluminescence imager (Vilber Fusion FX Edge). Full, uncut immunoblots are shown.
